# Supplementary material for: Assessing the feasibility and impact of specially adapted exercise interventions, aimed at improving the multi-dimensional health and functional capacity of frail geriatric hospital inpatients: protocol for a feasibility study
Source: BMJ Open. 2019 Nov 21;9(11):e031159. doi: 10.1136/bmjopen-2019-031159 (PMC6886909; doi:10.1136/bmjopen-2019-031159)
Supplement: Supplementary data [file bmjopen-2019-031159supp001.pdf]

Leg Strength Assessment

Leg strength will be assessed by an estimated one repetition maximum (1RM) obtained from participants five repetition maximum (5RM) on the leg press and leg extension machines pre- and post-intervention. The protocol for this assessment can be found below:

Step 1. Participant performs a warm up on the machine with a light resistance that will easily allow 5 - 10 repetitions

↓

Step 2. 90 seconds rest

↓

Step 3. Based on Step 1, estimate a load that will easily allow 6 - 7 repetitions

↓

Step 4. 2 minutes rest

↓

Step 5. Estimate a load that will easily allow for 5 - 6 repetitions

↓

Step 6a. If the participant is successful, and both the researcher and the participant feel this the participants 5RM, use this value as the participants 5RM.

↓

Step 6b. If the participant is successful and feels as if they can add more weight, provide 2 minutes rest and add 5 - 10 % of the existing load.

↓

Step 6c. In the event a participant is unable to perform 5 repetions at the existing weight in Step 5, remove 5% of existing load and repeat Step 5.

Supplementary Material Figure 1: Five repetition maximum (5RM) assessment protocol adapted from Haff, G Gregory, Triplett, 2015
