# Supplementary material for: Assessing the feasibility and impact of specially adapted exercise interventions, aimed at improving the multi-dimensional health and functional capacity of frail geriatric hospital inpatients: protocol for a feasibility study
Source: BMJ Open. 2019 Nov 21;9(11):e031159. doi: 10.1136/bmjopen-2019-031159 (PMC6886909; doi:10.1136/bmjopen-2019-031159)
Supplement: Supplementary data [file bmjopen-2019-031159supp002.pdf]

Seated Physical Activity in Ageing (SPAA)
PANINI (Physical Activity and Nutritional INfluences In ageing) Project

UNIVERSITY OF BIRMINGHAM

Seated Physical Activity in Ageing (SPAA)

Participant Information Sheet

I. What is this study about?

This research study will assess whether it is feasible to conduct two seated physical activity interventions for frail older adults within a short-stay hospital ward setting. The intervention will run five days per week (35 mins per session) for two weeks. We are interested in the effects on health and wellbeing.

The specially-adapted chair-based physical activity interventions are:

Move It Or Lose It (MIOLI)

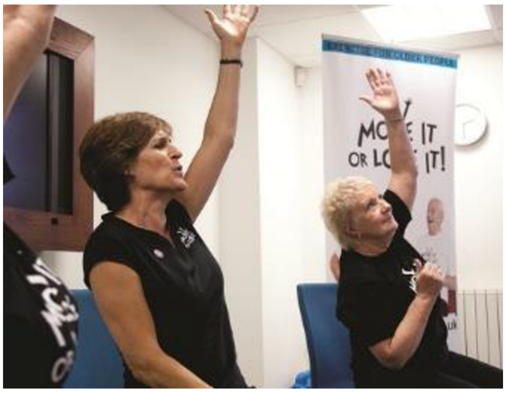

- An established chair-based physical activity programme for older adults.
- Exercises performed will be related to strength, balance, fitness and flexibility.
- The exercises are aimed to increase function and independence in older adults
- All exercises performed are chair-based, so they can be performed while sitting or standing (with, or without, chair support).

Resistance Training Intervention

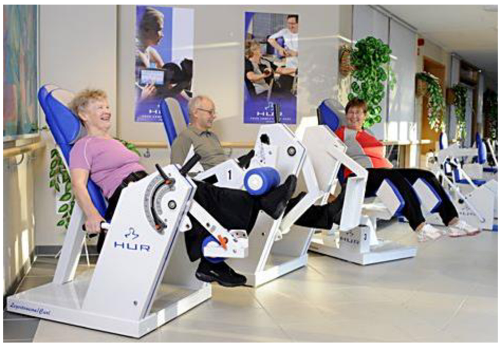

- Specially adapted resistance training machines for older adults.
- They are comfortable, easy to use, and are pneumatic (work on air-based resistance)
- They allow the difficulty to be increased in small amounts (100 grams - similar to the weight of a small apple).
- The machines work by scanning your wristband on a touch screen, and pressing buttons to increase or decrease difficulty

II. Who is organising and conducting this study?

The Physical Activity and Nutritional INfluences In ageing (PANINI) project research group at the University of Birmingham (School of Sport Exercise and Rehabilitation Sciences), in collaboration with the Queen Elizabeth Hospital Birmingham.

Research Team: Mr. Paul Doody (Email: p.d.doody@bham.ac.uk. Phone: 0121 414 4125)
Professor Anna Whittaker (Email: a.c.whittaker@bham.ac.uk. Phone: 0121 414 4121)
Professor Janet Lord (Email: j.m.lord@bham.ac.uk. Phone: 0121 371 3234)
Dr. Thomas Jackson PhD (Consultant Geriatrician) (Email: j.jackson@bham.ac.uk)
Dr. Zoe Wyrko (Consultant Geriatrician (Email: zoe.wyrko@uhb.nhs.uk)

Participant Information Sheet Version 4 – 10/01/2018 IRAS ID Number: 213954

III. Why have I been approached?

The Harborne ward is actively involved in research in geriatric populations, and encourages patient involvement in research to improve care.

In order to be eligible for the study, you must be:

- A patient / resident on the Harborne Ward
- ≥ 65 years old,
- Have the capacity to speak and read in English,
- Not currently terminally ill
- Not currently taking part in any other research study which could affect the findings of this present study
- Frail* (Possessing any three of the following criteria):
  - Low levels of handgrip strength,
  - Unintentional weight loss,
  - Low levels of physical activity,
  - Slow walking speed, and
  - Self-reported exhaustion

*If you would like to participate but are unsure if you are eligible based on the criteria above, do not worry, as these criteria will be assessed by the research team prior to enrolment once you have expressed your interest.

We will also liaise with your clinical care team to determine if you will be expected to stay on the ward for a length of time that would enable your participation in the study, and to ensure that you do not have any condition which could potentially exclude you from the study, such as severe sensory impairments.

IV. What will happen to me if I take part?

Your participation will be up to 3 weeks, including testing before and after the physical activity interventions themselves. There will be no changes to your standard care.

Before the intervention: Up to four days before the intervention, you will be asked to undergo a series of measurements including testing if you are eligible for the study (1 hour), relating to your physical, psychological, emotional and social health and physical ability. These will be assessed through tests, questionnaires, and a blood sample consisting of three small tubes (16ml total) to measure immune function. These will take about 3 hours in total, across two 1 hour 30-minute sessions over the four days. Although we would ideally like you to complete all of the assessments, if you feel it is too much, we will prioritise certain tests.

During the intervention: You will then be allocated into one of the two physical activity interventions (which intervention depends on the dates you are resident in the ward). You will be asked to complete 10 training sessions throughout two weeks within the physical activity intervention you have been allocated to. Each training session will last approximately 35 minutes, and all training sessions will take place on the Harborne ward.

After the Intervention: After the intervention, you will be asked to complete the same measurements as before (tests of function, questionnaires, and a blood sample) in order to assess the impact of the intervention, which again will be split into two sessions, each about 1 hour 30 minutes. You will also be asked to take part in an interview (20 minutes) with the researcher, audio-recorded with your permission. During this you will be able

UNIVERSITY OF BIRMINGHAM

to tell us your opinion of the intervention, what you enjoyed about it the study, and where it could be improved, which will allow us to adapt the intervention based on your feedback, which we would greatly appreciate. After transcription of the interviews the audio recording will be destroyed. The researchers within this study will also request limited access to your medical notes to allow the research team to assess any factors (injuries, medications, diseases) which might affect the results of the study. Information from your patient notes will be stored confidentially and separately from your personal details.

V. Do I have to take part?

Involvement is purely voluntary and you are free to withdraw from the study at any point without giving an explanation. There will be no negative consequences of withdrawing from the study in terms of your care. If you withdraw we would like to still analyse the data we have collected thus far.

If you express your interest in the study, a member of the research team will then meet with you to provide you with more information on the study, and to answer any questions you may have. If you states that you would not like to be involved in the research and would also not like to be contacted in the future with relation to participation, then you will not be approached by the researchers.

VI. What are the possible benefits of taking part?

Physical activity has been shown to produce positive health benefits in older people. Additionally, physical activity interventions have been suggested as potentially offering the best form of treatment for frail older adults. Frailty can have an enormous impact on an individual's life, in addition to the lives of their friends and family. If this current study proves feasible and is found to have a positive impact, this research has the potential to have positive implications in the advancement of the hospital care of frail older adults.

VII. What are the possible risks/side effects of taking part?

Physical activity in older people has a relatively small risk of adverse effects, typically mild muscle soreness. Due to the nature of the interventions (physical activity), it is possible that you may experience mild muscle soreness associated with physical activity, however this is a normal adaptation to exercise. In order to minimise the risk of Delayed Onset Muscle Soreness (DOMS – a more severe form of muscle soreness with a delayed onset), the interventions have been developed in a progressive manner. Additionally, the interventions will be carried out on the Harborne ward; with your care team in close proximity at all times.

VIII. Confidentiality and data protection

Your identity or other personal information will be kept confidential. You will be assigned an ID number under which all study information will be stored on an encrypted and password protected computer/laptop at the University of Birmingham. Physical data (e.g. questionnaires) will be identifiable only by ID number and stored in a locked filing cabinet at the University of Birmingham, accessible only by the research team. Your personal information (name, D.O.B.) and your consent form will be stored securely in a separate locked filing cabinet. Data will be stored securely and confidentially for 10 years and anonymized data will also be entered into a European 'PANINI' database that this project is part of. Blood samples will be stored in approved facilities at the University of Birmingham for three years and **optionally** may be analysed in future ethically approved research across the PANINI network. As part of the PANINI project we would like to send the smallest of the three blood sample tubes to the University of Bologna in Italy to be tested by specialists there for any potential alterations in genetic markers from the physical activity intervention (optional). The University of Birmingham has public liability insurance covering its research.

Seated Physical Activity in Ageing (SPAA)
PANINI (Physical Activity and Nutritional INfluences In ageing) Project

UNIVERSITY OF BIRMINGHAM

IX. Funding

This study has been funded by the European Commission as part of a European – wide innovate training network (ITN), consisting of Marie Curie Research Fellows / PhD students.

X. What next?

If you say that you are interested in taking part in the study to your clinical care team, the research team will come to answer any questions you may have, check you are eligible and enrol you into the study. If you do not state that you are either interested/not in the study, the research team will ask you about whether or not you would be potentially interested in taking part. If you would not like to take part, then please tell your care team and/or the research team and you will not be contacted further about participation.

If you agree to take part, we will ask you to sign an informed consent form, outlining that you have read this information sheet and that you are aware of what the study involves. You can either sign this straight away, or take up to 24 hours to make up your mind. After this, you will be assessed for the eligibility criteria, and then begin the testing as soon as possible and then start the intervention.

If you have concerns about this study please contact the study research team or your care team to discuss this. For independent advice, you can contact your local Patient Advice and Liaison Service (PALS) (0121 371 3280), or the study sponsor (the University of Birmingham, who have given this study the sponsor registration number: RG_16-172):

Dr. Sean Jennings, Head of Research Ethics and Governance, University of Birmingham, Edgbaston, Birmingham, United Kingdom. Telephone: 0121 415 8011, Email: s.jennings@bham.ac.uk

Thank you very much for your help
