# Supplementary material for: Assessing the feasibility and impact of specially adapted exercise interventions, aimed at improving the multi-dimensional health and functional capacity of frail geriatric hospital inpatients: protocol for a feasibility study
Source: BMJ Open. 2019 Nov 21;9(11):e031159. doi: 10.1136/bmjopen-2019-031159 (PMC6886909; doi:10.1136/bmjopen-2019-031159)
Supplement: Supplementary data [file bmjopen-2019-031159supp003.pdf]

Informed Consent Form
Seated Physical Activity in Ageing (SPAA)
PANINI (Physical Activity and Nutritional INfluences In ageing) Project

UNIVERSITY OF BIRMINGHAM

Seated Physical Activity in Ageing (SPAA)

II. Consent

Please complete the following table. Read each of the following statements. If you agree to the statement please put your **initials** in the box. If you do not agree to the statement please leave the box blank. Please note any boxes which are not marked as optional, are compulsory for initial enrollment into the study.

I confirm that I have read the Participant Information Sheet (Version 4 – 10/01/2018), and that I fully understand the information provided, and have had the opportunity to ask questions, and have received satisfactory answers.		
I understand that I will receive a unique ID number, under which all information collected about me will be stored confidentially, either on an encrypted and password protected laptop and computer, or in the case of physical information, within a locked filing cabinet, at the University of Birmingham, accessible only to the research team.		
I consent to giving a blood sample both pre- and post-intervention. I am aware that samples will be stored in a secure laboratory freezer at the University of Birmingham.		
I consent to giving information related to my personal data (name, age, occupation, marital status) psychological, cognitive, emotional, social health and functional capacity through questionnaires and tests.		
I agree to the audio-taping of an interview between myself and the researcher (where I can give my opinion on the study) and understand recordings will be destroyed after transcription.		
I understand that my data might be used in a written report, but will not be identifiable as me.		
I consent to all data obtained about me being stored for a period of 10 years as part of the PANINI project shared dataset, and that these data will be unidentifiable as mine but may be used in future ethically approved research. After 10 years it will be destroyed.		
I understand that involvement within this research project is purely voluntary and that I am free to withdraw from the study at any point which I wish to do so with no repercussion of any form for myself, or my quality of care.		
I understand that relevant sections of the data collected about me during the study may be looked at by ethics regulatory authorities or appropriate individuals from the University of Birmingham during a potential monitoring visit. I give permission for these individuals to have access to my linked anonymized data.		
I consent to take part in this above titled research study of my own volition		
**Optional (Please circle yes / no)** - I consent to members of the research team having limited access to my medical notes in order to allow information to be obtained relating to various morbidities (injuries, ailments, diseases) and medications.	Yes<br>No	
**Optional (Please circle yes / no)** - I consent to my blood samples being stored as part of the PANINI project shared dataset for three years, and that these data will be unidentifiable as mine, but may be used in future ethically approved research.	Yes<br>No	

Participants' Signature: ___________________

Name in Block Capitals: _________________

Date: __________________________________

Signature of person taking consent: _______________

Name in Block Capitals: ________________________

Date: ____

Seated Physical Activity in Ageing (SPAA)
Informed Consent Form – Version 4 10/01/2018 IRAS ID Number: 213954
