# Supplementary material for: Assessing the feasibility and impact of specially adapted exercise interventions, aimed at improving the multi-dimensional health and functional capacity of frail geriatric hospital inpatients: protocol for a feasibility study
Source: BMJ Open. 2019 Nov 21;9(11):e031159. doi: 10.1136/bmjopen-2019-031159 (PMC6886909; doi:10.1136/bmjopen-2019-031159)
Supplement: Author's manuscript [file bmjopen-2019-031159.draft_revisions.pdf]

BMJ Open

BMJ Open is committed to open peer review. As part of this commitment we make the peer review history of every article we publish publicly available.

When an article is published we post the peer reviewers' comments and the authors' responses online. We also post the versions of the paper that were used during peer review. These are the versions that the peer review comments apply to.

The versions of the paper that follow are the versions that were submitted during the peer review process. They are not the versions of record or the final published versions. They should not be cited or distributed as the published version of this manuscript.

BMJ Open is an open access journal and the full, final, typeset and author-corrected version of record of the manuscript is available on our site with no access controls, subscription charges or pay-per-view fees (http://bmjopen.bmj.com).

If you have any questions on BMJ Open's open peer review process please email info.bmjopen@bmj.com

BMJ Open

# Assessing the feasibility and impact of specially adapted exercise interventions, aimed at improving the multi-dimensional health and functional capacity of frail geriatric hospital inpatients: protocol for a feasibility study.

| | |
|---|---|
| Journal: | *BMJ Open* |
| Manuscript ID | bmjopen-2019-031159 |
| Article Type: | Protocol |
| Date Submitted by the Author: | 18-Apr-2019 |
| Complete List of Authors: | Doody, Paul; University of Birmingham, School of Sport, Exercise and Rehabilitation Sciences<br>Lord, Janet; University of Birmingham, NIHR Surgical Reconstruction & Microbiology Research Centre<br>Greig, Carolyn; School of Sport, Exercise and Rehabilitation Sciences, University of Birmingham, Edgbaston, Birmingham, B15 2TT, UK; MRC-Arthritis Research UK Centre for Musculoskeletal Ageing Research<br>Whittaker, Anna; University of Birmingham, School of Sport, Exercise and Rehabilitation Sciences; University of Stirling |
| Keywords: | Elderly, Exercise, Frail, Older adults, inpatient, geriatric |
| | |

SCHOLARONE™
Manuscripts

Protocol_Manscript_V1.0_25/03/2019

# Assessing the feasibility and impact of specially adapted exercise interventions, aimed at improving the multi-dimensional health and functional capacity of frail geriatric hospital inpatients: protocol for a feasibility study.

Paul Doody[1], Janet M. Lord[2,3], Carolyn A. Greig[1,2,3], Anna C. Whittaker[1,4]

[1] School of Sport, Exercise and Rehabilitation Sciences, University of Birmingham, United Kingdom

[2] MRC-Arthritis Research UK Centre for Musculoskeletal Ageing Research, Institute of Inflammation and Ageing, University of Birmingham, United Kingdom

[3] NIHR Birmingham Biomedical Research Centre, University Hospitals Birmingham NHS Foundation Trust and the University of Birmingham, United Kingdom

[4] Faculty of Health Sciences and Sport, University of Stirling, United Kingdom

Corresponding author: Mr. Paul Doody (p.d.doody@bham.ac.uk, +44 (0) 121 414 4125), School of Sport Exercise and Rehabilitation Sciences (G35), University of Birmingham, United Kingdom.

For peer review only

## Abstract

**Background:** Frailty is a common and clinically significant condition in older adults, predominantly due to its association with adverse health outcomes such as hospitalisation, disability and mortality. Exercise interventions have been shown to be a beneficial treatment for frailty. However, more high-quality studies are needed to assess the feasibility and impact of these interventions in frail geriatric populations within different settings, and their impact on broader aspects of health and wellbeing.

**Methods and analysis:** This study will utilise a 2-week, interventional, independent measures research design in order to assess the feasibility and impact of two specially adapted exercise training interventions; aimed at improving the multi-dimensional health and functional capacity of frail geriatric hospital inpatients.

**Ethics and dissemination:** This study has received a favourable ethical opinion by the Coventry and Warwickshire NHS Research Ethics Committee and sponsorship by the University of Birmingham after review by the sponsors research governance office. The findings will be disseminated through publication in open access scientific journals, public engagement events, online via social media, conference presentations, and directly to study participants upon request.

**Trial registration:** Clinicaltrials.gov: NCT03141866.


## Strengths and limitations

- Mixed methods feasibility study employing both quantitative and qualitative research methodologies
- Specially adapted exercise interventions for frail geriatric populations
- Difficult to reach (and often excluded) participant population
- Single site study
- **Key words:** Elderly; exercise; feasibility; frail; geriatric; hospital; inpatient; intervention; older adults; patient; physical activity.

For peer review only - http://bmjopen.bmj.com/site/about/guidelines.xhtml 

## **Background**

Frailty is a common and clinically significant condition within geriatric populations (Rodriguez-Mañas, Fried, 2015), predominantly due to its association with adverse health outcomes such as hospitalization, disability and mortality (Fried et al., 2001, Gill et al., 2006, Clegg et al., 2013, Sternberg et al., 2011, Sourial et al., 2013, Rodriguez-Mañas, Fried, 2015). Although the exact prevalence of frailty within this population is poorly defined due to the lack of a single standardised operational definition for the classification of frailty, it is generally believed that the prevalence of frailty amongst community-dwelling older adults ranges between 7.0 – 16.3% (Rodriguez-Mañas, Fried, 2015, Collard et al., 2012). Presently there are no well-evidenced, pooled estimates of the overall prevalence of frailty within geriatric hospital inpatients. However, there are several studies which have primarily aimed to produce estimates of the prevalence of frailty within this population (Joosten et al., 2014, Oliveira et al., 2013, Ekerstad et al., 2011, Purser et al., 2006, Khandelwal et al., 2012, Maguet et al., 2014, Hewitt et al., 2015, Andela et al., 2010). Although there is an evident need for more robust research to thoroughly assess the overall prevalence of frailty within this setting, through analysis of these existing studies, dependent upon the criteria utilised, the prevalence of frailty in geriatric hospital inpatients ranges from approximately 27 – 94%. In the five of the eight studies which utilised the Fried Frailty phenotype (Fried et al., 2001) as the operational definition of frailty, there is a narrower range (27–48.5%), with a mean prevalence of frailty across the five studies of 37.5±6.76% ((Joosten et al., 2014, Oliveira et al., 2013, Purser et al., 2006, Khandelwal et al., 2012, Le Maguet et al., 2014)).

Although there is no one standardised and universally utilised operational definition of frailty, one of the most commonly utilised is the Fried frailty phenotype (Fried et al., 2001). This proposes that frailty be defined as a clinical syndrome in which three or more of the five following criteria are present: unintentional weight loss (≥ 10lbs in the past year), self- reported exhaustion, weakness (grip strength), slow walking speed and low levels of physical activity.

For peer review only - http://bmjopen.bmj.com/site/about/guidelines.xhtml

Exercise interventions have been proposed as potentially offering the best form of treatment for frail older adults (Theou et al., 2011); with exercise shown to be a significantly beneficial treatment for this population (Fiatarone et al., 1994, Tarazona-Santabalbina et al., 2016, Arrieta et al., 2019); and even shown to mediate the reversal of frailty in some cases (Ng et al., 2016, Tarazona-Santabalbina et al., 2016). However, while there is evidence of the benefits of exercise relating to the prevention, treatment, and reversal of frailty, it is universally noted that there needs to be more high quality studies within this area to truly assess the impact of exercise in frail geriatric populations within different settings, particularly relating to its effects on broader aspects of health and well-being (Rodriguez-Mañas, Fried, 2015).

This present study will assess the feasibility and efficacy of short duration (2-week), intensive (five-days per week), specially adapted exercise interventions within a delayed transfer of care hospital ward setting. Feasibility will relate to the eight main areas of focus for feasibility studies (Bowen et al., 2009), while efficacy will be assessed through limited efficacy testing of the impact of the interventions on the secondary dependent variables relating to multi-dimensional health and functional capacity.

Such research is very timely and pertinent, as current demographic trends indicate that by the year 2030 almost one in six of the European population will be aged 60 or over, and the number of older people will grow to 247 million by 2050, representing a 35% increase from 2017, with one in four older adults being over 85 by 2040 (United Nations, Department of Economic and Social Affairs, Population Division, 2017). This coupled with continual progressive declines in the rate of physical activity, at all stages of the lifespan (Caspersen, Pereira & Curran, 2000), leaves the population particularly susceptible to the development of disease and co-morbidities associated with a lack of physical activity and an increase in sedentary behaviour (Ford, Caspersen, 2012). Moreover acute hospital admission for older adults are associated with further loss of physical activity and represent a period of increased susceptibility to sarcopenia and frailty (Liu et al.,

For peer review only - http://bmjopen.bmj.com/site/about/guidelines.xhtml

2018). Therefore, there is an urgent need to examine the effect of such interventions within this setting, and whether these interventions can be employed to improve various aspects of health in frail older populations in inpatient hospital ward settings, as well as their efficacy in specifically treating, preventing and reversing frailty.

## Methods and analysis

### Aims and objectives

The primary aim of this study is to assess the feasibility of a proposed future trial in this setting, which aims to assess the impact of specially adapted exercise interventions on the physiological, psychological, cognitive, social, and emotional health, and functional capacity of frail geriatric populations within a hospital ward setting; recognising health as a multi-factorial concept incorporating multiple inter-related dimensions. The secondary aim of this feasibility study is to assess the efficacy of the intervention on the primary dependent variables of the proposed future clinical trial within this setting.

The primary and secondary aims of this study will be achieved through the sequential achievement of the following objectives: 1) Recruitment of eligible participants from the Harborne Ward of the Queen Elizabeth Hospital Birmingham, Mindelsohn Way, Birmingham, United Kingdom. 2) Baseline assessment of the secondary dependent variables related to multi-dimensional health. 3) Assessment of the feasibility of the study as it relates to the eight-primary areas of focus for feasibility studies (Bowen et al., 2009). 4) Post-intervention assessment of all primary and secondary dependent variables.

The research questions of this study relate to the eight aforementioned areas of focus of this feasibility study, incorporating the following questions relating to the feasibility and efficacy of the study within this setting: Can it work? Will it work? Does it work? (Bowen et al., 2009) (Table 1).

### Design overview

For peer review only

This feasibility study will utilise a 2-week, interventional, independent measures research design (Figure 1).

*(Insert Figure 1)*

A variation of a stepped-wedged design / rolling recruitment will be utilised, with the interventions being conducted multiple times over the course of several months in order to maximise the potential sample size due to constraints of the setting: a delayed transfer of care hospital ward for patients prior to official discharge, with the majority of patients residing on the ward for > 3 weeks, and approximately 25 patients on the ward at any given time.

The independent variables of the study will comprise of two exercise training interventions: a specially adapted machine-based resistance training intervention, and Move It Or Lose It (MIOLI), an established community-based exercise intervention for older adults. A control group will be utilised within the proposed future clinical trial but will not be utilised within this feasibility study as one of the primary purposes of this study is to assess the feasibility, and to a limited degree the efficaciousness, of the interventions within this setting. (Figure 2). *(Insert Figure 2)*

In order to ensure this present study is as scientifically valid as possible a number of precautions have been taken to protect the internal and external validity of the study within its methodological design:

First, for each participant, all testing procedures (baseline and post-intervention (2-weeks) will be conducted at approximately the same time of day (+/- two hours). This will be controlled in order to protect the findings of the study from changes in the dependent variables which may be attributable to circadian variation rather than manipulation of the independent variables (Atkinson, Reilly, 1996). The hypothesis of the study will not be divulged to participants prior to or during the conduction of the study in order to control for any potential degree of demand characteristics; a scenario where participants alter their behaviour and/or answers, in order to align with what they believe is potentially the 'desired' outcome of the study. All dependent variable testing sessions will take place at least 24 hours after the cessation of the previous training session for each participant. This will be implemented to ensure acute fatigue does not become a

contributing factor to the results of the study, specifically relating to the secondary dependent variables, but also the feasibility of such practice during a proposed future clinical trial. The order in which dependent variables are tested will be counter-balanced throughout the study at each assessment timepoint in order to attempt to protect the study from practice effects, especially order effects, where a participant has been exposed to a specific order of testing before and as such performs better on subsequent testing procedures of the same material. Additionally, only one intervention will be run at a given time, protecting against potential subconscious selection bias amongst the research team relating to group allocation of participants. Finally, in order to increase the external validity of the study, eligibility criteria will be kept as minimalistic as possible (within the limits of safety and reason), in order to allow as inclusive a proportion of this population as possible, and in such producing findings which are applicable to not only those within the study, but to the greater population of frail geriatric hospital inpatients and particularly those within delayed transfer of care settings.

## Eligibility

This study is open to both men and woman whom meet the following eligibility criteria: inpatient on the Harborne ward of the Queen Elizabeth hospital Birmingham, Mindelsohn Way, Birmingham, United Kingdom; ≥ 65 years of age; frail according to the Fried Frailty Phenotype criteria (Fried et al., 2001); ability to speak and read in English; not currently taking part in any other clinical trial which could potentially impact upon or influence the findings of this study; not currently terminally ill with life expectancy less than the duration of the study; no severe sensory impairment which would profoundly impact upon ability to undergo the intervention, even once appropriate adaptations have been made; anticipated by their care team to remain on the ward for approximately 21 days post enrolment into the study.

## Interventions

For peer review only

All participants will undergo 10 sessions in total throughout the two-week intervention in either one of the two interventions. All session will be conducted individually under the guidance of a qualified trainer. A maximum of one session will be performed each day, and sessions will not be performed on any more than a maximum of three consecutive days throughout the duration of the study.

## Exercise Intervention 1: Specially-adapted resistance training intervention

This intervention will be comprised of an intensive (five days per week), short duration (2 week), 35 minutes per session, machine-based resistance training intervention. The exercises will specifically target the lower limbs; consisting of a leg press and leg extension machines (Figure 3). *(Insert Figure 3)*

An outline of the protocol for each session can be found in Figure 4. *(Insert Figure 4)*

## Exercise Intervention 2: Move It Or Lose It (MIOLI)

This intervention will comprise of an intensive (five days per week), short duration (2 week), 35 minutes per session, chair-based exercise intervention. The exercises conducted will relate to strength, power, flexibility and aerobic capacity, predominantly targeting the lower limbs, but also incorporating the upper body and core. An outline of the protocol for each session can be found in Figure 5. *(Insert Figure 5)*

## Dependent Variables

### Primary dependent variables

The primary dependent variables will relate to the eight primary areas of focus of feasibility studies (Bowen et al., 2009) (utilised to establish the feasibility of a proposed future clinical trial within this setting), relating to: acceptability, demand, implementation, practicality, adaptation, integration expansion and limited-efficacy testing.

For peer review only - http://bmjopen.bmj.com/site/about/guidelines.xhtml

These dependent variables will be assessed through semi-structured interviews with study participants and focus groups with ward staff post-intervention. Participant uptake and adherence records will also be employed throughout. These methods seek to attain answers to the following questions and parameters relating to the eight primary areas of enquiry for this feasibility study outlined in further detail in Table 1.

*(Insert Table 1 - Landscape)*

In order to enhance trustworthiness in the qualitative component of this research, several methods will be employed**:**

The researcher gathering the data will keep a reflective journal in which they will record information about themselves, their activities and the methods used. Field notes will include time, date and location, participant's actual notes, and the researcher's own questions and comments. This will lend to logging and documenting what is learned about the study, the intervention, the setting, the participants, and used to refine focus for future interviews through assessing the following questions: What is important? What is it I need to find out more about? What would I want to focus on more closely if I could do the interview again, or in future interviews? (Ortlipp, 2008).

Data will be gathered from study participants and ward staff in order to collect data from multiple sources (triangulate information). This study will also employ more than one researcher to analyse the qualitative data in order to enhance triangulation and validity.

Enough details will be given about the participants and the setting to make decisions about the quality of the findings from the qualitative analysis. Detailed descriptions about the participants experiences and the setting will be provided by the researcher.

In the qualitative data analysis, clarification of all possible researcher biases will be made known. For example, it will be articulated that the researcher is an advocate of physical activity as a means to promote health, prescribing to the theoretical and practical concept of exercise as medicine, and hence there may be some form of unconscious subjective bias in this context. However, it should also be noted that the

For peer review only

researcher within this study is also an advocate of science to an equal or even greater extent, and as such any such bias in subjective analysis would potentially be counteracted in this sense.

Interviews - "a conversation with a purpose" (Kahn, Cannell, 1957) will be the primary method of data gathering utilised, as it enables large amounts of information to be gathered relatively quickly. Specifically, this study will employ semi-structured interviews, with open questions in a conversational format. There will be several pre-determined themes, topics and questions to be discussed, specifically relating to the eight areas of focus of the primary dependent variables of this study. All interviews and focus groups will be audio-recorded in order to facilitate future transcription. The qualitative element of this study will also explore opportunities for Patient and Public Involvement (PPI) in the research design of the proposed future clinical trial.

As this study will utilise a mixed methods research approach, employing both qualitative and quantitative research methodologies, this will facilitate the potential for elaboration and expansion of these findings of individual methodologies through complementary analysis. The qualitative aspect of this feasibility study, aimed at assessing the primary dependent variables, will predominantly take a phenomenological approach to understand the experiences of individuals involved in the study (Smith, Osborn, 2004).

## Secondary Dependent Variables

The secondary dependent variables relating to multi-dimensional health (comprising the proposed primary dependent variables of the future clinical trial) will be:

Physiological*: Serum cortisol, dehydroepiandrosterone-sulphate (DHEAS), cortisol: DHEAS ratio, C-reactive protein (CRP), Interleukin 6 (IL-6), Tumor Necrosis Factor alpha (TNFα), Interferon gamma (IFNy)

Functional: Hand grip strength (Southampton protocol (Roberts et al., 2011)), Leg strength and power output (Haff, Triplett, 2015, Epley, 1985), Short Physical Performance Battery (SPPB) (Guralnik et al.,

For peer review only - http://bmjopen.bmj.com/site/about/guidelines.xhtml

1994), Katz Index of Independence in Activities of Daily Living (Katz ADL) (Katz, Chinn & Cordrey, 1959), and the Fried Frailty Phenotype (Fried et al., 2001)

Psychological / Emotional: Geriatric Depression Scale (GDS) (Yesavage et al., 1983), Hospital Anxiety Depression Scale (HADS) (Zigmond, Snaith, 1983)

Cognitive: Standardized Mini-Mental State Examination (SMMSE) (Molloy, Alemayehu & Roberts, 1991)

Social: Interpersonal Support Evaluation List (ISEL-12) (Cohen et al., 1985)

*All blood samples will be obtained through venepuncture. Serum will be analysed for the physiological dependent variables relating to cortisol, DHEAS and CRP (assessed by commercial ELISA kit). Inflammatory cytokines (IL-6, TNFα, IFNy) will be assayed using a multiplex commercial kit (R&D Systems).

For peer review only

## Data collection

Data will predominantly be collected at two main time points: baseline and post-intervention (Table 2).

*(Insert Table 2 - Landscape)*

**Baseline Assessment:** Participants' baseline socio-demographic and information for the secondary dependent variables will be collected between 5 – 2 days prior to intervention commencement. One repetition maximum (1RM) for the specially adapted resistance training equipment will also be assessed during this time period (after all baseline testing has been completed, and at least 24 hours after baseline testing which requires physical exertion, which may impact on the accuracy of the 1RM measurements).The resistance bands which participants in the MIOLI intervention will use will similarly be assessed during this period.

**Post-intervention assessment:** Primary and secondary dependent variables will be assessed between 1 – 5 days post intervention cessation. All assessments will be conducted at least 24 hours post the cessation of the last training session.

Adherence rates in the intervention will be recorded as the number of repetitions completed in a set (100% required for adherence to that exercise), and the number of exercises for which there was 100% adherence. If participants meet these parameters for each exercise session, they will be considered to be in 100% adherence to the intervention. For example, if a participant adheres to 100% of the intervention for 9 sessions, but only 90% for 1 session, then they will have a 99% adherence rate. Adherence rates, whether high, or low, may signify that the interventions may have been too demanding, too easy or optimal. Information will also be collected throughout the study related to uptake and retention rates.

## Data monitoring

Data will be monitored by the trial management committee at monthly intervals. Prior to analysis data entry checking will be conducted for accuracy on 10% of all participants, and queries resolved through discussion with the trial management committee and access to the source documents. Data management will adhere to the PANINI data management plan, which was developed in accordance with national and European principles as part of the university research governance and European Commission research governance guidelines. Thus, data management for this project adheres to the FAIR principles (Wilkinson et al., 2016).

## Sample size

This study aims to recruit a convenience sample of n = ~ 30 participants: 15 in each intervention. No formal power calculations were conducted due to the feasibility nature of this study. This sample size was based on initial expectations related to the obtainment of data saturation in the qualitative component of the study given its phenomenological approach (Morse, 1994, Creswell, Poth, 2017).

For peer review only

## Identification, Consent and Recruitment

**Identification:** Patients on the ward at the commencement of the study will initially be screened by their care team for the known presence of any severe sensory impairments which would exclude them from participation. Due to the study's rolling recruitment, this initial screening will continue throughout the study as additional patients are admitted to the ward. Participant information sheets will not be distributed to patients who are deemed to be medically unfit or due for imminent discharge as identified by their care team. All other aspects of the screening process will occur after consent has been obtained. After identification, potential participants will be approached by the researcher with an information sheet related to the study and asked if they would be interested in participating, or if they would like to receive more information about the study. The information sheet will contain all the most pertinent information relating to the study and specifically what it would require from potential participants. Potential participants will be given ≥ 24 hours to consider whether or not they would like to participate.

**Consent:** At this stage potential participants will also be provided with an informed consent form and asked if they would be interested in participating. If it is deemed that a potential participant lacks the capacity to consent, a personal consultee will be sought. If a personal consultee cannot be found, a nominated consultee will be sought. All efforts possible will be made in this regard to include participants whom lack the capacity to consent, as intrinsically within the research team from a personal and professional perspective we would consider it unethical to exclude potential participants from participating in a study, which can potentially benefit them and their overall health status, due to the fact that they lack the capacity to consent.

**Recruitment:** Following consent being obtained from the participant themselves, or the obtainment of a declaration from a consultee, all consented participants will be screened for the remaining eligibility criteria.

## Statistical methods

### Primary Dependent Variables

For peer review only - http://bmjopen.bmj.com/site/about/guidelines.xhtml

Analysis of the primary dependent variables will be based on an inductive process utilising Interpretative Phenomenological Analysis (thematic analysis). Two researchers will be employed to analyse the data to increase triangulation from the analysis perspective, having already triangulated data acquisition through obtainment from multiple sources (study participants and ward staff). All semi-structured interviews and focus groups will be audio-recorded. Data synthesis will be performed through verbatim transcription. The three main steps of Interpretative Phenomenological Analysis will be followed (Smith, Osborn, 2004): 1) The generation of themes from transcripts within the areas of feasibility inquiry. As an iterative process, these themes will be continuously reviewed and adapted based on the emergence of information in subsequent transcripts 2) The collation and separation of these themes within each of the areas of feasibility inquiry 3) Written interpretation of the resultant themes within each of the areas of feasibility and their relationship to one another. At all stages within this process, reflective journal entries and field notes will be utilised to provide a more comprehensive understanding of the findings, in addition to incorporating additional feasibility information related to uptake and retention rates, and limited efficacy testing of the secondary dependent variables in the final analysis to provide a comprehensive assessment of the feasibility of the study.

## Secondary Dependent Variables

Statistical analysis of the secondary dependent variables will be performed using IBM Statistical Package for Social Sciences (SPSS) software. Specifically, the following statistical analysis which will be utilised: 2x2 way independent measures ANOVA's (analysis' of variance consisting of two independent variables; the specially adapted resistance training intervention, and the Move It Or Lose It (MIOLI) intervention, each with two levels: baseline and post-intervention. A subsequent post-hoc test will be utilised if a significant main effect or interactions are found. Pearson product correlations will also be utilised between various socio-demographic variables (such as age and sex) and the dependent variables of this study, to assess

For peer review only

possible relationships between differences in socio-demographic factors and changes in the dependent variables.

Central tendency and variability measurements consisting of parameters such as the mean, median and mode, and standard deviation and range of scores respectively, will also be utilised during the analysis of data for illustrative purpose. Significance levels will be set at 0.05 ($p \leq 0.05$), and effect sizes will be reported for all analyses. In order to establish if the assumptions of parametric statistics have been met regarding the assumption that there is a normal distribution of data, the data will be analysed for skewness and kurtosis.

## Data storage and protection

Participants' identity or other personal information will be kept confidential. Participants will be assigned a unique ID number under which all study information will be stored in a secure file on an encrypted and password protected computer and laptop at the University of Birmingham (UoB). Physical data (e.g. Case Report Forms (CRF's)) will be identifiable only by ID number and stored in a locked filing cabinet at the School of Sport, Exercise and Rehabilitation Sciences at UOB, accessible only by the research team. Participants' personal data (name, date of birth) and consent forms matching them to their ID number, will be stored securely in a locked filing cabinet, separate from all other data and/or in a password protected master sheet on an encrypted and password protected computer and laptop at UOB. All serum samples will be stored in Human Tissue Act (HTA) compliant facilities at UOB for three years then destroyed.

All hard copy data collected on CRF's will be stored in a linked-anonymised format securely for 10 years then destroyed. All personal data (consent forms, master sheet linking participant IDs to names and contact details) will be stored for 10 years then destroyed. All computerised data will be archived on UOB servers in anonymised form for 10 years in the first instance in accordance with the UoB Code of Practice for Research, and the Data Protection Act (2018).

For peer review only

Following analysis for this specific study, all data will be anonymised and entered into a European 'PANINI' open access database that this project is part of and may be analysed in future ethically approved research across the PANINI network. The PANINI shared dataset will be made open access at the conclusion of the funding for the PANINI network including this study in 2020 and stored for at least 10 years as an open access searchable published dataset.

## Ethics and dissemination

### Ethical approval and consent to participate

This study has been granted a favourable ethical opinion by the Coventry and Warwickshire NHS Research Ethics Committee (REC) (17/WM/0390) on the 12/03/2018. This study has also been sponsored by the University of Birmingham, after review by the sponsor's research governance office (sponsor registration number: RG_17-108).

### Dissemination

The findings of this study will be disseminated through publication of scientific papers in open access scientific journals, public engagement events, online via social media (Twitter, Instagram) and the PANINI project website (Whittaker et al., 2018, http://www.birmingham.ac.uk/generic/panini/index.aspx), presentation at various conferences, and to study participants upon request.

### Safety reporting and monitoring

Adverse events (AE) and serious adverse events (SAE) will be monitored and if applicable recorded, by the primary researcher at each testing or exercise session and reported weekly to the principle investigator (PI) and reviewed in the case of AEs. SAEs will be reported immediately to the PI who will complete an SAE form

For peer review only

indicating causality and severity. The chief investigator (CI) will then submit this to the sponsor's research governance office, the Research Ethics Committee (REC) and University Hospitals Birmingham (UHB) research governance office, within 24 hours. UHB local policies and Standard Operating Procedures (SOP's) for all safety reporting will also be followed by the research team. SAEs related to pre-existing conditions will not be reported. Actions following an AE or SAE would be as standard, i.e. direct referral to their clinical care team, who then may wish to treat the AE or SAE themselves or where appropriate refer the participant to another relevant medical professional, and to recommend that the participant withdraw from the study unless they have been cleared to continue exercise by their attending physician.

For peer review only

## Trial registration

This study has been registered on ClinicalTrials.gov under the identifier number: NCT03141866.

## Trial status

This trial commenced on the 03/09/2018 on the Harborne ward of the Queen Elizabeth Hospital Birmingham, with anticipated completion of data collection scheduled for 06/08/2019.

## Funding

This review has been funded by the European Commission's Horizon 2020 research and innovation programme under the Marie Sklodowska-Curie grant agreement (675003); of which PD is a Marie Sklodowska-Curie Doctoral Research Fellow, AW, JL and CAG doctoral supervisors, and AW the grants Principal Investigator.

## Author contributions

PD designed the study protocol, and associated manuscript for publication, with supervision, input and feedback from AW at all stage of the design and writing process. JL and CAG reviewed and revised the manuscript prior to publication. All authors have read and approved the final manuscript.

## References

Andela, R.M., Dijkstra, A., Slaets, J.P. & Sanderman, R. 2010, "Prevalence of frailty on clinical wards: Description and implications", *International Journal of Nursing Practice,* vol. 16, no. 1, pp. 14-19.

Arrieta, H., Rezola-Pardo, C., Gil, S.M., Virgala, J., Iturburu, M., Antón, I., González-Templado, V., Irazusta, J. & Rodriguez-Larrad, A. 2019, "Effects of Multicomponent Exercise on Frailty in Long-Term Nursing Homes: A Randomized Controlled Trial", *Journal of the American Geriatrics Society,* .

Atkinson, G. & Reilly, T. 1996, "Circadian variation in sports performance", *Sports medicine,* vol. 21, no. 4, pp. 292-312.

Bowen, D.J., Kreuter, M., Spring, B., Cofta-Woerpel, L., Linnan, L., Weiner, D., Bakken, S., Kaplan, C.P., Squiers, L. & Fabrizio, C. 2009, "How we design feasibility studies", *American Journal of Preventive Medicine,* vol. 36, no. 5, pp. 452-457.

Caspersen, C.J., Pereira, M.A. & Curran, K.M. 2000, "Changes in physical activity patterns in the United States, by sex and cross-sectional age", *Medicine and science in sports and exercise,* vol. 32, no. 9, pp. 1601-1609.

Clegg, A., Young, J., Iliffe, S., Rikkert, M.O. & Rockwood, K. 2013, "Frailty in elderly people", *The Lancet,* vol. 381, no. 9868, pp. 752-762.

Cohen, S., Mermelstein, R., Kamarck, T. & Hoberman, H.M. 1985, "Measuring the functional components of social support" in *Social support: Theory, research and applications* Springer, , pp. 73-94.

Collard, R.M., Boter, H., Schoevers, R.A. & Oude Voshaar, R.C. 2012, "Prevalence of frailty in community-dwelling older persons: a systematic review", *Journal of the American Geriatrics Society,* vol. 60, no. 8, pp. 1487-1492.

Creswell, J.W. & Poth, C.N. 2017, *Qualitative inquiry and research design: Choosing among five approaches,* Sage publications.

For peer review only

Ekerstad, N., Swahn, E., Janzon, M., Alfredsson, J., Löfmark, R., Lindenberger, M. & Carlsson, P. 2011, "Frailty is independently associated with short-term outcomes for elderly patients with non-ST-segment elevation myocardial infarction", *Circulation,* vol. 124, no. 22, pp. 2397-2404.

Epley, B. 1985, "Poundage chart", *Boyd Epley Workout.Lincoln, NE: Body Enterprises, 2985,* vol. 86, pp. p. 86.

Fiatarone, M.A., O'Neill, E.F., Ryan, N.D., Clements, K.M., Solares, G.R., Nelson, M.E., Roberts, S.B., Kehayias, J.J., Lipsitz, L.A. & Evans, W.J. 1994, "Exercise training and nutritional supplementation for physical frailty in very elderly people", *New England Journal of Medicine,* vol. 330, no. 25, pp. 1769-1775.

Ford, E.S. & Caspersen, C.J. 2012, "Sedentary behaviour and cardiovascular disease: a review of prospective studies", *International journal of epidemiology,* , pp. dys078.

Fried, L.P., Tangen, C.M., Walston, J., Newman, A.B., Hirsch, C., Gottdiener, J., Seeman, T., Tracy, R., Kop, W.J. & Burke, G. 2001, "Frailty in older adults evidence for a phenotype", *The Journals of Gerontology Series A: Biological Sciences and Medical Sciences,* vol. 56, no. 3, pp. M157.

Gill, T.M., Gahbauer, E.A., Allore, H.G. & Han, L. 2006, "Transitions between frailty states among community-living older persons", *Archives of Internal Medicine,* vol. 166, no. 4, pp. 418-423.

Guralnik, J.M., Simonsick, E.M., Ferrucci, L., Glynn, R.J., Berkman, L.F., Blazer, D.G., Scherr, P.A. & Wallace, R.B. 1994, "A short physical performance battery assessing lower extremity function: association with self-reported disability and prediction of mortality and nursing home admission", *Journal of gerontology,* vol. 49, no. 2, pp. M94.

Haff, G. & Triplett, N.T. 2015, "Essentials of strength training and conditioning", , pp. 453.

Hewitt, J., Moug, S.J., Middleton, M., Chakrabarti, M., Stechman, M.J. & McCarthy, K. 2015, "Prevalence of frailty and its association with mortality in general surgery", *American Journal of Surgery,* vol. 209, no. 2, pp. 254-259.

Joosten, E., Demuynck, M., Detroyer, E. & Milisen, K. 2014a, "Prevalence of frailty and its ability to predict in hospital delirium, falls, and 6-month mortality in hospitalized older patients", *BMC Geriatrics,* vol. 14, no. 1.

Joosten, E., Demuynck, M., Detroyer, E. & Milisen, K. 2014b, "Prevalence of frailty and its ability to predict in hospital delirium, falls, and 6-month mortality in hospitalized older patients; 24393272", *BMC Geriatrics,* vol. 14, no. 1.

Kahn, R.L. & Cannell, C.F. 1957, "The dynamics of interviewing; theory, technique, and cases.", .

For peer review only

Katz, S., Chinn, A.B. & Cordrey, L. 1959, "Multidisciplinary studies of illness in aged persons II: a new classification of functional status in activities of daily living", *J Chronic Dis,* vol. 9, no. 1, pp. 55-62.

Khandelwal, D., Goel, A., Kumar, U., Gulati, V., Narang, R. & Dey, A.B. 2012a, "Frailty is associated with longer hospital stay and increased mortality in hospitalized older patients", *The journal of nutrition, health & aging,* vol. 16, no. 8, pp. 732-735.

Khandelwal, D., Goel, A., Kumar, U., Gulati, V., Narang, R. & Dey, A.B. 2012b, "Frailty is associated with longer hospital stay and increased mortality in hospitalized older patients", *Journal of Nutrition, Health and Aging,* , pp. 1-4.

Le Maguet, P., Roquilly, A., Lasocki, S., Asehnoune, K., Carise, E., Saint Martin, M., Mimoz, O., Le Gac, G., Somme, D., Cattenoz, C., Feuillet, F., Malledant, Y. & Seguin, P. 2014, "Prevalence and impact of frailty on mortality in elderly ICU patients: A prospective, multicenter, observational study", *Intensive care medicine,* vol. 40, no. 5, pp. 674-682.

Liu, J., Reijnierse, E.M., Ancum, J., Verlaan, S., Meskers, C. & Maier, A.B. 2018, "Acute inflammation is associated with lower muscle strength, muscle mass and dependency in activities of daily living in male hospitalised older patients", *AUSTRALASIAN JOURNAL ON AGEING*WILEY 111 RIVER ST, HOBOKEN 07030-5774, NJ USA, , pp. 58.

Maguet, P.L., Roquilly, A., Lasocki, S., Asehnoune, K., Carise, E., Martin, M.S., Mimoz, O., Gac, G.L., Somme, D., Cattenoz, C., Feuillet, F., Malledant, Y. & Seguin, P. 2014, "Prevalence and impact of frailty on mortality in elderly ICU patients: a prospective, multicenter, observational study", *Intensive Care Medicine,* vol. 40, no. 5, pp. 674-682.

Molloy, D.W., Alemayehu, E. & Roberts, R. 1991, "Reliability of a standardized mini-mental state examination compared with the traditional mini-mental state examination", *Am J Psychiatry,* vol. 148, no. 1, pp. 102-105.

Morse, J.M. 1994, "Designing funded qualitative research.", .

Ng, J.K.-., Kwan, B.C.-., Chow, K.-., Cheng, P.M.-., Law, M.-., Pang, W.-., Leung, C.-., Li, P.K.-. & Szeto, C.-. 2016, "Frailty in Chinese Peritoneal Dialysis Patients: Prevalence and Prognostic Significance", *Kidney and Blood Pressure Research,* vol. 41, no. 6, pp. 736-745.

Oliveira, D.R., Bettinelli, L.A., Pasqualotti, A., Corso, D., Brock, F. & Erdmann, A.L. 2013a, "Prevalence of frailty syndrome in old people in a hospital institution", *Revista latino-americana de enfermagem,* vol. 21, no. 4, pp. 891-898.

Oliveira, D.R., Bettinelli, L.A., Pasqualotti, A., Corso, D., Brock, F., Erdmann, A.L., Oliveira, D.R., Bettinelli, L.A., Pasqualotti, A., Corso, D., Brock, F. & Erdmann, A.L.

For peer review only

2013b, "Prevalence of frailty syndrome in old people in a hospital institution", *Revista Latino-Americana de Enfermagem,* vol. 21, no. 4, pp. 891-898.

Ortlipp, M. 2008, "Keeping and using reflective journals in the qualitative research process", *The qualitative report,* vol. 13, no. 4, pp. 695-705.

Purser, J.L., Kuchibhatla, M.N., Fillenbaum, G.G., Harding, T., Peterson, E.D. & Alexander, K.P. 2006a, "Identifying frailty in hospitalized older adults with significant coronary artery disease", *Journal of the American Geriatrics Society,* vol. 54, no. 11, pp. 1674-1681.

Purser, J.L., Kuchibhatla, M.N., Fillenbaum, G.G., Harding, T., Peterson, E.D. & Alexander, K.P. 2006b, "Identifying Frailty in Hospitalized Older Adults with Significant Coronary Artery Disease", *Journal of the American Geriatrics Society,* vol. 54, no. 11, pp. 1674-1681.

Roberts, H.C., Denison, H.J., Martin, H.J., Patel, H.P., Syddall, H., Cooper, C. & Sayer, A.A. 2011, "A review of the measurement of grip strength in clinical and epidemiological studies: towards a standardised approach", *Age and Ageing,* vol. 40, no. 4, pp. 423-429.

Rodriguez-Mañas, L. & Fried, L.P. 2015, "Frailty in the clinical scenario", *The Lancet,* vol. 385, no. 9968, pp. e9.

Smith, J.A. & Osborn, M. 2004, "Interpretative phenomenological analysis", *Doing social psychology research,* , pp. 229-254.

Sourial, N., Bergman, H., Karunananthan, S., Wolfson, C., Payette, H., Gutierrez-Robledo, L.M., Béland, F., Fletcher, J.D. & Guralnik, J. 2013, "Implementing Frailty Into Clinical Practice: A Cautionary Tale", *The Journals of Gerontology Series A: Biological Sciences and Medical Sciences,* , pp. glt053.

Sternberg, S.A., Schwartz, A.W., Karunananthan, S., Bergman, H. & Mark Clarfield, A. 2011, "The identification of frailty: a systematic literature review", *Journal of the American Geriatrics Society,* vol. 59, no. 11, pp. 2129-2138.

Tarazona-Santabalbina, F.J., Gmez-Cabrera, M.C., Prez-Ros, P., Martnez-Arnau, F.M., Cabo, H., Tsaparas, K., Salvador-Pascual, A., Rodriguez-Maas, L. & Via, J. 2016, "A multicomponent exercise intervention that reverses frailty and improves cognition, emotion, and social networking in the community-dwelling frail elderly: a randomized clinical trial", *Journal of the American Medical Directors Association,* vol. 17, no. 5, pp. 426-433.

Theou, O., Stathokostas, L., Roland, K.P., Jakobi, J.M., Patterson, C., Vandervoort, A.A. & Jones, G.R. 2011, "The effectiveness of exercise interventions for the management of frailty: a systematic review", *Journal of aging research,* vol. 2011.

For peer review only - http://bmjopen.bmj.com/site/about/guidelines.xhtml 

United Nations, Department of Economic and Social Affairs, Population Division *World Population Ageing 2017 (ST/ESA/SER.A/408). URL: https://www.un.org/en/development/desa/population/publications/pdf/ageing/WPA2017_Report.pdf. Accessed: 17th April 2019.*

University of Birmingham *, Physical Activity and Nutritional INfluences In ageing (PANINI) *. Available: http://www.birmingham.ac.uk/generic/panini/index.aspx [2019, 6th February].

Whittaker, A.C., Delledonne, M., Finni, T., Garagnani, P., Greig, C., Kallen, V., Kokko, K., Lord, J., Maier, A.B. & Meskers, C.G. 2018, "Physical Activity and Nutrition INfluences In ageing (PANINI): consortium mission statement", *Aging clinical and experimental research,* , pp. 1-8.

Wilkinson, M.D., Dumontier, M., Aalbersberg, I.J., Appleton, G., Axton, M., Baak, A., Blomberg, N., Boiten, J., da Silva Santos, Luiz Bonino & Bourne, P.E. 2016, "The FAIR Guiding Principles for scientific data management and stewardship", *Scientific data,* vol. 3.

Yesavage, J.A., Brink, T.L., Rose, T.L., Lum, O., Huang, V., Adey, M. & Leirer, V.O. 1983, "Development and validation of a geriatric depression screening scale: a preliminary report", *Journal of psychiatric research,* vol. 17, no. 1, pp. 37-49.

Zigmond, A.S. & Snaith, R.P. 1983, "The hospital anxiety and depression scale", *Acta Psychiatrica Scandinavica,* vol. 67, no. 6, pp. 361-370.

For peer review only

# Tables

*Table 1. The eight primary areas of focus, outlining the research questions and methods of assessment*

| Area of focus | Potential questions | Methods of Assessment |
|---|---|---|
| Acceptability | • Will the proposed population be interested in participating in the study?<br>• What will the uptake be?<br>• Will the program be judged as suitable by the delivers of the program in addition to the program participants?<br>• Participant's opinions on hypothetically being randomized into a control group during a proposed future clinical trial? *<br><br>*Participants within the feasibility study will not be recruited as participants within the proposed future clinical trial in order to protect the scientific validity of the proposed future clinical trial, as the participants within the feasibility will already have undergone the interventions, or similar interventions. However, participants within the feasibility study will likely not still be residents on the ward, as the average stay for participants on the ward is approximately 3.5 weeks. As such once the proposed future clinical trial has commenced, all participants within the feasibility trial will likely have left the ward well in advance. | • Participant uptake analysis (All participants approached and eligible for the study, all of those successfully recruited to the study)<br>• Semi-structured interviews with participants<br>• Focus groups with study support staff. |
| Demand | • Will the proposed population of hospital inpatients participate in the study?<br>• What will adherence rates be?<br>• Are the staff on the ward open to the idea of having exercise interventions potentially on the ward long term if it proves effective? | • Analysis of uptake rates<br>• Exercise intervention adherence rates<br>• Focus groups with study support staff/ward staff. |
| Implementation | • What are the possible logistical issues with the setting which will need to be addressed or accounted for prior to the clinical trial?<br>• Can the interventions be successfully carried out within this setting?<br>• Can a single or double bind be successfully implemented within this setting? | • Semi-structured interviews with study participants.<br>• More in-depth with focus groups with study support staff. |
| Practicality | • What are the practical implications of the study with relation to time commitment of the researchers, relating to both the implementation of the interventions, and the testing of participants for the dependent variables of the proposed future clinical trial?<br>• Is it viable to potentially conduct follow-up testing on participants in the proposed future clinical trial? | • Semi-structured interviews with study participants.<br>• Focus groups with support staff. |

For peer review only

| | | |
|---|---|---|
| | • Do any alterations need to be made to the proposed primary dependent variables of the future clinical trial?<br>• If the interventions are successful in influencing parameters of health and functional capacity, will it potentially be possible to assess if these improvements are sustained during a two week follow up in the proposed future clinical trial, if the same is found? | |
| Integration | • How will the ward staff appraise the study?<br>• Will the interventions be easily integrated into the existing culture, protocols and procedures within the ward seamlessly? | • Focus groups with ward/support staff. |
| Adaptation | • Will any further adaptations be required to the existing interventions to make them more feasible or appropriate within this setting? | • Semi structured interviews with participants. |
| Expansion | • Can the Move It or Lose It intervention (an established chair-based exercise programme for older adults) be successfully expanded to this setting?<br>• Can be specially adapted resistance training equipment be successfully expanded to this setting? | • Semi-structured interviews / Focus group with the ward/study support staff. |
| Limited-efficacy testing | • Is two weeks a sufficient duration to potentially provide significant benefit to patients?<br>• Can intensive (five-six days per week), short duration (two weeks) physical activity interventions improve markers of multi-dimensional health, in very frail individuals? | • Analysis of the secondary dependent variables within the study (primary dependant variables of the future clinical trial)<br>• Analysis of uptake and adherence rates<br>• Analysis of the level of satisfaction with the interventions through questionnaires with participants post intervention. |

566

567

568

For peer review only

*Table 2. Study timeline of all major events throughout the duration of the study (SPIRIT Schedule)*

| Week | Seated Physical Activity in Ageing (SPAA) Study Timeline | | | | | | |
|---|---|---|---|---|---|---|---|
| | Monday | Tuesday | Wednesday | Thursday | Friday | Saturday | Sunday |
| Week 0 | Identification + information sheet distribution | 24-hour consideration period | Recruitment, eligibility screening, and pre-intervention assessments | Recruitment, eligibility screening, and pre-intervention assessments | Recruitment, eligibility screening, and pre-intervention assessments | Recruitment, eligibility screening, and pre-intervention assessments | - |
| Week 1 | Training | Rest | Training | Training | Rest | Training | Training |
| Week 2 | Training | Rest | Training | Training | Training | Rest | Training |
| Week 3 | Post-Intervention Assessment | Post-Intervention Assessment | Post-Intervention Assessment | Post-Intervention Assessment | Post-Intervention Assessment | Post-Intervention Assessment | - |



For peer review only - http://bmjopen.bmj.com/site/about/guidelines.xhtml

# Figures

Figure 1. Trial schema of participant flow throughout the duration of the study.

Figure 2. The independent variables of the proposed future clinical trial.

Figure 3. Minato resistance training equipment utilised within the specially adapted resistance training intervention.

Figure 4. Exercise intervention 1- Specially adapted resistance training intervention protocol.

Figure 5. Exercise intervention 2 – Move It Or Lose It (MIOLI) chair-based exercise intervention protocol.

All figures are attached to this manuscript submission as separate documents titled “Figure 1”, “Figure 2”, “Figure 3”, “Figure 4” and “Figure 5” accordingly.

For peer review only

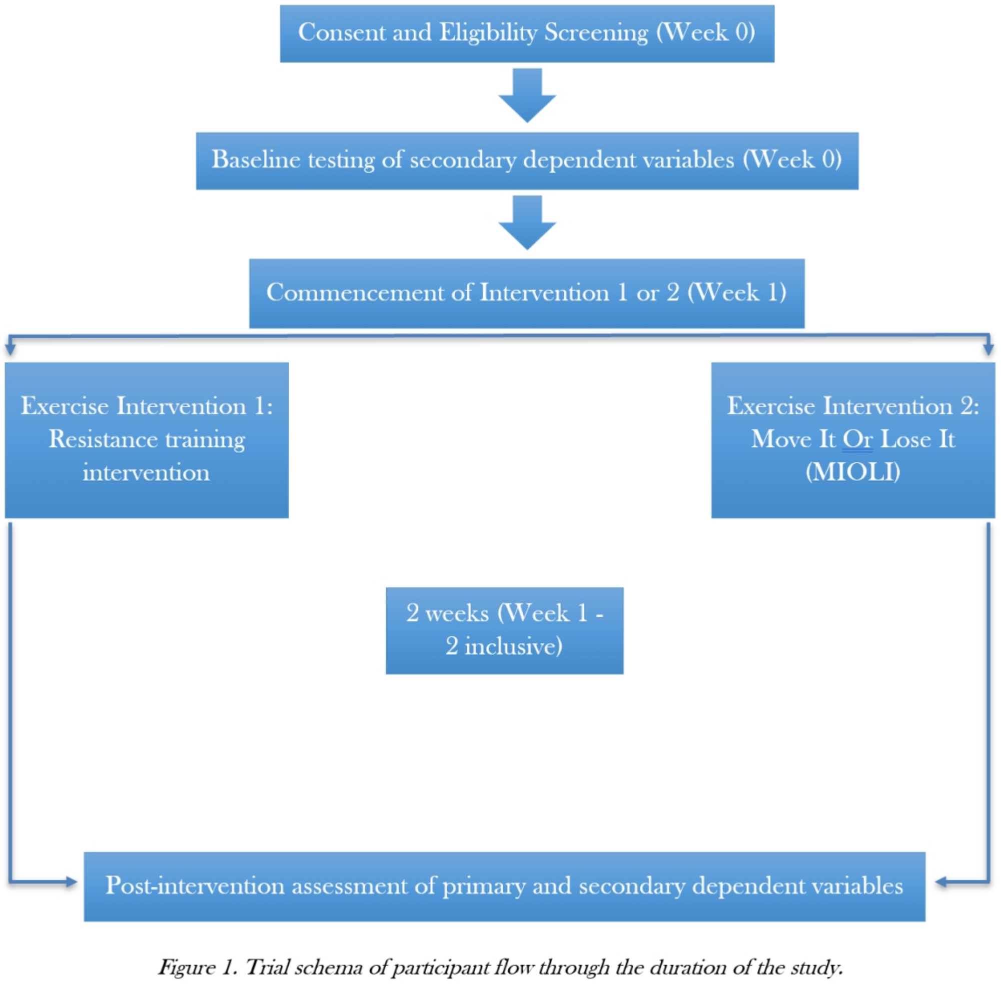


Figure 1. Trial schema of participant flow through the duration of the study.

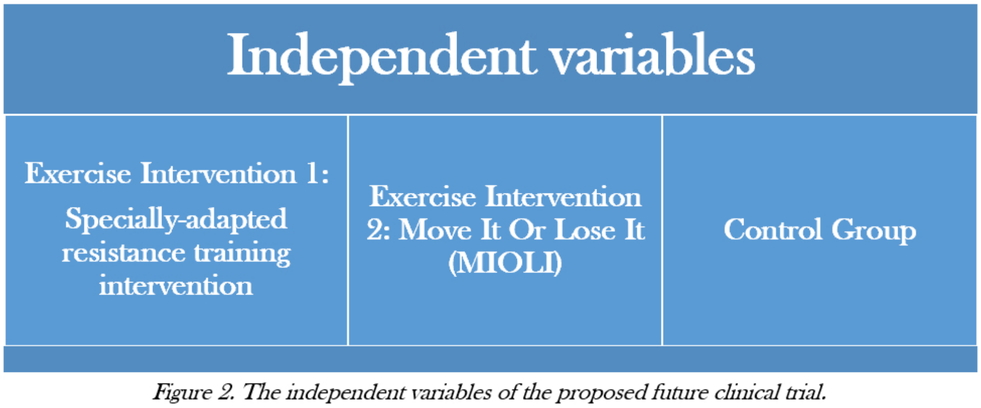


Figure 2. The independent variables of the proposed future clinical trial.

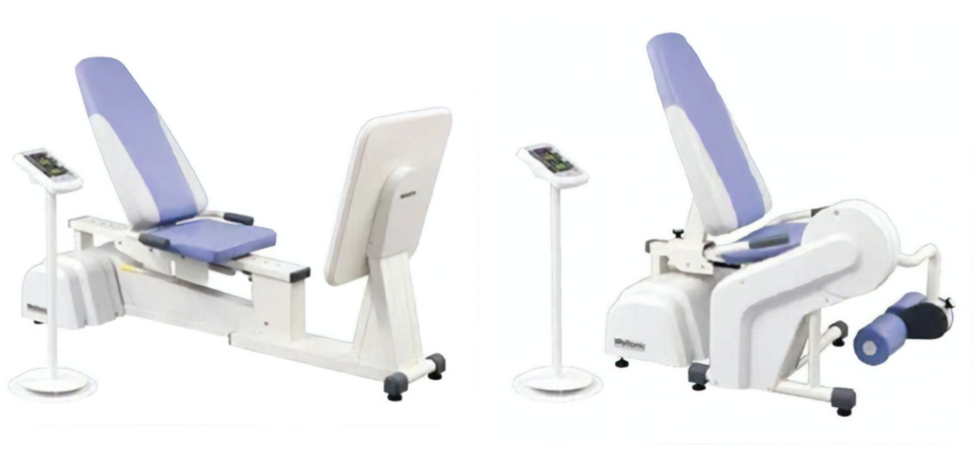

*Figure 3. Minato resistance training equipment utilised within the specially adapted resistance training intervention*

Figure 3. Minato resistance trianing equipment utilised within the speically adapted resistence training intervention.

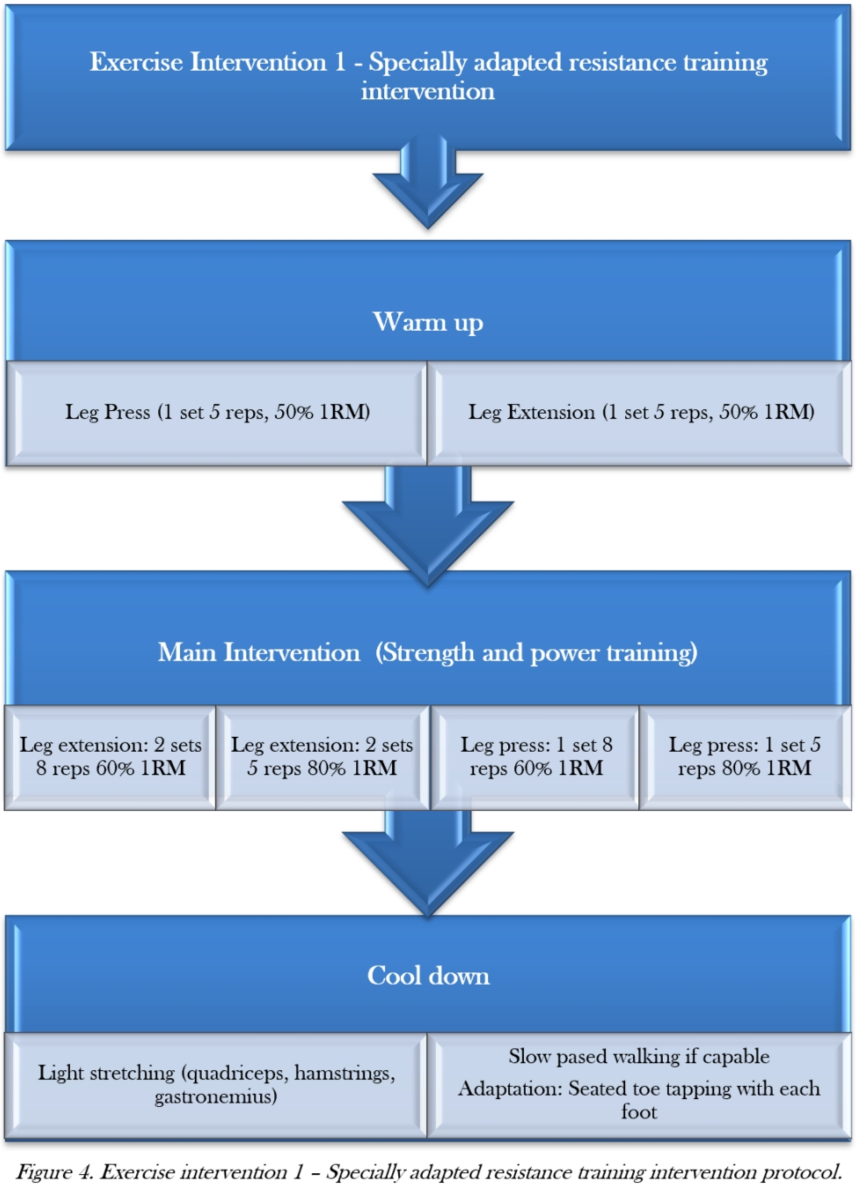


Figure 4. Exercise intervention 1 - Specially adapted resistance training intervention.

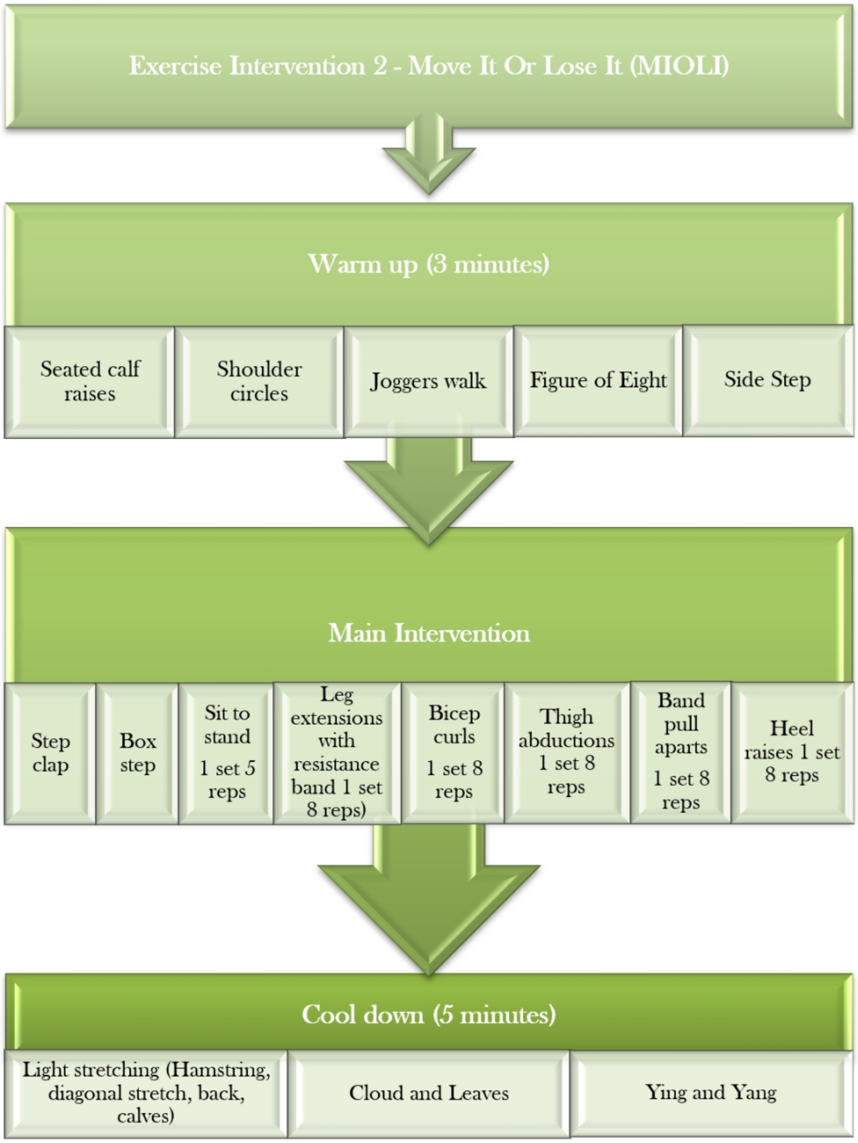


*Figure 5. Exercise Intervention 2 – Move It Or Lose It (MIOLI) chair-based exercise intervention protocol.*

Figure 5. Exercise Intervention 2 - Move It Or Lose It (MIOLI) chair-based exercise intervention protocol.

# Seated Physical Activity in Ageing (SPAA)

## Participant Information Sheet

## I. What is this study about?

This research study will assess whether it is feasible to conduct two seated physical activity interventions for frail older adults within a short-stay hospital ward setting. The intervention will run five days per week (35 mins per session) for two weeks. We are interested in the effects on health and wellbeing.

The specially-adapted chair-based physical activity interventions are:

### Move It Or Lose It (MIOLI)

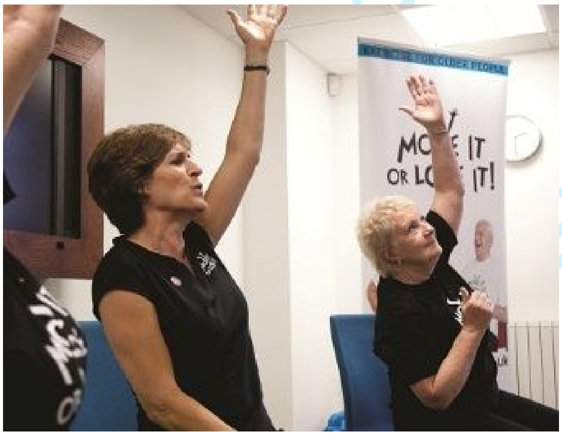

- An established chair-based physical activity programme for older adults.
- Exercises performed will be related to strength, balance, fitness and flexibility.
- The exercises are aimed to increase function and independence in older adults
- All exercises performed are chair-based, so they can be performed while sitting or standing (with, or without, chair support).

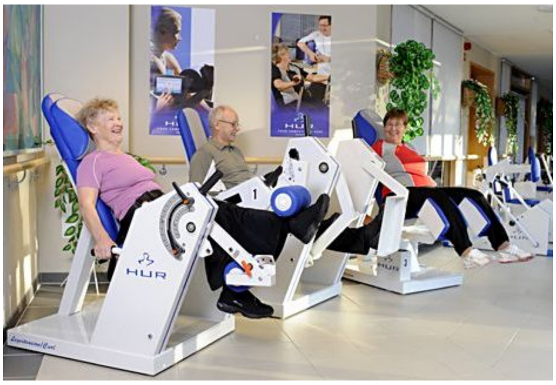


### Resistance Training Intervention

- Specially adapted resistance training machines for older adults.
- They are comfortable, easy to use, and are pneumatic (work on air-based resistance)
- They allow the difficulty to be increased in small amounts (100 grams - similar to the weight of a small apple).
- The machines work by scanning your wristband on a touch screen, and pressing buttons to increase or decrease difficulty

## II. Who is organising and conducting this study?

The Physical Activity and Nutritional INfluences In ageing (PANINI) project research group at the University of Birmingham (School of Sport Exercise and Rehabilitation Sciences), in collaboration with the Queen

Elizabeth Hospital Birmingham.

Research Team: Mr. Paul Doody (Email: p.d.doody@bham.ac.uk. Phone: 0121 414 4125)
Professor Anna Whittaker (Email: a.c.whittaker@bham.ac.uk. Phone: 0121 414 4121)
Professor Janet Lord (Email: j.m.lord@bham.ac.uk. Phone: 0121 371 3234)



For peer review only - http://bmjopen.bmj.com/site/about/guidelines.xhtml

Seated Physical Activity in Ageing (SPAA)
PANINI (Physical Activity and Nutritional INfluences In ageing) Project
UNIVERSITY OF BIRMINGHAM
Dr. Thomas Jackson PhD (Consultant Geriatrician) (Email: j.jackson@bham.ac.uk)
Dr. Zoe Wyrko (Consultant Geriatrician (Email: zoe.wyrko@uhb.nhs.uk)

## III. Why have I been approached?

The Harborne ward is actively involved in research in geriatric populations, and encourages patient involvement in research to improve care.

In order to be eligible for the study, you must be:

- A patient / resident on the Harborne Ward
- ≥ 65 years old,
- Have the capacity to speak and read in English,
- Not currently terminally ill
- Not currently taking part in any other research study which could affect the findings of this present study
- Frail* (Possessing any three of the following criteria):
  - Low levels of handgrip strength,
  - Unintentional weight loss,
  - Low levels of physical activity,
  - Slow walking speed, and
  - Self-reported exhaustion

*If you would like to participate but are unsure if you are eligible based on the criteria above, do not worry, as these criteria will be assessed by the research team prior to enrolment once you have expressed your interest.

We will also liaise with your clinical care team to determine if you will be expected to stay on the ward for a length of time that would enable your participation in the study, and to ensure that you do not have any condition which could potentially exclude you from the study, such as severe sensory impairments.

## IV. What will happen to me if I take part?

Your participation will be up to 3 weeks, including testing before and after the physical activity interventions themselves. There will be no changes to your standard care.

**Before the intervention:** Up to four days before the intervention, you will be asked to undergo a series of measurements including testing if you are eligible for the study (1 hour), relating to your physical, psychological, emotional and social health and physical ability. These will be assessed through tests, questionnaires, and a blood sample consisting of three small tubes (16ml total) to measure immune function. These will take about 3 hours in total, across two 1 hour 30-minute sessions over the four days. Although we would ideally like you to complete all of the assessments, if you feel it is too much, we will prioritise certain tests.

**During the intervention:** You will then be allocated into one of the two physical activity interventions (which intervention depends on the dates you are resident in the ward). You will be asked to complete 10 training sessions throughout two weeks within the physical activity intervention you have been allocated to. Each training session will last approximately 35 minutes, and all training sessions will take place on the Harborne ward.


For peer review only - http://bmjopen.bmj.com/site/about/guidelines.xhtml

**After the Intervention:** After the intervention, you will be asked to complete the same measurements as before (tests of function, questionnaires, and a blood sample) in order to assess the impact of the intervention, which again will be split into two sessions, each about 1 hour 30 minutes. You will also be asked to take part in an interview (20 minutes) with the researcher, audio-recorded with your permission. During this you will be able to tell us your opinion of the intervention, what you enjoyed about it the study, and where it could be improved, which will allow us to adapt the intervention based on your feedback, which we would greatly appreciate. After transcription of the interviews the audio recording will be destroyed. The researchers within this study will also request limited access to your medical notes to allow the research team to assess any factors (injuries, medications, diseases) which might affect the results of the study. Information from your patient notes will be stored confidentially and separately from your personal details.

## V. Do I have to take part?

Involvement is purely voluntary and you are free to withdraw from the study at any point without giving an explanation. There will be no negative consequences of withdrawing from the study in terms of your care. If you withdraw we would like to still analyse the data we have collected thus far.

If you express your interest in the study, a member of the research team will then meet with you to provide you with more information on the study, and to answer any questions you may have. If you states that you would not like to be involved in the research and would also not like to be contacted in the future with relation to participation, then you will not be approached by the researchers.

## VI. What are the possible benefits of taking part?

Physical activity has been shown to produce positive health benefits in older people. Additionally, physical activity interventions have been suggested as potentially offering the best form of treatment for frail older adults. Frailty can have an enormous impact on an individual's life, in addition to the lives of their friends and family. If this current study proves feasible and is found to have a positive impact, this research has the potential to have positive implications in the advancement of the hospital care of frail older adults.

## VII. What are the possible risks/side effects of taking part?

Physical activity in older people has a relatively small risk of adverse effects, typically mild muscle soreness. Due to the nature of the interventions (physical activity), it is possible that you may experience mild muscle soreness associated with physical activity, however this is a normal adaptation to exercise. In order to minimise the risk of Delayed Onset Muscle Soreness (DOMS – a more severe form of muscle soreness with a delayed onset), the interventions have been developed in a progressive manner. Additionally, the interventions will be carried out on the Harborne ward; with your care team in close proximity at all times.

## VIII. Confidentiality and data protection

Your identity or other personal information will be kept confidential. You will be assigned an ID number under which all study information will be stored on an encrypted and password protected computer/laptop at the University of Birmingham. Physical data (e.g. questionnaires) will be identifiable only by ID number and stored in a locked filing cabinet at the University of Birmingham, accessible only by the research team. Your personal information (name, D.O.B.) and your consent form will be stored securely in a separate locked filing cabinet. Data will be stored securely and confidentially for 10 years and anonymized data will also be entered into a European 'PANINI' database that this project is part of. Blood samples will be stored in approved facilities at the University of Birmingham for three years and **optionally** may be analysed in future ethically approved research across the PANINI network. As part of the PANINI project we would like to send the smallest of the three blood sample tubes to the University of Bologna in Italy to be tested by specialists there


For peer review only - http://bmjopen.bmj.com/site/about/guidelines.xhtml

for any potential alterations in genetic markers from the physical activity intervention (optional). The University of Birmingham has public liability insurance covering its research.

## IX. Funding

This study has been funded by the European Commission as part of a European – wide innovate training network (ITN), consisting of Marie Curie Research Fellows / PhD students.

## X. What next?

If you say that you are interested in taking part in the study to your clinical care team, the research team will come to answer any questions you may have, check you are eligible and enrol you into the study. If you do not state that you are either interested/not in the study, the research team will ask you about whether or not you would be potentially interested in taking part. If you would not like to take part, then please tell your care team and/or the research team and you will not be contacted further about participation.

If you agree to take part, we will ask you to sign an informed consent form, outlining that you have read this information sheet and that you are aware of what the study involves. You can either sign this straight away, or take up to 24 hours to make up your mind. After this, you will be assessed for the eligibility criteria, and then begin the testing as soon as possible and then start the intervention.

If you have concerns about this study please contact the study research team or your care team to discuss this. For independent advice, you can contact your local Patient Advice and Liaison Service (PALS) (0121 371 3280), or the study sponsor (the University of Birmingham, who have given this study the sponsor registration number: RG_16-172):

Dr. Sean Jennings, Head of Research Ethics and Governance, University of Birmingham, Edgbaston, Birmingham, United Kingdom. Telephone: 0121 415 8011, Email: s.jennings@bham.ac.uk

Thank you very much for your help

For peer review only


For peer review only - http://bmjopen.bmj.com/site/about/guidelines.xhtml

Informed Consent Form
Seated Physical Activity in Ageing (SPAA)
PANINI (Physical Activity and Nutritional INfluences In ageing) Project

UNIVERSITY OF BIRMINGHAM

# Seated Physical Activity in Ageing (SPAA)

## II. Consent

Please complete the following table. Read each of the following statements. If you agree to the statement please put your **initials** in the box. If you do not agree to the statement please leave the box blank. Please note any boxes which are not marked as optional, are compulsory for initial enrollment into the study.

| | | |
|---|---|---|
| I confirm that I have read the Participant Information Sheet (Version 4 – 10/01/2018), and that I fully understand the information provided, and have had the opportunity to ask questions, and have received satisfactory answers. | | |
| I understand that I will receive a unique ID number, under which all information collected about me will be stored confidentially, either on an encrypted and password protected laptop and computer, or in the case of physical information, within a locked filing cabinet, at the University of Birmingham, accessible only to the research team. | | |
| I consent to giving a blood sample both pre- and post-intervention. I am aware that samples will be stored in a secure laboratory freezer at the University of Birmingham. | | |
| I consent to giving information related to my personal data (name, age, occupation, marital status) psychological, cognitive, emotional, social health and functional capacity through questionnaires and tests. | | |
| I agree to the audio-taping of an interview between myself and the researcher (where I can give my opinion on the study) and understand recordings will be destroyed after transcription. | | |
| I understand that my data might be used in a written report, but will not be identifiable as me. | | |
| I consent to all data obtained about me being stored for a period of 10 years as part of the PANINI project shared dataset, and that these data will be unidentifiable as mine but may be used in future ethically approved research. After 10 years it will be destroyed. | | |
| I understand that involvement within this research project is purely voluntary and that I am free to withdraw from the study at any point which I wish to do so with no repercussion of any form for myself, or my quality of care. | | |
| I understand that relevant sections of the data collected about me during the study may be looked at by ethics regulatory authorities or appropriate individuals from the University of Birmingham during a potential monitoring visit. I give permission for these individuals to have access to my linked anonymized data. | | |
| I consent to take part in this above titled research study of my own volition | | |
| **Optional (Please circle yes / no)** - I consent to members of the research team having limited access to my medical notes in order to allow information to be obtained relating to various morbidities (injuries, ailments, diseases) and medications. | Yes<br><br>No | |
| **Optional (Please circle yes / no)** - I consent to my blood samples being stored as part of the PANINI project shared dataset for three years, and that these data will be unidentifiable as mine, but may be used in future ethically approved research. | Yes<br><br>No | |

**Participants' Signature:** ____________________

**Name in Block Capitals:** ____________________

**Date:** ____________________

**Signature of person taking consent:** ____________________

**Name in Block Capitals:** ____________________

**Date:** ____________________



For peer review only - http://bmjopen.bmj.com/site/about/guidelines.xhtml

SPIRIT

STANDARD PROTOCOL ITEMS: RECOMMENDATIONS FOR INTERVENTIONAL TRIALS

SPIRIT 2013 Checklist: Recommended items to address in a clinical trial protocol and related documents*

| Section/item | Item No | Description | Addressed on page number |
|---|---|---|---|
| **Administrative information** | | | |
| Title | 1 | Descriptive title identifying the study design, population, interventions, and, if applicable, trial acronym | 1 |
| Trial registration | 2a | Trial identifier and registry name. If not yet registered, name of intended registry | 2 |
| | 2b | All items from the World Health Organization Trial Registration Data Set | |
| Protocol version | 3 | Date and version identifier | 1 |
| Funding | 4 | Sources and types of financial, material, and other support | 17 |
| Roles and responsibilities | 5a | Names, affiliations, and roles of protocol contributors | 1, 17, 18 |
| | 5b | Name and contact information for the trial sponsor | 16 |
| | 5c | Role of study sponsor and funders, if any, in study design; collection, management, analysis, and interpretation of data; writing of the report; and the decision to submit the report for publication, including whether they will have ultimate authority over any of these activities | N/A |
| | 5d | Composition, roles, and responsibilities of the coordinating centre, steering committee, endpoint adjudication committee, data management team, and other individuals or groups overseeing the trial, if applicable (see Item 21a for data monitoring committee) | 12 |

For peer review only



For peer review only - http://bmjopen.bmj.com/site/about/guidelines.xhtml

**Introduction**

| | | | |
|---|---|---|---|
| Background and rationale | 6a | Description of research question and justification for undertaking the trial, including summary of relevant studies (published and unpublished) examining benefits and harms for each intervention | 3-5 |
| | 6b | Explanation for choice of comparators | 5 |
| Objectives | 7 | Specific objectives or hypotheses | 5 |
| Trial design | 8 | Description of trial design including type of trial (eg, parallel group, crossover, factorial, single group), allocation ratio, and framework (eg, superiority, equivalence, noninferiority, exploratory) | 6,7 |

**Methods: Participants, interventions, and outcomes**

| | | | |
|---|---|---|---|
| Study setting | 9 | Description of study settings (eg, community clinic, academic hospital) and list of countries where data will be collected. Reference to where list of study sites can be obtained | 5-7 |
| Eligibility criteria | 10 | Inclusion and exclusion criteria for participants. If applicable, eligibility criteria for study centres and individuals who will perform the interventions (eg, surgeons, psychotherapists) | 7 |
| Interventions | 11a | Interventions for each group with sufficient detail to allow replication, including how and when they will be administered | 8 |
| | 11b | Criteria for discontinuing or modifying allocated interventions for a given trial participant (eg, drug dose change in response to harms, participant request, or improving/worsening disease) | 16, 17 |
| | 11c | Strategies to improve adherence to intervention protocols, and any procedures for monitoring adherence (eg, drug tablet return, laboratory tests) | 12 |
| | 11d | Relevant concomitant care and interventions that are permitted or prohibited during the trial | 6, 7 |
| Outcomes | 12 | Primary, secondary, and other outcomes, including the specific measurement variable (eg, systolic blood pressure), analysis metric (eg, change from baseline, final value, time to event), method of aggregation (eg, median, proportion), and time point for each outcome. Explanation of the clinical relevance of chosen efficacy and harm outcomes is strongly recommended | 8-11 |
| Participant timeline | 13 | Time schedule of enrolment, interventions (including any run-ins and washouts), assessments, and visits for participants. A schematic diagram is highly recommended (see Figure) | 11,12 |



For peer review only - http://bmjopen.bmj.com/site/about/guidelines.xhtml

| | | | |
|---|---|---|---|
| Sample size | 14 | Estimated number of participants needed to achieve study objectives and how it was determined, including clinical and statistical assumptions supporting any sample size calculations | 12 |
| Recruitment | 15 | Strategies for achieving adequate participant enrolment to reach target sample size | 13 |
| **Methods: Assignment of interventions (for controlled trials)** | | | |
| Allocation: | | | |
| Sequence generation | 16a | Method of generating the allocation sequence (eg, computer-generated random numbers), and list of any factors for stratification. To reduce predictability of a random sequence, details of any planned restriction (eg, blocking) should be provided in a separate document that is unavailable to those who enrol participants or assign interventions | 7 |
| Allocation concealment mechanism | 16b | Mechanism of implementing the allocation sequence (eg, central telephone; sequentially numbered, opaque, sealed envelopes), describing any steps to conceal the sequence until interventions are assigned | |
| Implementation | 16c | Who will generate the allocation sequence, who will enrol participants, and who will assign participants to interventions | |
| Blinding (masking) | 17a | Who will be blinded after assignment to interventions (eg, trial participants, care providers, outcome assessors, data analysts), and how | |
| | 17b | If blinded, circumstances under which unblinding is permissible, and procedure for revealing a participant's allocated intervention during the trial | |
| **Methods: Data collection, management, and analysis** | | | |
| Data collection methods | 18a | Plans for assessment and collection of outcome, baseline, and other trial data, including any related processes to promote data quality (eg, duplicate measurements, training of assessors) and a description of study instruments (eg, questionnaires, laboratory tests) along with their reliability and validity, if known. Reference to where data collection forms can be found, if not in the protocol | 8-12 |
| | 18b | Plans to promote participant retention and complete follow-up, including list of any outcome data to be collected for participants who discontinue or deviate from intervention protocols | |

| | | | |
|---|---|---|---|
| Data management | 19 | Plans for data entry, coding, security, and storage, including any related processes to promote data quality (eg, double data entry; range checks for data values). Reference to where details of data management procedures can be found, if not in the protocol | 12,15, 16 |
| Statistical methods | 20a | Statistical methods for analysing primary and secondary outcomes. Reference to where other details of the statistical analysis plan can be found, if not in the protocol | 14, 15 |
| | 20b | Methods for any additional analyses (eg, subgroup and adjusted analyses) | |
| | 20c | Definition of analysis population relating to protocol non-adherence (eg, as randomised analysis), and any statistical methods to handle missing data (eg, multiple imputation) | |
| **Methods: Monitoring** | | | |
| Data monitoring | 21a | Composition of data monitoring committee (DMC); summary of its role and reporting structure; statement of whether it is independent from the sponsor and competing interests; and reference to where further details about its charter can be found, if not in the protocol. Alternatively, an explanation of why a DMC is not needed | 12 |
| | 21b | Description of any interim analyses and stopping guidelines, including who will have access to these interim results and make the final decision to terminate the trial | |
| Harms | 22 | Plans for collecting, assessing, reporting, and managing solicited and spontaneously reported adverse events and other unintended effects of trial interventions or trial conduct | 16, 17 |
| Auditing | 23 | Frequency and procedures for auditing trial conduct, if any, and whether the process will be independent from investigators and the sponsor | |
| **Ethics and dissemination** | | | |
| Research ethics approval | 24 | Plans for seeking research ethics committee/institutional review board (REC/IRB) approval | 16 |
| Protocol amendments | 25 | Plans for communicating important protocol modifications (eg, changes to eligibility criteria, outcomes, analyses) to relevant parties (eg, investigators, REC/IRBs, trial participants, trial registries, journals, regulators) | |

| | | | |
|---|---|---|---|
| Consent or assent | 26a | Who will obtain informed consent or assent from potential trial participants or authorised surrogates, and how (see Item 32) | 13 |
| | 26b | Additional consent provisions for collection and use of participant data and biological specimens in ancillary studies, if applicable | 13,15, 16 |
| Confidentiality | 27 | How personal information about potential and enrolled participants will be collected, shared, and maintained in order to protect confidentiality before, during, and after the trial | 15, 16 |
| Declaration of interests | 28 | Financial and other competing interests for principal investigators for the overall trial and each study site | 17 |
| Access to data | 29 | Statement of who will have access to the final trial dataset, and disclosure of contractual agreements that limit such access for investigators | 15, 16 |
| Ancillary and post-trial care | 30 | Provisions, if any, for ancillary and post-trial care, and for compensation to those who suffer harm from trial participation | |
| Dissemination policy | 31a | Plans for investigators and sponsor to communicate trial results to participants, healthcare professionals, the public, and other relevant groups (eg, via publication, reporting in results databases, or other data sharing arrangements), including any publication restrictions | 16 |
| | 31b | Authorship eligibility guidelines and any intended use of professional writers | 18 |
| | 31c | Plans, if any, for granting public access to the full protocol, participant-level dataset, and statistical code | 16 |
| **Appendices** | | | |
| Informed consent materials | 32 | Model consent form and other related documentation given to participants and authorised surrogates | |
| Biological specimens | 33 | Plans for collection, laboratory evaluation, and storage of biological specimens for genetic or molecular analysis in the current trial and for future use in ancillary studies, if applicable | 15, 16 |

*It is strongly recommended that this checklist be read in conjunction with the SPIRIT 2013 Explanation & Elaboration for important clarification on the items. Amendments to the protocol should be tracked and dated. The SPIRIT checklist is copyrighted by the SPIRIT Group under the Creative Commons "Attribution-NonCommercial-NoDerivs 3.0 Unported" license.

For peer review only - http://bmjopen.bmj.com/site/about/guidelines.xhtml

BMJ Open

# Assessing the feasibility and impact of specially adapted exercise interventions, aimed at improving the multi-dimensional health and functional capacity of frail geriatric hospital inpatients: protocol for a feasibility study.



| | |
|---|---|
| Journal: | *BMJ Open* |
| Manuscript ID | bmjopen-2019-031159.R1 |
| Article Type: | Protocol |
| Date Submitted by the Author: | 26-Jul-2019 |



| | |
|---|---|
| Complete List of Authors: | Doody, Paul; University of Birmingham, School of Sport, Exercise and Rehabilitation Sciences<br>Lord, Janet; University of Birmingham, NIHR Surgical Reconstruction & Microbiology Research Centre<br>Greig, Carolyn; School of Sport, Exercise and Rehabilitation Sciences, University of Birmingham, Edgbaston, Birmingham, B15 2TT, UK; MRC-Arthritis Research UK Centre for Musculoskeletal Ageing Research<br>Whittaker, Anna; University of Birmingham, School of Sport, Exercise and Rehabilitation Sciences; University of Stirling |



| | |
|---|---|
| <b>Primary Subject Heading</b>: | Sports and exercise medicine |
| Secondary Subject Heading: | Geriatric medicine, Rehabilitation medicine |
| Keywords: | Elderly, Exercise, Frail, Older adults, inpatient, geriatric |
| | |


SCHOLARONE™
Manuscripts

Protocol_Manscript_V2.0_11/07/2019

# Assessing the feasibility and impact of specially adapted exercise interventions, aimed at improving the multi-dimensional health and functional capacity of frail geriatric hospital inpatients: protocol for a feasibility study.

Paul Doody[1], Janet M. Lord[2,3], Carolyn A. Greig[1,2,3], Anna C. Whittaker[1,4]

[1] School of Sport, Exercise and Rehabilitation Sciences, University of Birmingham, United Kingdom

[2] MRC-Arthritis Research UK Centre for Musculoskeletal Ageing Research, Institute of Inflammation and Ageing, University of Birmingham, United Kingdom

[3] NIHR Birmingham Biomedical Research Centre, University Hospitals Birmingham NHS Foundation Trust and the University of Birmingham, United Kingdom

[4] Faculty of Health Sciences and Sport, University of Stirling, United Kingdom

Corresponding author: Mr. Paul Doody (p.d.doody@bham.ac.uk, +44 (0) 121 414 4125), School of Sport Exercise and Rehabilitation Sciences (G35), University of Birmingham, United Kingdom.

For peer review only

Protocol_Manscript_V2.0_11/07/2019

## Abstract

**Background:** Frailty is a common and clinically significant condition in older adults, predominantly due to its association with adverse health outcomes such as hospitalisation, disability and mortality. Exercise interventions have been shown to be a beneficial treatment for frailty. However, more high-quality studies are needed to assess the feasibility and impact of these interventions in frail geriatric populations within different settings, and their impact on broader aspects of health and wellbeing.

**Methods and analysis:** This study will utilise a 2-week, interventional, independent measures research design in order to assess the feasibility and impact of two specially adapted exercise training interventions (a specially adapted resistance training intervention, and Move It Or Lose It: an established community-based exercise intervention for older adults) aimed at improving the multi-dimensional health and functional capacity of frail geriatric hospital inpatients.

**Ethics and dissemination:** This study has received a favourable ethical opinion by the Coventry and Warwickshire NHS Research Ethics Committee and sponsorship by the University of Birmingham after review by the sponsors research governance office. The findings will be disseminated through publication in open access scientific journals, public engagement events, online via social media, conference presentations, and directly to study participants upon request.

**Trial registration:** Clinicaltrials.gov: NCT03141866.

## Strengths and limitations

- Mixed methods feasibility study employing both quantitative and qualitative research methodologies
- Specially adapted exercise interventions for frail geriatric populations
- Difficult to reach (and often excluded) participant population
- Single site study

For peer review only

- **Key words:** Elderly; exercise; feasibility; frail; geriatric; hospital; inpatient; intervention; older adults; patient; physical activity.

## Background

Frailty is a common and clinically significant condition within geriatric populations (1), predominantly due to its association with adverse health outcomes such as hospitalization, disability and mortality (1-6). Although the exact prevalence of frailty within this population is poorly defined due to the lack of a single standardised operational definition for the classification of frailty, it is generally believed that the prevalence of frailty amongst community-dwelling older adults ranges between 7.0 – 16.3% (1,7). Presently there are no well-evidenced, pooled estimates of the overall prevalence of frailty within geriatric hospital inpatients. However, there are several studies which have primarily aimed to produce estimates of the prevalence of frailty within this population (8-15). Although there is an evident need for more robust research to thoroughly assess the overall prevalence of frailty within this setting, through analysis of these existing studies, dependent upon the criteria utilised, the prevalence of frailty in geriatric hospital inpatients ranges from approximately 27 – 94%. In the five of the eight studies which utilised the Fried Frailty phenotype (2) as the operational definition of frailty, there is a narrower range (27–48.5%), with a mean prevalence of frailty across the five studies of 37.5±6.76% (12,16-19).

Although there is no one standardised and universally utilised operational definition of frailty, one of the most commonly utilised is the Fried frailty phenotype (2). This proposes that frailty be defined as a clinical syndrome in which three or more of the five following criteria are present: unintentional weight loss (≥ 10lbs in the past year), self- reported exhaustion, weakness (grip strength), slow walking speed and low levels of physical activity.

For peer review only - http://bmjopen.bmj.com/site/about/guidelines.xhtml

Exercise interventions have been proposed as potentially offering the best form of treatment for frail older adults (20); with exercise shown to be a significantly beneficial treatment for this population with regard to multiple components of health, and even shown to mediate the reversal of frailty in some cases (21-24). However, while there is evidence of the benefits of exercise relating to the prevention, treatment, and reversal of frailty, it is universally noted that there needs to be more high quality studies within this area to truly assess the impact of exercise in frail geriatric populations within different settings, particularly relating to its effects on broader aspects of health and well-being (1).

This present study will assess the feasibility and efficacy of short duration (2-week), intensive (five-days per week), specially adapted exercise interventions within a delayed transfer of care hospital ward setting. Feasibility will relate to the eight main areas of focus for feasibility studies (25), while efficacy will be assessed through limited efficacy testing of the impact of the interventions on the secondary dependent variables relating to multi-dimensional health and functional capacity.

Such research is very timely and pertinent, as current demographic trends indicate that by the year 2030 almost one in six of the European population will be aged 60 or over, and the number of older people will grow to 247 million by 2050, representing a 35% increase from 2017, with one in four older adults being over 85 by 2040 (26). This coupled with continual progressive declines in the rate of physical activity, at all stages of the lifespan (27), leaves the population particularly susceptible to the development of disease and co-morbidities associated with a lack of physical activity and an increase in sedentary behaviour (28). Moreover acute hospital admission for older adults are associated with further loss of physical activity and represent a period of increased susceptibility to sarcopenia and frailty (29). Frailty is associated with longer stay and increased rates of mortality in hospitalised older adults, as well as serving as a predictor of readmission (12, 30). Therefore, there is an urgent need to examine the effect of such interventions within this setting, and whether these interventions can be employed to improve various aspects of health in frail

For peer review only - http://bmjopen.bmj.com/site/about/guidelines.xhtml

older populations in inpatient hospital ward settings, as well as their efficacy in specifically treating, preventing and reversing frailty. Preliminary research has shown some success in the implementation of exercise interventions to reverse functional decline in general geriatric inpatient populations (31), however, to the authors' knowledge this present study is the first to attempt such an intervention in frail delayed transfer of care patients.

# Methods and analysis

## Aims and objectives

The primary aim of this study is to assess the feasibility of a proposed future trial in this setting, which aims to assess the impact of specially adapted exercise interventions on the physiological, psychological, cognitive, social, and emotional health, and functional capacity of frail geriatric populations within a hospital ward setting; recognising health as a multi-factorial concept incorporating multiple inter-related dimensions. The secondary aim of this feasibility study is to assess the efficacy of the interventions on the primary dependent variables of the proposed future clinical trial within this setting.

The primary and secondary aims of this study will be achieved through the sequential achievement of the following objectives: 1) Recruitment of eligible participants from the Harborne Ward of the Queen Elizabeth Hospital Birmingham, Mindelsohn Way, Birmingham, United Kingdom. 2) Baseline assessment of the secondary dependent variables related to multi-dimensional health. 3) Assessment of the feasibility of the study as it relates to the eight-primary areas of focus for feasibility studies (acceptability, demand, implementation, practicality, adaptation, integration expansion and limited-efficacy testing) (25). 4) Post-intervention assessment of all primary and secondary dependent variables.

The research questions of this study relate to the eight aforementioned areas of focus of this feasibility study, incorporating the following questions relating to the feasibility and efficacy of the study within this setting: Can it work? Will it work? Does it work? (25) (Table 1).

For peer review only

## Design overview

This feasibility study will utilise a 2-week, interventional, independent measures research design (Figure 1).

*(Insert Figure 1)*

A variation of a stepped-wedged design / rolling recruitment will be utilised, with the interventions being conducted multiple times over the course of several months in order to maximise the potential sample size due to constraints of the setting: a delayed transfer of care hospital ward for patients prior to official discharge, with the majority of patients residing on the ward for > 3 weeks, and approximately 25 patients on the ward at any given time.

The independent variables of the study will comprise of two exercise training interventions: a specially adapted machine-based resistance training intervention, and Move It Or Lose It (MIOLI), an established community-based exercise intervention for older adults. A control group will be utilised within the proposed future clinical trial but will not be utilised within this feasibility study as one of the primary purposes of this study is to assess the feasibility, and to a limited degree the efficaciousness, of the interventions within this setting. (Figure 2). *(Insert Figure 2)*

In order to ensure this present study is as scientifically valid as possible a number of precautions have been taken to protect the internal and external validity of the study within its methodological design:

First, for each participant, all testing procedures (baseline and post-intervention (2-weeks) will be conducted at approximately the same time of day (+/- two hours). This will be controlled in order to protect the findings of the study from changes in the dependent variables which may be attributable to circadian variation rather than manipulation of the independent variables (32). The hypothesis of the study will not be divulged to participants prior to or during the conduction of the study in order to control for any potential degree of demand characteristics; a scenario where participants alter their behaviour and/or answers, in order to align with what they believe is potentially the 'desired' outcome of the study. All dependent variable

For peer review only - http://bmjopen.bmj.com/site/about/guidelines.xhtml

testing sessions will take place at least 24 hours after the cessation of the previous training session for each participant. This will be implemented to ensure acute fatigue does not become a contributing factor to the results of the study, specifically relating to the secondary dependent variables, but also the feasibility of such practice during a proposed future clinical trial. The order in which dependent variables are tested will be counter-balanced throughout the study at each assessment timepoint in order to attempt to protect the study from practice effects, especially order effects, where a participant has been exposed to a specific order of testing before and as such performs better on subsequent testing procedures of the same material. Additionally, only one intervention will be run at a given time, protecting against potential subconscious selection bias amongst the research team relating to group allocation of participants. Finally, in order to increase the external validity of the study, eligibility criteria will be kept as minimalistic as possible (within the limits of safety and reason), in order to allow as inclusive a proportion of this population as possible, and in such producing findings which are applicable to not only those within the study, but to the greater population of frail geriatric hospital inpatients and particularly those within delayed transfer of care settings.

## Eligibility

This study is open to both men and woman whom meet the following eligibility criteria: inpatient on the Harborne ward of the Queen Elizabeth hospital Birmingham, Mindelsohn Way, Birmingham, United Kingdom; ≥ 65 years of age; frail according to the Fried Frailty Phenotype criteria (2); ability to speak and read in English; not currently taking part in any other clinical trial which could potentially impact upon or influence the findings of this study; not currently terminally ill with life expectancy less than the duration of the study; no severe sensory impairment which would profoundly impact upon ability to undergo the intervention, even once appropriate adaptations have been made; anticipated by their care team to remain on the ward for approximately 21 days post enrolment into the study.

## Interventions

For peer review only

All participants will undergo 10 sessions in total throughout the two-week intervention in either one of the two interventions. All session will be conducted individually under the guidance of a qualified trainer. A maximum of one session will be performed each day, and sessions will not be performed on any more than a maximum of three consecutive days throughout the duration of the study (Table 2).

## Exercise Intervention 1: Specially-adapted resistance training intervention

This intervention will be comprised of an intensive (five days per week), short duration (2 week), approximately 35 minutes per session, machine-based resistance training intervention. The exercises performed will specifically target the lower limbs through a combination of strength and power training utilising a leg press and leg extension machine (Figure 3). *(Insert Figure 3)*

An outline of the protocol for each session can be found in Figure 4. *(Insert Figure 4)*

## Exercise Intervention 2: Move It Or Lose It (MIOLI)

This intervention will comprise of an intensive (five days per week), short duration (2 week), approximately 35 minutes per session, chair-based exercise intervention. The exercises conducted will relate to strength, power, flexibility and aerobic capacity, predominantly targeting the lower limbs, but also incorporating the upper body and core. An outline of the protocol for each session can be found in Figure 5. *(Insert Figure 5)*

## Dependent Variables

### Primary dependent variables

The primary dependent variables will relate to the eight primary areas of focus of feasibility studies (25) (utilised to establish the feasibility of a proposed future clinical trial within this setting), relating to:

For peer review only

acceptability, demand, implementation, practicality, adaptation, integration, expansion and limited-efficacy testing.

These dependent variables will be assessed through semi-structured interviews with study participants and focus groups with ward staff post-intervention. Participant uptake and adherence records will also be employed throughout. These methods seek to attain answers to the following questions and parameters relating to the eight primary areas of enquiry for this feasibility study outlined in further detail in Table 1.

*(Insert Table 1 - Landscape)*

In order to enhance trustworthiness in the qualitative component of this research, several methods will be employed**:**

The researcher gathering the data will keep a reflective journal in which they will record information about themselves, their activities and the methods used. Field notes will include time, date and location, participant's actual notes, and the researcher's own questions and comments. This will lend to logging and documenting what is learned about the study, the intervention, the setting, the participants, and used to refine focus for future interviews through assessing the following questions: What is important? What is it I need to find out more about? What would I want to focus on more closely if I could do the interview again, or in future interviews? (33).

Data will be gathered from study participants and ward staff in order to collect data from multiple sources (triangulate information). This study will also employ more than one researcher to analyse the qualitative data in order to enhance triangulation and validity.

Enough details will be given about the participants and the setting to make decisions about the quality of the findings from the qualitative analysis. Detailed descriptions about the participants experiences and the setting will be provided by the researcher.

In the qualitative data analysis, clarification of all possible researcher biases will be made known. For example, it will be articulated that the researcher is an advocate of physical activity as a means to promote

For peer review only

health, prescribing to the theoretical and practical concept of exercise as medicine, and hence there may be some form of unconscious subjective bias in this context. However, it should also be noted that the researcher within this study is also an advocate of science to an equal or even greater extent, and as such any such bias in subjective analysis would potentially be counteracted in this sense.

Interviews - "a conversation with a purpose" (34) will be the primary method of data gathering utilised, as it enables large amounts of information to be gathered relatively quickly. Specifically, this study will employ semi-structured interviews, with open questions in a conversational format. There will be several pre-determined themes, topics and questions to be discussed, specifically relating to the eight areas of focus of the primary dependent variables of this study. All interviews and focus groups will be audio-recorded in order to facilitate future transcription. The qualitative element of this study will also explore opportunities for Patient and Public Involvement (PPI) in the research design of the proposed future clinical trial.

As this study will utilise a mixed methods research approach, employing both qualitative and quantitative research methodologies, this will facilitate the potential for elaboration and expansion of these findings of individual methodologies through complementary analysis. The qualitative aspect of this feasibility study, aimed at assessing the primary dependent variables, will predominantly take a phenomenological approach to understand the experiences of individuals involved in the study (35).

## Secondary Dependent Variables

The secondary dependent variables relating to multi-dimensional health (comprising the proposed primary dependent variables of the future clinical trial) will be:

Physiological*: Serum cortisol, dehydroepiandrosterone-sulphate (DHEAS), cortisol: DHEAS ratio, C-reactive protein (CRP), Interleukin 6 (IL-6), Tumor Necrosis Factor alpha (TNFα), Interferon gamma (IFNy)

For peer review only

Functional: Hand grip strength (Southampton protocol (36), Leg strength and power output (37,38), Short Physical Performance Battery (SPPB) (39), Katz Index of Independence in Activities of Daily Living (Katz ADL) (40), and the Fried Frailty Phenotype (2)

Psychological / Emotional: Geriatric Depression Scale (GDS) (41), Hospital Anxiety Depression Scale (HADS) (42)

Cognitive: Standardized Mini-Mental State Examination (SMMSE) (43)

Social: Interpersonal Support Evaluation List (ISEL-12) (44)

*All blood samples will be obtained through venepuncture. Serum will be analysed for the physiological dependent variables relating to cortisol, DHEAS and CRP (assessed by commercial ELISA kit). Inflammatory cytokines (IL-6, TNFα, IFNy) will be assayed using a multiplex commercial kit (R&D Systems). These specific physiological variables will be examined due to their association with the ageing process, and previous research which have both proposed and indicated that exercise is potentially capable of altering these variables (45-50).

## Data collection

Data will predominantly be collected at two main time points: baseline and post-intervention (Table 2).

*(Insert Table 2 - Landscape)*

**Baseline Assessment:** Participants' baseline socio-demographic and information for the secondary dependent variables will be collected between 5 – 2 days prior to intervention commencement. One repetition maximum (1RM) for the specially adapted resistance training equipment will also be assessed during this time period (after all baseline testing has been completed, and at least 24 hours after baseline testing which requires physical exertion, which may impact on the accuracy of the 1RM measurements).

For peer review only - http://bmjopen.bmj.com/site/about/guidelines.xhtml

1RM will be determined based on estimation from participants five repetition maximum (5RM) utilising the Epley formula: 1RM = w (1 + r/30), where 1 = weight, and r = repetitions (38). The protocol employed during obtainment of participants 5RM has been adapted from (37) (Supplementary file 1). The resistance bands which participants in the MIOLI intervention will use will similarly be assessed during this period.

**Post-intervention assessment:** Primary and secondary dependent variables will be assessed between 1 – 5 days post intervention cessation. All assessments will be conducted at least 24 hours post the cessation of the last training session.

Adherence rates in the intervention will be recorded as the number of repetitions completed in a set (100% required for adherence to that exercise), and the number of exercises for which there was 100% adherence. If participants meet these parameters for each exercise session, they will be considered to be in 100% adherence to the intervention. For example, if a participant adheres to 100% of the intervention for 9 sessions, but only 90% for 1 session, then they will have a 99% adherence rate. Adherence rates, whether high, or low, may signify that the interventions may have been too demanding, too easy or optimal. Information will also be collected throughout the study related to uptake and retention rates.

## Data monitoring

Data will be monitored by the trial management committee at monthly intervals. Prior to analysis data entry checking will be conducted for accuracy on 10% of all participants, and queries resolved through discussion with the trial management committee and access to the source documents. Data management will adhere to the PANINI data management plan, which was developed in accordance with national and European principles as part of the university research governance and European Commission research governance 0guidelines. Thus, data management for this project adheres to the FAIR principles (51).

## Sample size

For peer review only

This study aims to recruit a convenience sample of n = ~ 30 participants: 15 in each intervention. No formal power calculations were conducted due to the feasibility nature of this study. This sample size was based on initial expectations related to the obtainment of data saturation in the qualitative component of the study given its phenomenological approach (52,53).

## Identification, Consent and Recruitment

**Identification:** Patients on the ward at the commencement of the study will initially be screened by their care team for the known presence of any severe sensory impairments which would exclude them from participation. Due to the study's rolling recruitment, this initial screening will continue throughout the study as additional patients are admitted to the ward. Participant information sheets will not be distributed to patients who are deemed to be medically unfit or due for imminent discharge as identified by their care team. All other aspects of the screening process will occur after consent has been obtained. After identification, potential participants will be approached by the researcher with an information sheet related to the study and asked if they would be interested in participating, or if they would like to receive more information about the study (Supplementary file 2). The information sheet will contain all the most pertinent information relating to the study and specifically what it would require from potential participants. Potential participants will be given ≥ 24 hours to consider whether or not they would like to participate.

**Consent:** At this stage potential participants will also be provided with an informed consent form and asked if they would be interested in participating (Supplementary file 3). If it is deemed that a potential participant lacks the capacity to consent, a personal consultee will be sought. A personal consultee will be someone who cares for the patient, or is interested in their welfare (but not a paid professional), and who is prepared to be consulted and give advice on what they believe the patient's wishes would be, were they to have the capacity to consent for themselves. If a personal consultee cannot be found, a nominated consultee will be sought. A nominated consultee will be someone who is familiar with the patient in a professional context and can adequately advise on whether they believe participation would be in the patients' best interest.

For peer review only - http://bmjopen.bmj.com/site/about/guidelines.xhtml

These processes will be informed by the UK Department of Health's guidance on nominating a consultee for research involving adults who lack the capacity to consent (54); in accordance with section 32(3) of the Mental Capacity Act 2005 (55). All efforts possible will be made in this regard to include participants whom lack the capacity to consent, as intrinsically within the research team from a personal and professional perspective we would consider it unethical to exclude potential participants from participating in a study, which can potentially benefit them and their overall health status, due to the fact that they lack the capacity to consent.

**Recruitment:** Following consent being obtained from the participant themselves, or the obtainment of a declaration from a consultee, all consented participants will be screened for the remaining eligibility criteria.

For peer review only

## Statistical methods

### Primary Dependent Variables

Analysis of the primary dependent variables will be based on an inductive process utilising Interpretative Phenomenological Analysis (thematic analysis). Two researchers will be employed to analyse the data to increase triangulation from the analysis perspective, having already triangulated data acquisition through obtainment from multiple sources (study participants and ward staff). All semi-structured interviews and focus groups will be audio-recorded. Data synthesis will be performed through verbatim transcription. The three main steps of Interpretative Phenomenological Analysis will be followed (35): 1) The generation of themes from transcripts within the areas of feasibility inquiry. As an iterative process, these themes will be continuously reviewed and adapted based on the emergence of information in subsequent transcripts 2) The collation and separation of these themes within each of the areas of feasibility inquiry 3) Written interpretation of the resultant themes within each of the areas of feasibility and their relationship to one another. At all stages within this process, reflective journal entries and field notes will be utilised to provide a more comprehensive understanding of the findings, in addition to incorporating additional feasibility

information related to uptake and retention rates, and limited efficacy testing of the secondary dependent variables in the final analysis to provide a comprehensive assessment of the feasibility of the study.

**Secondary Dependent Variables**

Statistical analysis of the secondary dependent variables will be performed using IBM Statistical Package for Social Sciences (SPSS) software. These analyses will be performed as part of the limited efficacy testing regarding the potential impact of the interventions on the secondary dependent variables (proposed primary dependent variables of the future clinical trial). These analyse will provide an estimation of efficacy and provide valuable insight regarding feasibility; useful to informing the design of the future powered clinical trial. Specifically, the following statistical analysis which will be utilised: 2x2 way independent measures ANOVA's (analysis' of variance consisting of two independent variables; the specially adapted resistance training intervention, and the Move It Or Lose It (MIOLI) intervention, each with two levels: baseline and post-intervention. A subsequent post-hoc test will be utilised if a significant main effect or interactions are found. Pearson product correlations will also be utilised between various socio-demographic variables (such as age and sex) and the dependent variables of this study, to assess possible relationships between differences in socio-demographic factors and changes in the dependent variables.

Central tendency and variability measurements consisting of parameters such as the mean, median and mode, and standard deviation and range of scores respectively, will also be utilised during the analysis of data for illustrative purpose. Significance levels will be set at 0.05 ($p \leq 0.05$), and effect sizes will be reported for all analyses. In order to establish if the assumptions of parametric statistics have been met regarding the assumption that there is a normal distribution of data, the data will be analysed for skewness and kurtosis. In the event data does not fulfill the assumptions of parametric statistics, the non-parametric equivalent of the aforementioned statistical analysis will be employed, namely the Scheirer-Ray-Hare test, and Spearman's rank-order correlations. As the quantitative component of this study has not been powered given the feasibility nature of the study, the examination of the efficacy of the intervention to impact these

For peer review only

secondary dependent variables is limited; and interpretation of these results should be treated with caution pending the future powered clinical trial. All results will be reported with effect size and 95% confidence intervals.

## Data storage and protection

Participants' identity or other personal information will be kept confidential. Participants will be assigned a unique ID number under which all study information will be stored in a secure file on an encrypted and password protected computer and laptop at the University of Birmingham (UoB). Physical data (e.g. Case Report Forms (CRF's)) will be identifiable only by ID number and stored in a locked filing cabinet at the School of Sport, Exercise and Rehabilitation Sciences at UoB, accessible only by the research team. Participants' personal data (name, date of birth) and consent forms matching them to their ID number, will be stored securely in a locked filing cabinet, separate from all other data and/or in a password protected master sheet on an encrypted and password protected computer and laptop at UoB. All serum samples will be stored in Human Tissue Act (HTA) compliant facilities at UoB for three years then destroyed.

All hard copy data collected on CRF's will be stored in a linked-anonymised format securely for 10 years then destroyed. All personal data (consent forms, master sheet linking participant IDs to names and contact details) will be stored for 10 years then destroyed. All computerised data will be archived on UoB servers in anonymised form for 10 years in the first instance in accordance with the UoB Code of Practice for Research, and the Data Protection Act (2018).

Following analysis for this specific study, all data will be anonymised and entered into a European 'PANINI' open access database that this project is part of and may be analysed in future ethically approved research across the PANINI network. The PANINI shared dataset will be made open access at the conclusion of the funding for the PANINI network including this study in 2020 and stored for at least 10 years as an open access searchable published dataset.

For peer review only

## Patient and Public involvement

All authors are strong proponents of patient and public involvement and engagement with research and believe the findings of the study will be important to aid the facilitation of improvements in the care of frail older hospital inpatients. Given the feasibility nature of this research, the qualitative element of this study will explore opportunities for Patient and Public Involvement (PPI) in the research design of the future clinical trial. The findings of this study will be disseminated to participants upon request, and our patient and public involvement groups.

## Ethics and dissemination

### Ethical approval and consent to participate

This study has been granted a favourable ethical opinion by the Coventry and Warwickshire NHS Research Ethics Committee (REC) (17/WM/0390) on the 12/03/2018. This study has also been sponsored by the University of Birmingham, after review by the sponsor's research governance office (sponsor registration number: RG_17-108).

### Dissemination

The findings of this study will be disseminated through publication of scientific papers in open access scientific journals, public engagement events, online via social media (Twitter, Instagram) and the PANINI project website (56,57), presentation at various conferences, and to study participants upon request.

### Safety reporting and monitoring

For peer review only

Adverse events (AE) and serious adverse events (SAE) will be monitored and if applicable recorded, by the primary researcher at each testing or exercise session and reported weekly to the principle investigator (PI) and reviewed in the case of AEs. SAEs will be reported immediately to the PI who will complete an SAE form indicating causality and severity. The chief investigator (CI) will then submit this to the sponsor's research governance office, the Research Ethics Committee (REC) and University Hospitals Birmingham (UHB) research governance office, within 24 hours. UHB local policies and Standard Operating Procedures (SOP's) for all safety reporting will also be followed by the research team. SAEs related to pre-existing conditions will not be reported. Actions following an AE or SAE would be as standard, i.e. direct referral to their clinical care team, who then may wish to treat the AE or SAE themselves or where appropriate refer the participant to another relevant medical professional, and to recommend that the participant withdraw from the study unless they have been cleared to continue exercise by their attending physician.

## Trial registration

This study has been registered on ClinicalTrials.gov under the identifier number: NCT03141866.

## Trial status

This trial commenced on the 03/09/2018 on the Harborne ward of the Queen Elizabeth Hospital Birmingham, with anticipated completion of data collection scheduled for 09/08/2019.

## Funding

This review has been funded by the European Commission's Horizon 2020 research and innovation programme under the Marie Sklodowska-Curie grant agreement (675003); of which PD is a Marie Sklodowska-Curie Doctoral Research Fellow, AW, JL and CAG doctoral supervisors, and AW the grants Principal Investigator.

For peer review only

## Author contributions

PD designed the study protocol, and associated manuscript for publication, with supervision, input and feedback from AW at all stage of the design and writing process. JL and CAG reviewed and revised the manuscript prior to publication. All authors have read and approved the final manuscript.

## Competing interests

The authors have no competing interests to declare.

For peer review only

## References

(1) Rodriguez-Mañas L, Fried LP. Frailty in the clinical scenario. The Lancet 2015 /02/14;385(9968):e9.

(2) Fried LP, Tangen CM, Walston J, Newman AB, Hirsch C, Gottdiener J, et al. Frailty in older adults evidence for a phenotype. The Journals of Gerontology Series A: Biological Sciences and Medical Sciences 2001;56(3):M157.

(3) Gill TM, Gahbauer EA, Allore HG, Han L. Transitions between frailty states among community-living older persons. Arch Intern Med 2006;166(4):418-423.

(4) Clegg A, Young J, Iliffe S, Rikkert MO, Rockwood K. Frailty in elderly people. The Lancet 2013 March 8,;381(9868):752-762.

(5) Sternberg SA, Schwartz AW, Karunananthan S, Bergman H, Mark Clarfield A. The identification of frailty: a systematic literature review. J Am Geriatr Soc 2011;59(11):2129-2138.

(6) Sourial N, Bergman H, Karunananthan S, Wolfson C, Payette H, Gutierrez-Robledo LM, et al. Implementing Frailty Into Clinical Practice: A Cautionary Tale. J Gerontol A Biol Sci Med Sci 2013 -05-02:glt053.

(7) Collard RM, Boter H, Schoevers RA, Oude Voshaar RC. Prevalence of frailty in community-dwelling older persons: a systematic review. J Am Geriatr Soc 2012 Aug;60(8):1487-1492.

(8) Joosten E, Demuynck M, Detroyer E, Milisen K. Prevalence of frailty and its ability to predict in hospital delirium, falls, and 6-month mortality in hospitalized older patients; 24393272. BMC Geriatrics 2014;14(1).

(9) Oliveira DR, Bettinelli LA, Pasqualotti A, Corso D, Brock F, Erdmann AL, et al. Prevalence of frailty syndrome in old people in a hospital institution. Revista Latino-Americana de Enfermagem 2013 08/;21(4):891-898.

(10) Ekerstad N, Swahn E, Janzon M, Alfredsson J, Löfmark R, Lindenberger M, et al. Frailty is independently associated with short-term outcomes for elderly patients with non-ST-segment elevation myocardial infarction. Circulation 2011 Nov 29,;124(22):2397-2404.

(11) Purser JL, Kuchibhatla MN, Fillenbaum GG, Harding T, Peterson ED, Alexander KP. Identifying Frailty in Hospitalized Older Adults with Significant Coronary Artery Disease. Journal of the American Geriatrics Society 2006 November 1,;54(11):1674-1681.

(12) Khandelwal D, Goel A, Kumar U, Gulati V, Narang R, Dey AB. Frailty is associated with longer hospital stay and increased mortality in hospitalized older patients. Journal of Nutrition, Health and Aging 2012;16(8):732-735.

(13) Maguet PL, Roquilly A, Lasocki S, Asehnoune K, Carise E, Martin MS, et al. Prevalence and impact of frailty on mortality in elderly ICU patients: a prospective, multicenter, observational study. Intensive Care Med 2014 /05/01;40(5):674-682.

(14) Hewitt J, Moug SJ, Middleton M, Chakrabarti M, Stechman MJ, McCarthy K. Prevalence of frailty and its association with mortality in general surgery. Am J Surg 2015 Feb;209(2):254-259.

(15) Andela RM, Dijkstra A, Slaets JP, Sanderman R. Prevalence of frailty on clinical wards: Description and implications. International Journal of Nursing Practice 2010 February 1,;16(1):14-19.

(16) Joosten E, Demuynck M, Detroyer E, Milisen K. Prevalence of frailty and its ability to predict in hospital delirium, falls, and 6-month mortality in hospitalized older patients. BMC Geriatrics 2014;14(1).

(17) Oliveira DR, Bettinelli LA, Pasqualotti A, Corso D, Brock F, Erdmann AL. Prevalence of frailty syndrome in old people in a hospital institution. Rev Lat Am 2013;21(4):891-898.

(18) Purser JL, Kuchibhatla MN, Fillenbaum GG, Harding T, Peterson ED, Alexander KP. Identifying frailty in hospitalized older adults with significant coronary artery disease. J Am Geriatr Soc 2006;54(11):1674-1681.

For peer review only

(19) Le Maguet P, Roquilly A, Lasocki S, Asehnoune K, Carise E, Saint Martin M, et al. Prevalence and impact of frailty on mortality in elderly ICU patients: A prospective, multicenter, observational study. Intensive Care Med 2014;40(5):674-682.

(20) Theou O, Stathokostas L, Roland KP, Jakobi JM, Patterson C, Vandervoort AA, et al. The effectiveness of exercise interventions for the management of frailty: a systematic review. Journal of aging research 2011;2011.

(21) Arrieta H, Rezola-Pardo C, Gil SM, Virgala J, Iturburu M, Antón I, et al. Effects of Multicomponent Exercise on Frailty in Long-Term Nursing Homes: A Randomized Controlled Trial. J Am Geriatr Soc 2019.

(22) Tarazona-Santabalbina FJ, Gmez-Cabrera MC, Prez-Ros P, Martnez-Arnau FM, Cabo H, Tsaparas K, et al. A multicomponent exercise intervention that reverses frailty and improves cognition, emotion, and social networking in the community-dwelling frail elderly: a randomized clinical trial. Journal of the American Medical Directors Association 2016;17(5):426-433.

(23) Ng TP, Feng L, Nyunt MSZ, Feng L, Niti M, Tan BY, et al. Nutritional, physical, cognitive, and combination interventions and frailty reversal among older adults: a randomized controlled trial. Am J Med 2015;128(11):1236. e1.

(24) Fiatarone MA, Marks EC, Ryan ND, Meredith CN, Lipsitz LA, Evans WJ. High-Intensity Strength Training in Nonagenarians: Effects on Skeletal Muscle. ResearchGate 1990 /07/01;263(22):3029-34.

(25) Bowen DJ, Kreuter M, Spring B, Cofta-Woerpel L, Linnan L, Weiner D, et al. How we design feasibility studies. Am J Prev Med 2009;36(5):452-457.

(26) United Nations, Department of Economic and Social Affairs, Population Division. World Population Ageing 2017 (ST/ESA/SER.A/408). URL: https://www.un.org/en/development/desa/population/publications/pdf/ageing/WPA2017_Report.pdf. Accessed: 17th April 2019.

(27) Caspersen CJ, Pereira MA, Curran KM. Changes in physical activity patterns in the United States, by sex and cross-sectional age. Med Sci Sports Exerc 2000;32(9):1601-1609.

(28) Ford ES, Caspersen CJ. Sedentary behaviour and cardiovascular disease: a review of prospective studies. Int J Epidemiol 2012:dys078.

(29) Acute inflammation is associated with lower muscle strength, muscle mass and dependency in activities of daily living in male hospitalised older patients. AUSTRALASIAN JOURNAL ON AGEING: WILEY 111 RIVER ST, HOBOKEN 07030-5774, NJ USA; 2018.

For peer review only

(30) Hao Q, Zhou L, Dong B, Yang M, Dong B, Weil Y. The role of frailty in predicting mortality and readmission in older adults in acute care wards: a prospective study. Scientific reports 2019;9(1):1207.

(31) Martínez-Velilla N, Casas-Herrero A, Zambom-Ferraresi F, de Asteasu, Mikel L Sáez, Lucia A, Galbete A, et al. Effect of exercise intervention on functional decline in very elderly patients during acute hospitalization: a randomized clinical trial. JAMA internal medicine 2019;179(1):28-36.

(32) Atkinson G, Reilly T. Circadian variation in sports performance. Sports medicine 1996;21(4):292-312.

(33) Ortlipp M. Keeping and using reflective journals in the qualitative research process. The qualitative report 2008;13(4):695-705.

(34) Kahn RL, Cannell CF. The dynamics of interviewing; theory, technique, and cases. 1957.

(35) Smith JA, Osborn M. Interpretative phenomenological analysis. Doing social psychology research 2004:229-254.

(36) Roberts HC, Denison HJ, Martin HJ, Patel HP, Syddall H, Cooper C, et al. A review of the measurement of grip strength in clinical and epidemiological studies: towards a standardised approach. Age Ageing 2011 07/01/;40(4):423-429.

(37) Haff GG, Triplett NT. Essentials of strength training and conditioning 4th edition. : Human kinetics; 2015.

(38) Epley B. Poundage chart. Boyd Epley Workout.Lincoln, NE: Body Enterprises, 2985 1985;86:p. 86.

(39) Guralnik JM, Simonsick EM, Ferrucci L, Glynn RJ, Berkman LF, Blazer DG, et al. A short physical performance battery assessing lower extremity function: association with self-reported disability and prediction of mortality and nursing home admission. J Gerontol 1994;49(2):M94.

(40) Katz S, Chinn AB, Cordrey L. Multidisciplinary studies of illness in aged persons II: a new classification of functional status in activities of daily living. J Chronic Dis 1959;9(1):55-62.

(41) Yesavage JA, Brink TL, Rose TL, Lum O, Huang V, Adey M, et al. Development and validation of a geriatric depression screening scale: a preliminary report. J Psychiatr Res 1983;17(1):37-49.

(42) Zigmond AS, Snaith RP. The hospital anxiety and depression scale. Acta Psychiatr Scand 1983;67(6):361-370.

For peer review only

Protocol_Manscript_V2.0_11/07/2019

(43) Molloy DW, Alemayehu E, Roberts R. Reliability of a standardized mini-mental state examination compared with the traditional mini-mental state examination. Am J Psychiatry 1991;148(1):102-105.

(44) Cohen S, Mermelstein R, Kamarck T, Hoberman HM. Measuring the functional components of social support. Social support: Theory, research and applications: Springer; 1985. p. 73-94.

(45) Heaney JL, Carroll D, Phillips AC. DHEA, DHEA-S and cortisol responses to acute exercise in older adults in relation to exercise training status and sex. Age 2013;35(2):395-405.

(46) Córdova C, Lopes-e-Silva J, Pires AS, Souza VC, Brito CJ, Moraes CF, et al. Long-Term Resistance Training Is Associated with Reduced Circulating Levels of IL-6, IFN-Gamma and TNF-Alpha in Elderly Women. Neuroimmunomodulation 2011 /02/09;18(3):165-170.

(47) Smart NA, Larsen AI, Le Maitre JP, Ferraz AS. Effect of exercise training on interleukin-6, tumour necrosis factor alpha and functional capacity in heart failure. Cardiology research and practice 2011;2011.

(48) Fischer CP. Interleukin-6 in acute exercise and training: what is the biological relevance. Exerc Immunol Rev 2006;12(6-33):41.

(49) Petersen AMW, Pedersen BK. The anti-inflammatory effect of exercise. J Appl Physiol 2005;98(4):1154-1162.

(50) Goldhammer E, Tanchilevitch A, Maor I, Beniamini Y, Rosenschein U, Sagiv M. Exercise training modulates cytokines activity in coronary heart disease patients. Int J Cardiol 2005;100(1):93-99.

(51) Wilkinson MD, Dumontier M, Aalbersberg IJ, Appleton G, Axton M, Baak A, et al. The FAIR Guiding Principles for scientific data management and stewardship. Scientific data 2016;3.

(52) Morse JM. Designing funded qualitative research. 1994.

(53) Creswell JW, Poth CN. Qualitative inquiry and research design: Choosing among five approaches. : Sage publications; 2017.

(54) United Kingdom. Department of Health. Guidance on nominating a consultee for research involving adults who lack capacity to consent (8953). URL: https://webarchive.nationalarchives.gov.uk/20130123193236/http://www.dh.gov.uk/en/PublicationsandStatistics/Publications/PublicationsPolicyAndGuidance/DH_083131. Accessed: 5th July 2019. 2008.

For peer review only

(55) United Kingdom Mental Capacity Act, 2005. URL: https://www.legislation.gov.uk/ukpga/2005/9/section/32. Accessed 5th July 2019.

(56) Whittaker AC, Delledonne M, Finni T, Garagnani P, Greig C, Kallen V, et al. Physical Activity and Nutrition INfluences In ageing (PANINI): consortium mission statement. Aging clinical and experimental research 2018:1-8.

(57) University of Birmingham. Physical Activity and Nutritional INfluences In ageing (PANINI)  Available at: http://www.birmingham.ac.uk/generic/panini/index.aspx. Accessed 6th February, 2019.

For peer review only

# Tables

*Table 1. The eight primary areas of focus, outlining the research questions and methods of assessment*

| Area of focus | Potential questions | Methods of Assessment |
|---|---|---|
| Acceptability | • Will the proposed population be interested in participating in the study?<br>• What will the uptake be?<br>• Will the program be judged as suitable by the delivers of the program in addition to the program participants?<br>• Participant's opinions on hypothetically being randomized into a control group during a proposed future clinical trial? *<br><br>*Participants within the feasibility study will not be recruited as participants within the proposed future clinical trial in order to protect the scientific validity of the proposed future clinical trial, as the participants within the feasibility will already have undergone the interventions, or similar interventions. However, participants within the feasibility study will likely not still be residents on the ward, as the average stay for participants on the ward is approximately 3.5 weeks. As such once the proposed future clinical trial has commenced, all participants within the feasibility trial will likely have left the ward well in advance. | • Participant uptake analysis (All participants approached and eligible for the study, all of those successfully recruited to the study)<br>• Semi-structured interviews with participants<br>• Focus groups with study support staff. |
| Demand | • Will the proposed population of hospital inpatients participate in the study?<br>• What will adherence rates be?<br>• Are the staff on the ward open to the idea of having exercise interventions potentially on the ward long term if it proves effective? | • Analysis of uptake rates<br>• Exercise intervention adherence rates<br>• Focus groups with study support staff/ward staff. |
| Implementation | • What are the possible logistical issues with the setting which will need to be addressed or accounted for prior to the clinical trial?<br>• Can the interventions be successfully carried out within this setting?<br>• Can a single or double bind be successfully implemented within this setting? | • Semi-structured interviews with study participants.<br>• More in-depth with focus groups with study support staff. |
| Practicality | • What are the practical implications of the study with relation to time commitment of the researchers, relating to both the implementation of the interventions, and the testing of participants for the dependent variables of the proposed future clinical trial?<br>• Is it viable to potentially conduct follow-up testing on participants in the proposed future clinical trial? | • Semi-structured interviews with study participants.<br>• Focus groups with support staff. |

For peer review only



For peer review only - http://bmjopen.bmj.com/site/about/guidelines.xhtml

| | | |
|---|---|---|
| | • Do any alterations need to be made to the proposed primary dependent variables of the future clinical trial?<br>• If the interventions are successful in influencing parameters of health and functional capacity, will it potentially be possible to assess if these improvements are sustained during a two week follow up in the proposed future clinical trial, if the same is found? | |
| Integration | • How will the ward staff appraise the study?<br>• Will the interventions be easily integrated into the existing culture, protocols and procedures within the ward seamlessly? | • Focus groups with ward/support staff. |
| Adaptation | • Will any further adaptations be required to the existing interventions to make them more feasible or appropriate within this setting? | • Semi structured interviews with participants. |
| Expansion | • Can the Move It or Lose It intervention (an established chair-based exercise programme for older adults) be successfully expanded to this setting?<br>• Can be specially adapted resistance training equipment be successfully expanded to this setting? | • Semi-structured interviews / Focus group with the ward/study support staff. |
| Limited-efficacy testing | • Is two weeks a sufficient duration to potentially provide significant benefit to patients?<br>• Can intensive (five-six days per week), short duration (two weeks) physical activity interventions improve markers of multi-dimensional health, in very frail individuals? | • Analysis of the secondary dependent variables within the study (primary dependant variables of the future clinical trial)<br>• Analysis of uptake and adherence rates<br>• Analysis of the level of satisfaction with the interventions through questionnaires with participants post intervention. |

601

602

603



For peer review only - http://bmjopen.bmj.com/site/about/guidelines.xhtml

*Table 2. Study timeline of all major events during each round of recruitment (SPIRIT Schedule).*

| Week | Seated Physical Activity in Ageing (SPAA) Study Timeline | | | | | | |
|---|---|---|---|---|---|---|---|
| | Monday | Tuesday | Wednesday | Thursday | Friday | Saturday | Sunday |
| Week 0 | Identification + participant information sheet distribution | 24-hour consideration period | Recruitment, eligibility screening, and pre-intervention assessments | Recruitment, eligibility screening, and pre-intervention assessments | Recruitment, eligibility screening, and pre-intervention assessments | Recruitment, eligibility screening, and pre-intervention assessments | - |
| Week 1 | Training | Rest | Training | Training | Rest | Training | Training |
| Week 2 | Training | Rest | Training | Training | Training | Rest | Training |
| Week 3 | Post-Intervention Assessment | Post-Intervention Assessment | Post-Intervention Assessment | Post-Intervention Assessment | Post-Intervention Assessment | Post-Intervention Assessment | - |



For peer review only - http://bmjopen.bmj.com/site/about/guidelines.xhtml

# Figures

Figure 1. Trial schema of participant flow throughout the duration of the study.

Figure 2. The independent variables of the proposed future clinical trial.

Figure 3. Minato resistance training equipment utilised within the specially adapted resistance training intervention.

Figure 4. Exercise intervention 1- Specially adapted resistance training intervention protocol.

Figure 5. Exercise intervention 2 – Move It Or Lose It (MIOLI) chair-based exercise intervention protocol.

All figures are attached to this manuscript submission as separate documents titled "Figure 1", "Figure 2", "Figure 3", "Figure 4" and "Figure 5" accordingly.

For peer review only

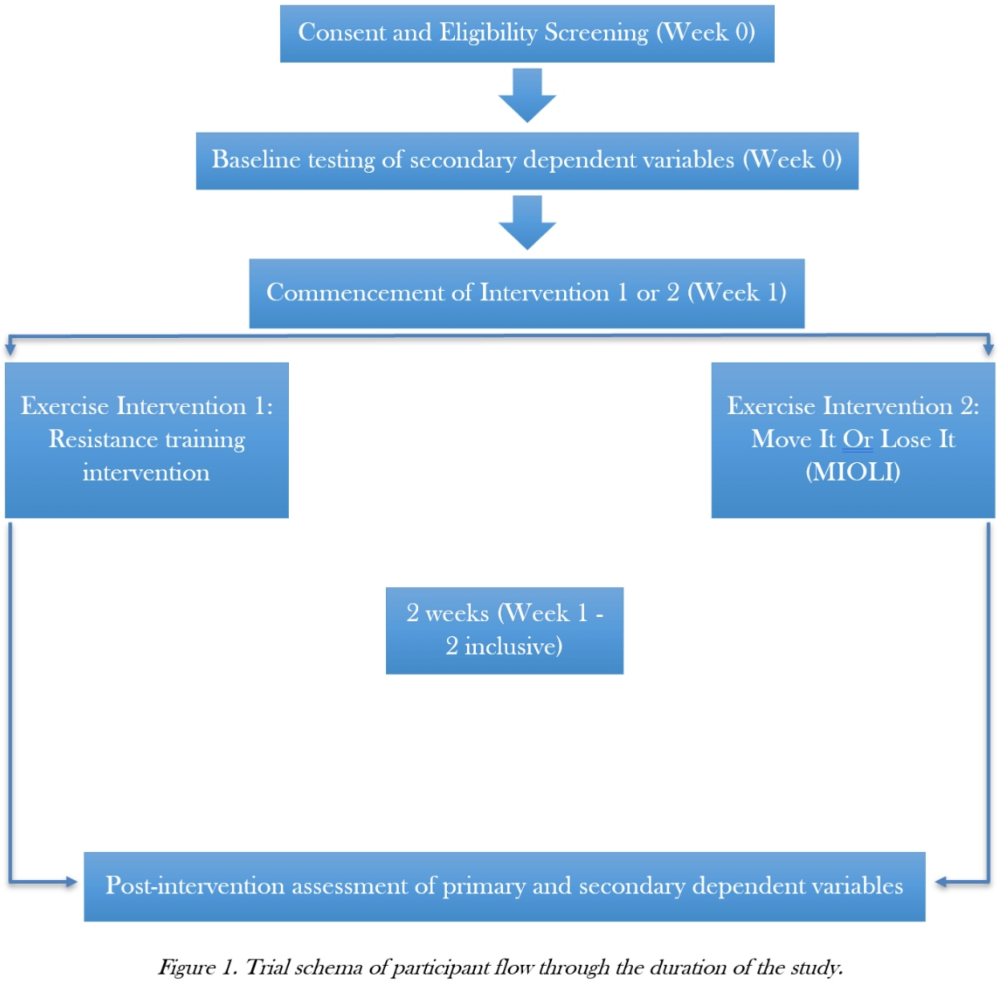


Figure 1. Trial schema of participant flow through the duration of the study.

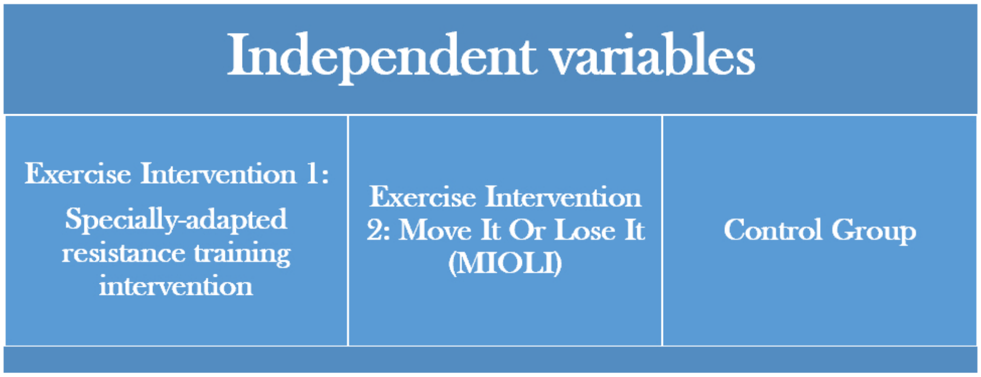


*Figure 2. The independent variables of the proposed future clinical trial.*

Figure 2. The independent variables of the proposed future clinical trial.

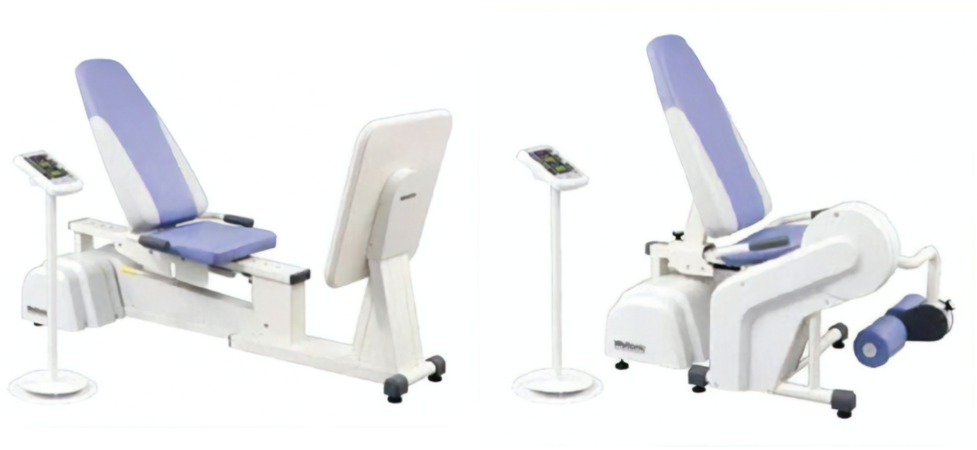

*Figure 3. Minato resistance training equipment utilised within the specially adapted resistance training intervention*

Figure 3. Minato resistance trianing equipment utilised within the speically adapted resistence training intervention.

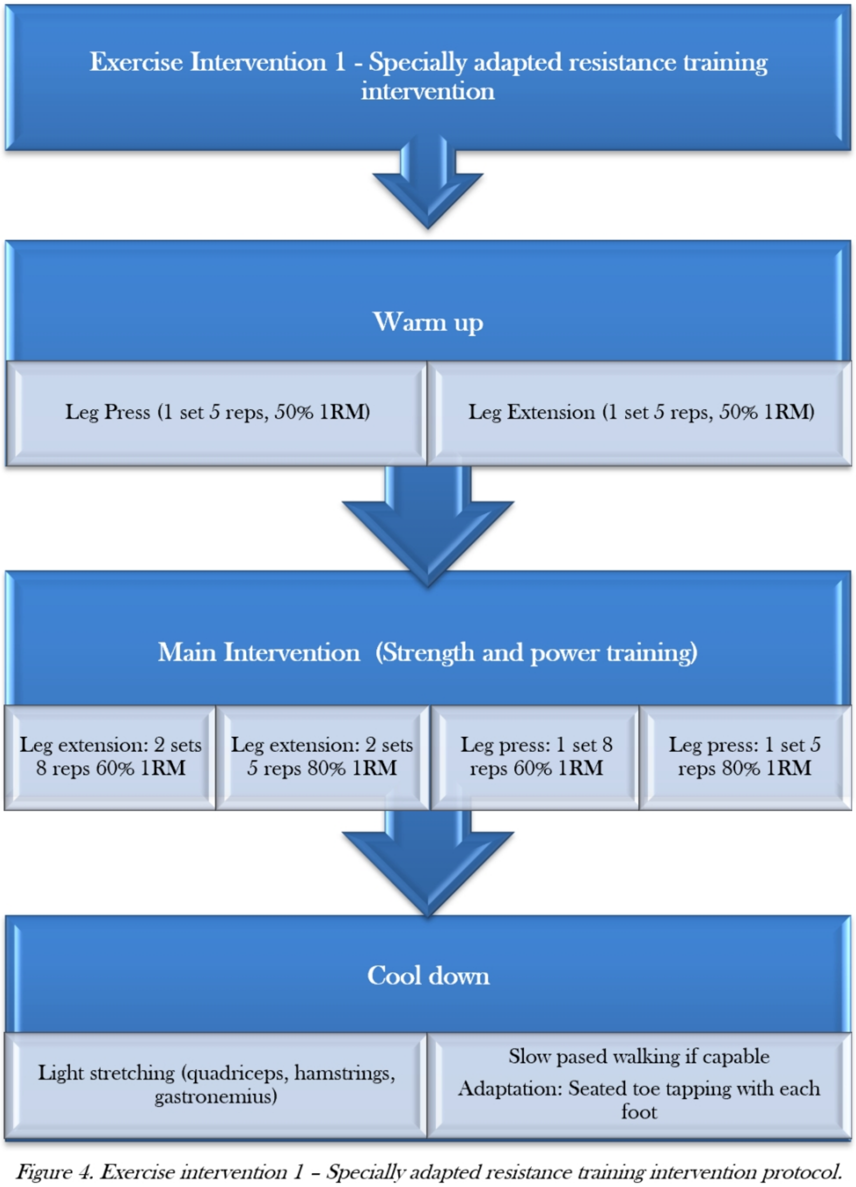


Figure 4. Exercise intervention 1 - Specially adapted resistance training intervention.

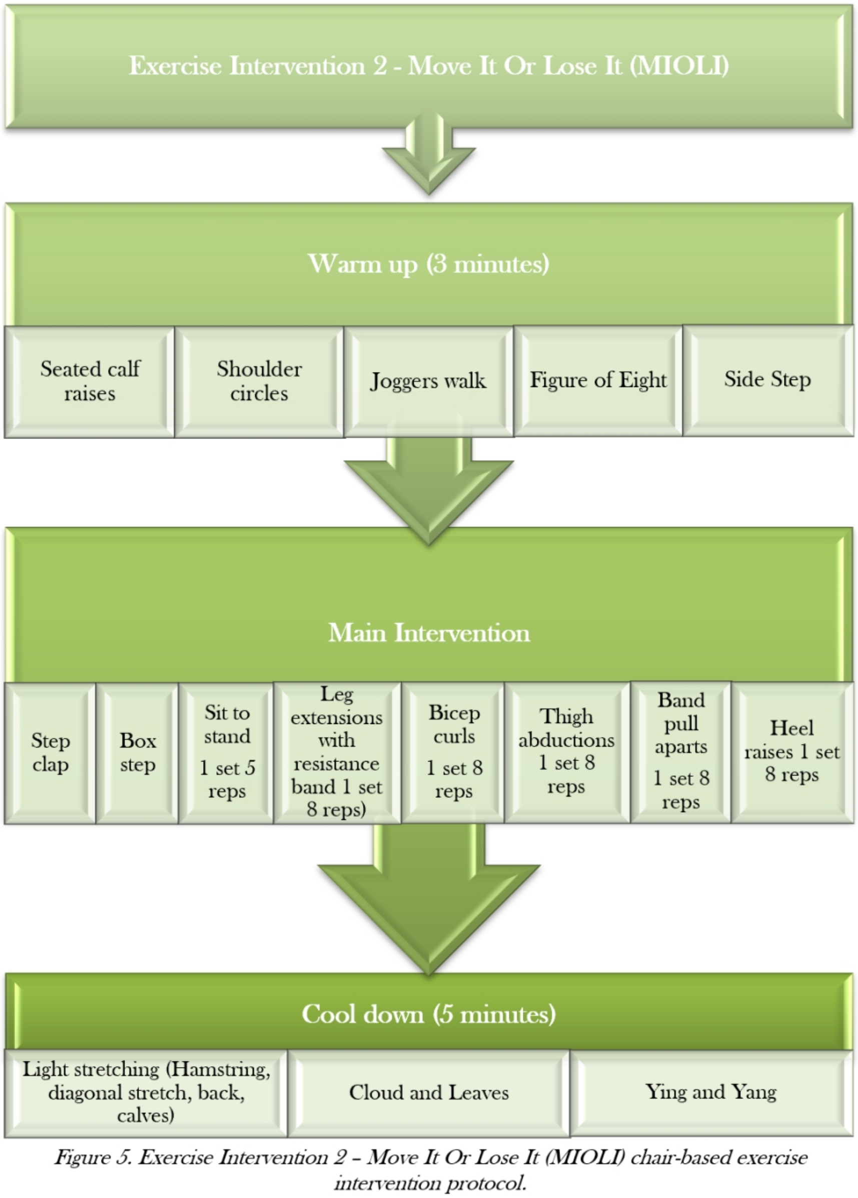


Figure 5. Exercise Intervention 2 - Move It Or Lose It (MIOLI) chair-based exercise intervention protocol.

# Leg Strength Assessment

Leg strength will be assessed by an estimated one repetition maximum (1RM) obtained from participants five repetition maximum (5RM) on the leg press and leg extension machines pre- and post-intervention. The protocol for this assessment can be found below:

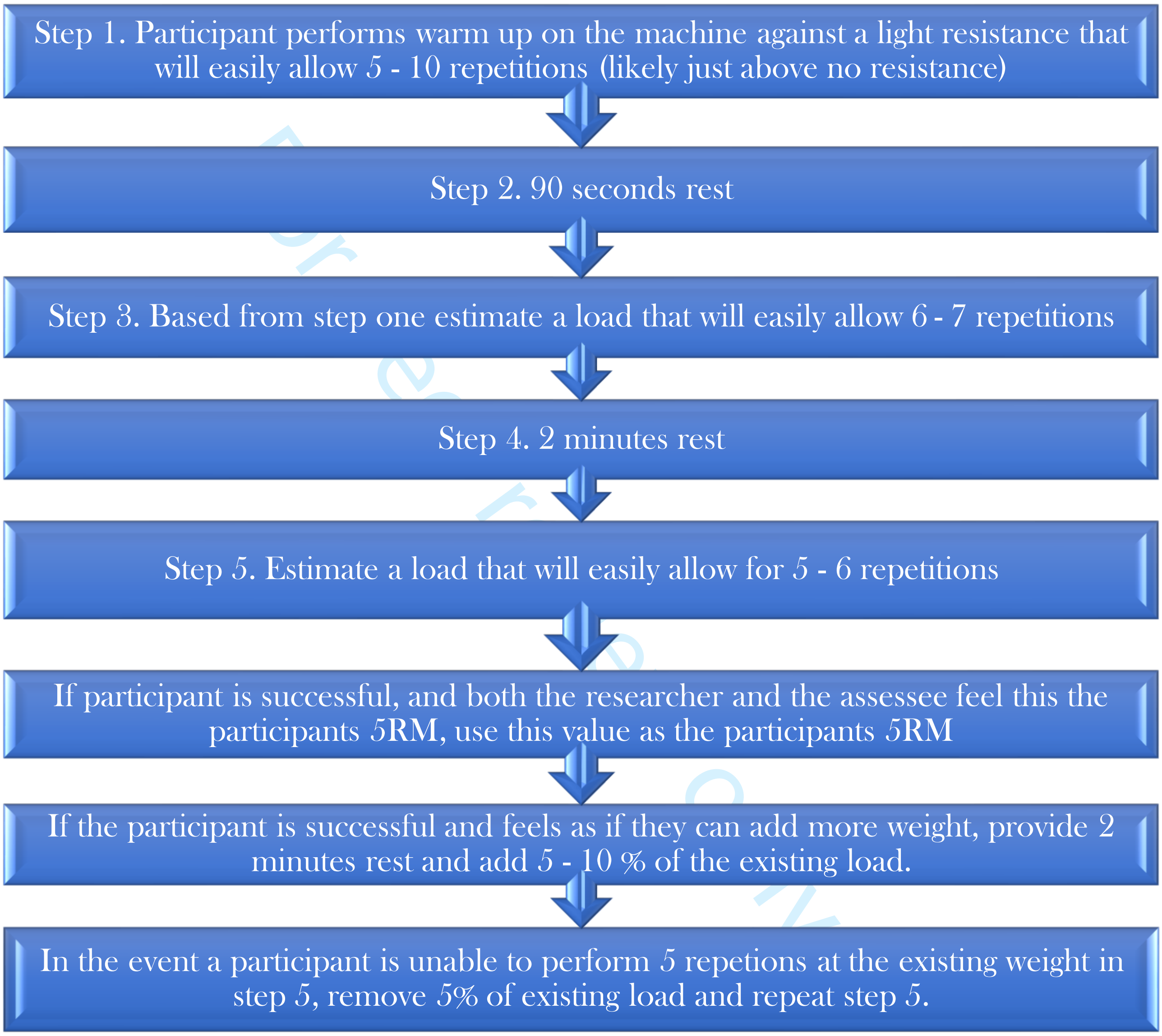


*Supplementary Material Figure 1: Five repetition maximum (5RM) assessment protocol adapted from Haff, G Gregory, Triplett, 2015*

UNIVERSITY OF BIRMINGHAM

# Seated Physical Activity in Ageing (SPAA)

## Participant Information Sheet

## I. What is this study about?

This research study will assess whether it is feasible to conduct two seated physical activity interventions for frail older adults within a short-stay hospital ward setting. The intervention will run five days per week (35 mins per session) for two weeks. We are interested in the effects on health and wellbeing.

The specially-adapted chair-based physical activity interventions are:

**Move It Or Lose It (MIOLI)**

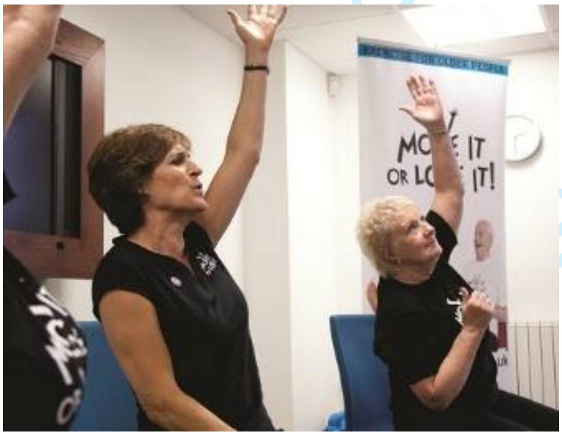

- An established chair-based physical activity programme for older adults.
- Exercises performed will be related to strength, balance, fitness and flexibility.
- The exercises are aimed to increase function and independence in older adults
- All exercises performed are chair-based, so they can be performed while sitting or standing (with, or without, chair support).

**Resistance Training Intervention**

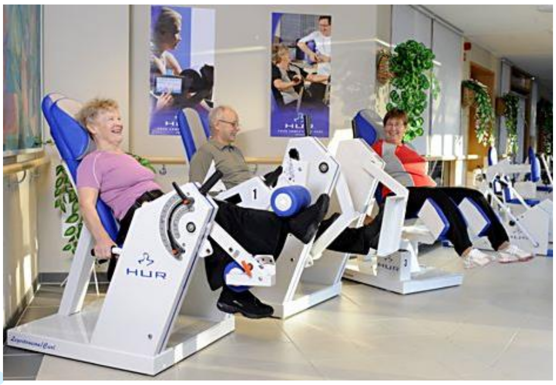


- Specially adapted resistance training machines for older adults.
- They are comfortable, easy to use, and are pneumatic (work on air-based resistance)
- They allow the difficulty to be increased in small amounts (100 grams - similar to the weight of a small apple).
- The machines work by scanning your wristband on a touch screen, and pressing buttons to increase or decrease difficulty

## II. Who is organising and conducting this study?

The Physical Activity and Nutritional INfluences In ageing (PANINI) project research group at the University of Birmingham (School of Sport Exercise and Rehabilitation Sciences), in collaboration with the Queen Elizabeth Hospital Birmingham.

Research Team: Mr. Paul Doody (Email: p.d.doody@bham.ac.uk. Phone: 0121 414 4125)
Professor Anna Whittaker (Email: a.c.whittaker@bham.ac.uk. Phone: 0121 414 4121)
Professor Janet Lord (Email: j.m.lord@bham.ac.uk. Phone: 0121 371 3234)
Dr. Thomas Jackson PhD (Consultant Geriatrician) (Email: t.jackson@bham.ac.uk)
Dr. Zoe Wyrko (Consultant Geriatrician (Email: zoe.wyrko@uhb.nhs.uk)



For peer review only - http://bmjopen.bmj.com/site/about/guidelines.xhtml

## III. Why have I been approached?

The Harborne ward is actively involved in research in geriatric populations, and encourages patient involvement in research to improve care.

In order to be eligible for the study, you must be:

- A patient / resident on the Harborne Ward
- ≥ 65 years old,
- Have the capacity to speak and read in English,
- Not currently terminally ill
- Not currently taking part in any other research study which could affect the findings of this present study
- Frail* (Possessing any three of the following criteria):
  - Low levels of handgrip strength,
  - Unintentional weight loss,
  - Low levels of physical activity,
  - Slow walking speed, and
  - Self-reported exhaustion

*If you would like to participate but are unsure if you are eligible based on the criteria above, do not worry, as these criteria will be assessed by the research team prior to enrolment once you have expressed your interest.

We will also liaise with your clinical care team to determine if you will be expected to stay on the ward for a length of time that would enable your participation in the study, and to ensure that you do not have any condition which could potentially exclude you from the study, such as severe sensory impairments.

## IV. What will happen to me if I take part?

Your participation will be up to 3 weeks, including testing before and after the physical activity interventions themselves. There will be no changes to your standard care.

<u>**Before the intervention:**</u> Up to four days before the intervention, you will be asked to undergo a series of measurements including testing if you are eligible for the study (1 hour), relating to your physical, psychological, emotional and social health and physical ability. These will be assessed through tests, questionnaires, and a blood sample consisting of three small tubes (16ml total) to measure immune function. These will take about 3 hours in total, across two 1 hour 30-minute sessions over the four days. Although we would ideally like you to complete all of the assessments, if you feel it is too much, we will prioritise certain tests.

<u>**During the intervention:**</u> You will then be allocated into one of the two physical activity interventions (which intervention depends on the dates you are resident in the ward). You will be asked to complete 10 training sessions throughout two weeks within the physical activity intervention you have been allocated to. Each training session will last approximately 35 minutes, and all training sessions will take place on the Harborne ward.

<u>**After the Intervention:**</u> After the intervention, you will be asked to complete the same measurements as before (tests of function, questionnaires, and a blood sample) in order to assess the impact of the intervention, which again will be split into two sessions, each about 1 hour 30 minutes. You will also be asked to take part in an interview (20 minutes) with the researcher, audio-recorded with your permission. During this you will be able


For peer review only - http://bmjopen.bmj.com/site/about/guidelines.xhtml

to tell us your opinion of the intervention, what you enjoyed about it the study, and where it could be improved, which will allow us to adapt the intervention based on your feedback, which we would greatly appreciate. After transcription of the interviews the audio recording will be destroyed. The researchers within this study will also request limited access to your medical notes to allow the research team to assess any factors (injuries, medications, diseases) which might affect the results of the study. Information from your patient notes will be stored confidentially and separately from your personal details.

## V. Do I have to take part?

Involvement is purely voluntary and you are free to withdraw from the study at any point without giving an explanation. There will be no negative consequences of withdrawing from the study in terms of your care. If you withdraw we would like to still analyse the data we have collected thus far.

If you express your interest in the study, a member of the research team will then meet with you to provide you with more information on the study, and to answer any questions you may have. If you states that you would not like to be involved in the research and would also not like to be contacted in the future with relation to participation, then you will not be approached by the researchers.

## VI. What are the possible benefits of taking part?

Physical activity has been shown to produce positive health benefits in older people. Additionally, physical activity interventions have been suggested as potentially offering the best form of treatment for frail older adults. Frailty can have an enormous impact on an individual's life, in addition to the lives of their friends and family. If this current study proves feasible and is found to have a positive impact, this research has the potential to have positive implications in the advancement of the hospital care of frail older adults.

## VII. What are the possible risks/side effects of taking part?

Physical activity in older people has a relatively small risk of adverse effects, typically mild muscle soreness. Due to the nature of the interventions (physical activity), it is possible that you may experience mild muscle soreness associated with physical activity, however this is a normal adaptation to exercise. In order to minimise the risk of Delayed Onset Muscle Soreness (DOMS – a more severe form of muscle soreness with a delayed onset), the interventions have been developed in a progressive manner. Additionally, the interventions will be carried out on the Harborne ward; with your care team in close proximity at all times.

## VIII. Confidentiality and data protection

Your identity or other personal information will be kept confidential. You will be assigned an ID number under which all study information will be stored on an encrypted and password protected computer/laptop at the University of Birmingham. Physical data (e.g. questionnaires) will be identifiable only by ID number and stored in a locked filing cabinet at the University of Birmingham, accessible only by the research team. Your personal information (name, D.O.B.) and your consent form will be stored securely in a separate locked filing cabinet. Data will be stored securely and confidentially for 10 years and anonymized data will also be entered into a European 'PANINI' database that this project is part of. Blood samples will be stored in approved facilities at the University of Birmingham for three years and **optionally** may be analysed in future ethically approved research across the PANINI network. As part of the PANINI project we would like to send the smallest of the three blood sample tubes to the University of Bologna in Italy to be tested by specialists there for any potential alterations in genetic markers from the physical activity intervention (optional). The University of Birmingham has public liability insurance covering its research.


For peer review only - http://bmjopen.bmj.com/site/about/guidelines.xhtml

## IX. Funding

This study has been funded by the European Commission as part of a European – wide innovate training network (ITN), consisting of Marie Curie Research Fellows / PhD students.

## X. What next?

If you say that you are interested in taking part in the study to your clinical care team, the research team will come to answer any questions you may have, check you are eligible and enrol you into the study. If you do not state that you are either interested/not in the study, the research team will ask you about whether or not you would be potentially interested in taking part. If you would not like to take part, then please tell your care team and/or the research team and you will not be contacted further about participation.

If you agree to take part, we will ask you to sign an informed consent form, outlining that you have read this information sheet and that you are aware of what the study involves. You can either sign this straight away, or take up to 24 hours to make up your mind. After this, you will be assessed for the eligibility criteria, and then begin the testing as soon as possible and then start the intervention.

If you have concerns about this study please contact the study research team or your care team to discuss this. For independent advice, you can contact your local Patient Advice and Liaison Service (PALS) (0121 371 3280), or the study sponsor (the University of Birmingham, who have given this study the sponsor registration number: RG_16-172):

Dr. Sean Jennings, Head of Research Ethics and Governance, University of Birmingham, Edgbaston, Birmingham, United Kingdom. Telephone: 0121 415 8011, Email: s.jennings@bham.ac.uk

Thank you very much for your help

For peer review only


For peer review only - http://bmjopen.bmj.com/site/about/guidelines.xhtml

Informed Consent Form
Seated Physical Activity in Ageing (SPAA)
PANINI (Physical Activity and Nutritional INfluences In ageing) Project

UNIVERSITY OF BIRMINGHAM

# Seated Physical Activity in Ageing (SPAA)

## II. Consent

Please complete the following table. Read each of the following statements. If you agree to the statement please put your **initials** in the box. If you do not agree to the statement please leave the box blank. Please note any boxes which are not marked as optional, are compulsory for initial enrollment into the study.

| | | |
|---|---|---|
| I confirm that I have read the Participant Information Sheet (Version 4 – 10/01/2018), and that I fully understand the information provided, and have had the opportunity to ask questions, and have received satisfactory answers. | | |
| I understand that I will receive a unique ID number, under which all information collected about me will be stored confidentially, either on an encrypted and password protected laptop and computer, or in the case of physical information, within a locked filing cabinet, at the University of Birmingham, accessible only to the research team. | | |
| I consent to giving a blood sample both pre- and post-intervention. I am aware that samples will be stored in a secure laboratory freezer at the University of Birmingham. | | |
| I consent to giving information related to my personal data (name, age, occupation, marital status) psychological, cognitive, emotional, social health and functional capacity through questionnaires and tests. | | |
| I agree to the audio-taping of an interview between myself and the researcher (where I can give my opinion on the study) and understand recordings will be destroyed after transcription. | | |
| I understand that my data might be used in a written report, but will not be identifiable as me. | | |
| I consent to all data obtained about me being stored for a period of 10 years as part of the PANINI project shared dataset, and that these data will be unidentifiable as mine but may be used in future ethically approved research. After 10 years it will be destroyed. | | |
| I understand that involvement within this research project is purely voluntary and that I am free to withdraw from the study at any point which I wish to do so with no repercussion of any form for myself, or my quality of care. | | |
| I understand that relevant sections of the data collected about me during the study may be looked at by ethics regulatory authorities or appropriate individuals from the University of Birmingham during a potential monitoring visit. I give permission for these individuals to have access to my linked anonymized data. | | |
| I consent to take part in this above titled research study of my own volition | | |
| **Optional (Please circle yes / no)** - I consent to members of the research team having limited access to my medical notes in order to allow information to be obtained relating to various morbidities (injuries, ailments, diseases) and medications. | Yes<br>No | |
| **Optional (Please circle yes / no)** - I consent to my blood samples being stored as part of the PANINI project shared dataset for three years, and that these data will be unidentifiable as mine, but may be used in future ethically approved research. | Yes<br>No | |

**Participants' Signature:** ____________________

**Name in Block Capitals:** ____________________

**Date:** ____________________

**Signature of person taking consent:** ____________________

**Name in Block Capitals:** ____________________

**Date:** ____________________


For peer review only - http://bmjopen.bmj.com/site/about/guidelines.xhtml

SPIRIT
STANDARD PROTOCOL ITEMS: RECOMMENDATIONS FOR INTERVENTIONAL TRIALS

SPIRIT 2013 Checklist: Recommended items to address in a clinical trial protocol and related documents*

| Section/item | Item No | Description | Addressed on page number |
|---|---|---|---|
| **Administrative information** | | | |
| Title | 1 | Descriptive title identifying the study design, population, interventions, and, if applicable, trial acronym | Page 1 (Lines 1-5) |
| Trial registration | 2a | Trial identifier and registry name. If not yet registered, name of intended registry | Page 2 (Line 36), Page 18 (Lines 417) |
| | 2b | All items from the World Health Organization Trial Registration Data Set | N/A |
| Protocol version | 3 | Date and version identifier | Page 1 -28 (Header) |
| Funding | 4 | Sources and types of financial, material, and other support | Page 18 (Lines 423 - 427) |
| Roles and responsibilities | 5a | Names, affiliations, and roles of protocol contributors | Page 1 (Lines 6 -1 – 13), Page 19 (Lines 429 - 432) |
| | 5b | Name and contact information for the trial sponsor | Page 17 (Lines 394 - 396) |
| | 5c | Role of study sponsor and funders, if any, in study design; collection, management, analysis, and interpretation of data; writing of the report; and the decision to submit the report for publication, including whether they will have ultimate authority over any of these activities | N/A |

For peer review only

| | | | |
|---|---|---|---|
| | 5d | Composition, roles, and responsibilities of the coordinating centre, steering committee, endpoint adjudication committee, data management team, and other individuals or groups overseeing the trial, if applicable (see Item 21a for data monitoring committee) | Page 12 (Lines 274 – 280) |
| **Introduction** | | | |
| Background and rationale | 6a | Description of research question and justification for undertaking the trial, including summary of relevant studies (published and unpublished) examining benefits and harms for each intervention | Page 3-5 (Lines 46 – 96) |
| | 6b | Explanation for choice of comparators | Page 4/5 (Lines 67 - 96) |
| Objectives | 7 | Specific objectives or hypotheses | Page 5 (Lines 98 - 114) |
| Trial design | 8 | Description of trial design including type of trial (eg, parallel group, crossover, factorial, single group), allocation ratio, and framework (eg, superiority, equivalence, noninferiority, exploratory) | Page 6/7 (Lines 116 - 153) |
| **Methods: Participants, interventions, and outcomes** | | | |
| Study setting | 9 | Description of study settings (eg, community clinic, academic hospital) and list of countries where data will be collected. Reference to where list of study sites can be obtained | Page 4 (Line 76), Page 5 (Lines 106 - 107), Page 6 (Lines 121 - 123) |
| Eligibility criteria | 10 | Inclusion and exclusion criteria for participants. If applicable, eligibility criteria for study centres and individuals who will perform the interventions (eg, surgeons, psychotherapists) | Page 7 (Lines 153 - 163) |
| Interventions | 11a | Interventions for each group with sufficient detail to allow replication, including how and when they will be administered | Page 7/8 (Lines 165 - 182) |
| | 11b | Criteria for discontinuing or modifying allocated interventions for a given trial participant (eg, drug dose change in response to harms, participant request, or improving/worsening disease) | Page 17/18 Lines 403 - 414) |


For peer review only - http://bmjopen.bmj.com/site/about/guidelines.xhtml

| | | | |
|---|---|---|---|
| | 11c | Strategies to improve adherence to intervention protocols, and any procedures for monitoring adherence (eg, drug tablet return, laboratory tests) | Page 12 (Lines 266 - 272) |
| | 11d | Relevant concomitant care and interventions that are permitted or prohibited during the trial | Page 6/7 Lines 125 - 153) |
| Outcomes | 12 | Primary, secondary, and other outcomes, including the specific measurement variable (eg, systolic blood pressure), analysis metric (eg, change from baseline, final value, time to event), method of aggregation (eg, median, proportion), and time point for each outcome. Explanation of the clinical relevance of chosen efficacy and harm outcomes is strongly recommended | Page 8-11 (Lines 184 - 248) |
| Participant timeline | 13 | Time schedule of enrolment, interventions (including any run-ins and washouts), assessments, and visits for participants. A schematic diagram is highly recommended (see Figure) | Page 11/12 (Lines 250 - 272) |
| Sample size | 14 | Estimated number of participants needed to achieve study objectives and how it was determined, including clinical and statistical assumptions supporting any sample size calculations | Page 12/13 (Lines 282 - 286) |
| Recruitment | 15 | Strategies for achieving adequate participant enrolment to reach target sample size | Page 13/14 (Lines 288 - 316) |

**Methods: Assignment of interventions (for controlled trials)**

Allocation:

| | | | |
|---|---|---|---|
| Sequence generation | 16a | Method of generating the allocation sequence (eg, computer-generated random numbers), and list of any factors for stratification. To reduce predictability of a random sequence, details of any planned restriction (eg, blocking) should be provided in a separate document that is unavailable to those who enrol participants or assign interventions | Page 7 Lines 148 - 149) |
| Allocation concealment mechanism | 16b | Mechanism of implementing the allocation sequence (eg, central telephone; sequentially numbered, opaque, sealed envelopes), describing any steps to conceal the sequence until interventions are assigned | N/A |
| Implementation | 16c | Who will generate the allocation sequence, who will enrol participants, and who will assign participants to interventions | N/A |

| | | | |
|---|---|---|---|
| Blinding (masking) | 17a | Who will be blinded after assignment to interventions (eg, trial participants, care providers, outcome assessors, data analysts), and how | N/A |
| | 17b | If blinded, circumstances under which unblinding is permissible, and procedure for revealing a participant's allocated intervention during the trial | N/A |
| **Methods: Data collection, management, and analysis** | | | |
| Data collection methods | 18a | Plans for assessment and collection of outcome, baseline, and other trial data, including any related processes to promote data quality (eg, duplicate measurements, training of assessors) and a description of study instruments (eg, questionnaires, laboratory tests) along with their reliability and validity, if known. Reference to where data collection forms can be found, if not in the protocol | Page 8 – 12 (Line 194-285) |
| | 18b | Plans to promote participant retention and complete follow-up, including list of any outcome data to be collected for participants who discontinue or deviate from intervention protocols | N/A |
| Data management | 19 | Plans for data entry, coding, security, and storage, including any related processes to promote data quality (eg, double data entry; range checks for data values). Reference to where details of data management procedures can be found, if not in the protocol | Page 13 (Line 287-293) |
| Statistical methods | 20a | Statistical methods for analysing primary and secondary outcomes. Reference to where other details of the statistical analysis plan can be found, if not in the protocol | Page 14 – 16 (Lines 331-373) |
| | 20b | Methods for any additional analyses (eg, subgroup and adjusted analyses) | |
| | 20c | Definition of analysis population relating to protocol non-adherence (eg, as randomised analysis), and any statistical methods to handle missing data (eg, multiple imputation) | N/A |
| **Methods: Monitoring** | | | |
| Data monitoring | 21a | Composition of data monitoring committee (DMC); summary of its role and reporting structure; statement of whether it is independent from the sponsor and competing interests; and reference to where further details about its charter can be found, if not in the protocol. Alternatively, an explanation of why a DMC is not needed | Page 13 (Lines 287-293) |
| | 21b | Description of any interim analyses and stopping guidelines, including who will have access to these interim results and make the final decision to terminate the trial | N/A |



For peer review only - http://bmjopen.bmj.com/site/about/guidelines.xhtml

| | | | |
|---|---|---|---|
| Harms | 22 | Plans for collecting, assessing, reporting, and managing solicited and spontaneously reported adverse events and other unintended effects of trial interventions or trial conduct | Page 17/18 (Line 403 - 414) |
| Auditing | 23 | Frequency and procedures for auditing trial conduct, if any, and whether the process will be independent from investigators and the sponsor | N/A |
| **Ethics and dissemination** | | | |
| Research ethics approval | 24 | Plans for seeking research ethics committee/institutional review board (REC/IRB) approval | Page 17 (Line 391 – 396) |
| Protocol amendments | 25 | Plans for communicating important protocol modifications (eg, changes to eligibility criteria, outcomes, analyses) to relevant parties (eg, investigators, REC/IRBs, trial participants, trial registries, journals, regulators) | N/A |
| Consent or assent | 26a | Who will obtain informed consent or assent from potential trial participants or authorised surrogates, and how (see Item 32) | Page 13/14 (Line 300 - 316) |
| | 26b | Additional consent provisions for collection and use of participant data and biological specimens in ancillary studies, if applicable | Page 16 (Lines 376 - 380) |
| Confidentiality | 27 | How personal information about potential and enrolled participants will be collected, shared, and maintained in order to protect confidentiality before, during, and after the trial | Page 16 (Lines 361 - 380) |
| Declaration of interests | 28 | Financial and other competing interests for principal investigators for the overall trial and each study site | Page 19 (Lines 434 - 435) |
| Access to data | 29 | Statement of who will have access to the final trial dataset, and disclosure of contractual agreements that limit such access for investigators | Page 16 (Lines 361 - 380) |
| Ancillary and post-trial care | 30 | Provisions, if any, for ancillary and post-trial care, and for compensation to those who suffer harm from trial participation | N/A |
| Dissemination policy | 31a | Plans for investigators and sponsor to communicate trial results to participants, healthcare professionals, the public, and other relevant groups (eg, via publication, reporting in results databases, or other data sharing arrangements), including any publication restrictions | Page 17 (Lines 387 – 388, Lines 398 – 401) |

For peer review only

| | | | |
|---|---|---|---|
| | 31b | Authorship eligibility guidelines and any intended use of professional writers | Page 19 (Lines 429 - 432) |
| | 31c | Plans, if any, for granting public access to the full protocol, participant-level dataset, and statistical code | Page 16 (Lines 376 – 380) |
| **Appendices** | | | |
| Informed consent materials | 32 | Model consent form and other related documentation given to participants and authorised surrogates | Supplementary files |
| Biological specimens | 33 | Plans for collection, laboratory evaluation, and storage of biological specimens for genetic or molecular analysis in the current trial and for future use in ancillary studies, if applicable | Page 10 (Lines 233 – 235), Page 11 (Lines 243 – 248) |

*It is strongly recommended that this checklist be read in conjunction with the SPIRIT 2013 Explanation & Elaboration for important clarification on the items. Amendments to the protocol should be tracked and dated. The SPIRIT checklist is copyrighted by the SPIRIT Group under the Creative Commons "Attribution-NonCommercial-NoDerivs 3.0 Unported" license.

For peer review only

BMJ Open

# Assessing the feasibility and impact of specially adapted exercise interventions, aimed at improving the multi-dimensional health and functional capacity of frail geriatric hospital inpatients: protocol for a feasibility study.



| | |
|---|---|
| Journal: | *BMJ Open* |
| Manuscript ID | bmjopen-2019-031159.R2 |
| Article Type: | Protocol |
| Date Submitted by the Author: | 20-Sep-2019 |





| | |
|---|---|
| Complete List of Authors: | Doody, Paul; University of Birmingham, School of Sport, Exercise and Rehabilitation Sciences<br>Lord, Janet; University of Birmingham, NIHR Surgical Reconstruction & Microbiology Research Centre<br>Greig, Carolyn; School of Sport, Exercise and Rehabilitation Sciences, University of Birmingham, Edgbaston, Birmingham, B15 2TT, UK; MRC-Arthritis Research UK Centre for Musculoskeletal Ageing Research<br>Whittaker, Anna; University of Birmingham, School of Sport, Exercise and Rehabilitation Sciences; University of Stirling |





| | |
|---|---|
| <b>Primary Subject Heading</b>: | Sports and exercise medicine |
| Secondary Subject Heading: | Geriatric medicine, Rehabilitation medicine |
| Keywords: | Elderly, Exercise, Frail, Older adults, inpatient, geriatric |
| | |



SCHOLARONE™
Manuscripts

Protocol_Manscript_V3.0_02/09/2019

# Assessing the feasibility and impact of specially adapted exercise interventions, aimed at improving the multi-dimensional health and functional capacity of frail geriatric hospital inpatients: protocol for a feasibility study.

Paul Doody[1], Janet M. Lord[2,3], Carolyn A. Greig[1,2,3], Anna C. Whittaker[1,4]

[1] School of Sport, Exercise and Rehabilitation Sciences, University of Birmingham, United Kingdom

[2] MRC-Arthritis Research UK Centre for Musculoskeletal Ageing Research, Institute of Inflammation and Ageing, University of Birmingham, United Kingdom

[3] NIHR Birmingham Biomedical Research Centre, University Hospitals Birmingham NHS Foundation Trust and the University of Birmingham, United Kingdom

[4] Faculty of Health Sciences and Sport, University of Stirling, United Kingdom

Corresponding author: Mr. Paul Doody (p.d.doody@bham.ac.uk, +44 (0) 121 414 4125), School of Sport Exercise and Rehabilitation Sciences (G35), University of Birmingham, United Kingdom.

For peer review only

## Abstract

**Background:** Frailty is a common and clinically significant condition in older adults, predominantly due to its association with adverse health outcomes such as hospitalisation, disability and mortality. Exercise interventions have been shown to be a beneficial treatment for frailty. However, more high-quality studies are needed to assess the feasibility and impact of these interventions in frail geriatric populations within different settings, and their impact on broader aspects of health and wellbeing.

**Methods and analysis:** This study will utilise a 2-week, interventional, independent measures research design in order to assess the feasibility and impact of two specially adapted exercise training interventions (a specially adapted resistance training intervention, and Move It Or Lose It: an established community-based exercise intervention for older adults) aimed at improving the multi-dimensional health and functional capacity of frail geriatric hospital inpatients.

**Ethics and dissemination:** This study has received a favourable ethical opinion by the Coventry and Warwickshire NHS Research Ethics Committee and sponsorship by the University of Birmingham after review by the sponsors research governance office. The findings will be disseminated through publication in open access scientific journals, public engagement events, online via social media, conference presentations, and directly to study participants upon request.

**Trial registration:** Clinicaltrials.gov: NCT03141866.

## Strengths and limitations

- Mixed methods feasibility study employing both quantitative and qualitative research methodologies
- Specially adapted exercise interventions for frail geriatric populations
- Difficult to reach (and often excluded) participant population
- Single site study

For peer review only

## Background

Frailty is a common and clinically significant condition within geriatric populations (1), predominantly due to its association with adverse health outcomes such as hospitalization, disability and mortality (1-6). Although the exact prevalence of frailty within this population is poorly defined due to the lack of a single standardised operational definition for the classification of frailty, it is generally believed that the prevalence of frailty amongst community-dwelling older adults ranges between 7.0 – 16.3% (1,7). Presently there are no well-evidenced, pooled estimates of the overall prevalence of frailty within geriatric hospital inpatients. However, there are several studies which have primarily aimed to produce estimates of the prevalence of frailty within this population (8-15). Although there is an evident need for more robust research to thoroughly assess the overall prevalence of frailty within this setting, through analysis of these existing studies, dependent upon the criteria utilised, the prevalence of frailty in geriatric hospital inpatients ranges from approximately 27 – 94%. In the five of the eight studies which utilised the Fried Frailty phenotype (2) as the operational definition of frailty, there is a narrower range (27–48.5%), with a mean prevalence of frailty across the five studies of 37.5±6.76% (12,16-19).

Although there is no one standardised and universally utilised operational definition of frailty, one of the most commonly utilised is the Fried frailty phenotype (2). This proposes that frailty be defined as a clinical syndrome in which three or more of the five following criteria are present: unintentional weight loss (≥ 10lbs in the past year), self- reported exhaustion, weakness (grip strength), slow walking speed and low levels of physical activity.

Exercise interventions have been proposed as potentially offering the best form of treatment for frail older adults (20); with exercise shown to be a significantly beneficial treatment for this population with regard to multiple components of health, and even shown to mediate the reversal of frailty in some cases (21-24). However, while there is evidence of the benefits of exercise relating to the prevention, treatment, and

reversal of frailty, it is universally noted that there needs to be more high quality studies within this area to truly assess the impact of exercise in frail geriatric populations within different settings, particularly relating to its effects on broader aspects of health and well-being (1).

This present study will assess the feasibility and efficacy of short duration (2-week), intensive (five-days per week), specially adapted exercise interventions within a delayed transfer of care hospital ward setting. Feasibility will relate to the eight main areas of focus for feasibility studies (25), while efficacy will be assessed through limited efficacy testing of the impact of the interventions on the secondary dependent variables relating to multi-dimensional health and functional capacity.

Such research is very timely and pertinent, as current demographic trends indicate that by the year 2030 almost one in six of the European population will be aged 60 or over, and the number of older people will grow to 247 million by 2050, representing a 35% increase from 2017, with one in four older adults being over 85 by 2040 (26). This coupled with continual progressive declines in the rate of physical activity, at all stages of the lifespan (27), leaves the population particularly susceptible to the development of disease and co-morbidities associated with a lack of physical activity and an increase in sedentary behaviour (28). Moreover acute hospital admission for older adults are associated with further loss of physical activity and represent a period of increased susceptibility to sarcopenia and frailty (29). Frailty is associated with longer stay and increased rates of mortality in hospitalised older adults, as well as serving as a predictor of readmission (12,30). Therefore, there is an urgent need to examine the effect of such interventions within this setting, and whether these interventions can be employed to improve various aspects of health in frail older populations in inpatient hospital ward settings, as well as their efficacy in specifically treating, preventing and reversing frailty. Preliminary research has shown some success in the implementation of exercise interventions to reverse functional decline in general geriatric inpatient populations (31), however,

For peer review only

to the authors' knowledge this present study is the first to attempt such an intervention in frail delayed transfer of care patients.

# Methods and analysis

## Aims and objectives

The primary aim of this study is to assess the feasibility of a proposed future trial in this setting, which aims to assess the impact of specially adapted exercise interventions on the physiological, psychological, cognitive, social, and emotional health, and functional capacity of frail geriatric populations within a hospital ward setting; recognising health as a multi-factorial concept incorporating multiple inter-related dimensions. The secondary aim of this feasibility study is to assess the potential efficacy of the interventions on the primary dependent variables of the proposed future clinical trial within this setting.

The primary and secondary aims of this study will be achieved through the sequential achievement of the following objectives: 1) Recruitment of eligible participants from the Harborne Ward of the Queen Elizabeth Hospital Birmingham, Mindelsohn Way, Birmingham, United Kingdom. 2) Baseline assessment of the secondary dependent variables related to multi-dimensional health. 3) Assessment of the feasibility of the study as it relates to the eight-primary areas of focus for feasibility studies (acceptability, demand, implementation, practicality, adaptation, integration, expansion and limited-efficacy testing) (25). 4) Post-intervention assessment of all primary and secondary dependent variables.

The research questions of this study relate to the eight aforementioned areas of focus of this feasibility study, incorporating the following questions relating to the feasibility and efficacy of the study within this setting: Can it work? Will it work? Does it work? (25) (Table 1).

## Design overview

This feasibility study will utilise a 2-week, interventional, independent measures research design (Figure 1).

*(Insert Figure 1)*

For peer review only

A variation of a stepped-wedged design / rolling recruitment will be utilised, with the interventions being conducted multiple times over the course of several months in order to maximise the potential sample size due to constraints of the setting: a delayed transfer of care hospital ward for patients prior to official discharge, with the majority of patients residing on the ward for > 3 weeks, and approximately 25 patients on the ward at any given time.

The independent variables of the study will comprise of two exercise training interventions: a specially adapted machine-based resistance training intervention, and Move It Or Lose It (MIOLI), an established community-based exercise intervention for older adults. A control group will be utilised within the proposed future clinical trial but will not be utilised within this feasibility study as one of the primary purposes of this study is to assess the feasibility, and to a limited degree the efficaciousness, of the interventions within this setting. (Figure 2). *(Insert Figure 2)*

In order to ensure this present study is as scientifically valid as possible a number of precautions have been taken to protect the internal and external validity of the study within its methodological design:

First, for each participant, all testing procedures (baseline and post-intervention (2-weeks) will be conducted at approximately the same time of day (+/- two hours). This will be controlled in order to protect the findings of the study from changes in the dependent variables which may be attributable to circadian variation rather than manipulation of the independent variables (32). The hypothesis of the study will not be divulged to participants prior to or during the conduction of the study in order to control for any potential degree of demand characteristics; a scenario where participants alter their behaviour and/or answers, in order to align with what they believe is potentially the 'desired' outcome of the study. All dependent variable testing sessions will take place at least 24 hours after the cessation of the previous training session for each participant. This will be implemented to ensure acute fatigue does not become a contributing factor to the results of the study, specifically relating to the secondary dependent variables, but also the feasibility of such practice during a proposed future clinical trial. The order in which dependent variables are tested will be

counter-balanced throughout the study at each assessment timepoint in order to attempt to protect the study from practice effects, especially order effects, where a participant has been exposed to a specific order of testing before and as such performs better on subsequent testing procedures of the same material. Additionally, only one intervention will be run at a given time, protecting against potential subconscious selection bias amongst the research team relating to group allocation of participants. Finally, in order to increase the external validity of the study, eligibility criteria will be kept as minimalistic as possible (within the limits of safety and reason), in order to allow as inclusive a proportion of this population as possible, and in such producing findings which are applicable to not only those within the study, but to the greater population of frail geriatric hospital inpatients and particularly those within delayed transfer of care settings.

## Eligibility

This study is open to both men and woman whom meet the following eligibility criteria: inpatient on the Harborne ward of the Queen Elizabeth hospital Birmingham, Mindelsohn Way, Birmingham, United Kingdom; ≥ 65 years of age; frail according to the Fried Frailty Phenotype criteria (2); ability to speak and read in English; not currently taking part in any other clinical trial which could potentially impact upon or influence the findings of this study; not currently terminally ill with life expectancy less than the duration of the study; no severe sensory impairment which would profoundly impact upon ability to undergo the intervention, even once appropriate adaptations have been made; anticipated by their care team to remain on the ward for approximately 21 days post enrolment into the study.

## Interventions

All participants will undergo 10 sessions in total throughout the two-week intervention in either one of the two interventions. All session will be conducted individually under the guidance of a qualified trainer. A maximum of one session will be performed each day, and sessions will not be performed on any more than a maximum of three consecutive days throughout the duration of the study (Table 2).

For peer review only

## Exercise Intervention 1: Specially-adapted resistance training intervention

This intervention will be comprised of an intensive (five days per week), short duration (2 week), approximately 35 minutes per session, machine-based resistance training intervention. The exercises performed will specifically target the lower limbs through a combination of multi-joint strength and power training utilising a leg press and leg extension machine. (Figure 3). *(Insert Figure 3)*.

The maximal strength reference value (% 1RM), duration (35 minutes), type of exercise (multi-joint), loadings (60% 1RM (Power), 80% 1RM (Strength)), and volume (3 sets, 5 – 8 repetitions) are largely consistent with the position statement from the National Strength and Conditioning Association (NSCA) regarding resistance training in older adults (Fragala et al. 2019).

An outline of the protocol for each session can be found in Figure 4. *(Insert Figure 4)*

## Exercise Intervention 2: Move It Or Lose It (MIOLI)

This multi-component intervention will comprise of an intensive (five days per week), short duration (2 week), approximately 35 minutes per session, chair-based exercise intervention. The exercises conducted will relate to strength, power, flexibility and aerobic capacity, consistent with the established Move It Or Lose It (MIOLI) flexibility, aerobic capacity, balance and strength (FABS) training program for community-dwelling older adults. The exercises performed will predominantly targeting the lower limbs, but also incorporate the upper body and core.

An outline of the protocol for each session can be found in Figure 5. *(Insert Figure 5)*

## Dependent Variables

For peer review only

## Primary dependent variables

The primary dependent variables will relate to the eight primary areas of focus of feasibility studies (25) (utilised to establish the feasibility of a proposed future clinical trial within this setting), relating to: acceptability, demand, implementation, practicality, adaptation, integration, expansion and limited-efficacy testing.

These dependent variables will be assessed through semi-structured interviews with study participants and focus groups with ward staff post-intervention. Participant uptake and adherence records will also be employed throughout. These methods seek to attain answers to the following questions and parameters relating to the eight primary areas of enquiry for this feasibility study outlined in further detail in Table 1.

*(Insert Table 1 - Landscape)*

In order to enhance trustworthiness in the qualitative component of this research, several methods will be employed**:**

The researcher gathering the data will keep a reflective journal in which they will record information about themselves, their activities and the methods used. Field notes will include time, date and location, participant's actual notes, and the researcher's own questions and comments. This will lend to logging and documenting what is learned about the study, the intervention, the setting, the participants, and used to refine focus for future interviews through assessing the following questions: What is important? What is it I need to find out more about? What would I want to focus on more closely if I could do the interview again, or in future interviews? (33).

Data will be gathered from study participants and ward staff in order to collect data from multiple sources (triangulate information). This study will also employ more than one researcher to analyse the qualitative data in order to enhance triangulation and validity.

For peer review only - http://bmjopen.bmj.com/site/about/guidelines.xhtml

Enough details will be given about the participants and the setting to make decisions about the quality of the findings from the qualitative analysis. Detailed descriptions about the participants experiences and the setting will be provided by the researcher.

In the qualitative data analysis, clarification of all possible researcher biases will be made known. For example, it will be articulated that the researcher is an advocate of physical activity as a means to promote health, prescribing to the theoretical and practical concept of exercise as medicine, and hence there may be some form of unconscious subjective bias in this context. However, it should also be noted that the researcher within this study is also an advocate of science to an equal or even greater extent, and as such any such bias in subjective analysis would potentially be counteracted in this sense.

Interviews - "a conversation with a purpose" (34) will be the primary method of data gathering utilised, as it enables large amounts of information to be gathered relatively quickly. Specifically, this study will employ semi-structured interviews, with open questions in a conversational format. There will be several pre-determined themes, topics and questions to be discussed, specifically relating to the eight areas of focus of the primary dependent variables of this study. All interviews and focus groups will be audio-recorded in order to facilitate future transcription. The qualitative element of this study will also explore opportunities for Patient and Public Involvement (PPI) in the research design of the proposed future clinical trial.

As this study will utilise a mixed methods research approach, employing both qualitative and quantitative research methodologies, this will facilitate the potential for elaboration and expansion of these findings of individual methodologies through complementary analysis. The qualitative aspect of this feasibility study, aimed at assessing the primary dependent variables, will predominantly take a phenomenological approach to understand the experiences of individuals involved in the study (35).

## Secondary Dependent Variables

For peer review only - http://bmjopen.bmj.com/site/about/guidelines.xhtml

The secondary dependent variables relating to multi-dimensional health (comprising the proposed primary dependent variables of the future clinical trial) will be:

Physiological*: Serum cortisol, dehydroepiandrosterone-sulphate (DHEAS), cortisol: DHEAS ratio, C-reactive protein (CRP), Interleukin 6 (IL-6), Tumor Necrosis Factor alpha (TNFα), Interferon gamma (IFNy)

Functional: Hand grip strength (Southampton protocol (36), Leg strength and power output (37,38), Short Physical Performance Battery (SPPB) (39), Katz Index of Independence in Activities of Daily Living (Katz ADL) (40), and the Fried Frailty Phenotype (2)

Psychological / Emotional: Geriatric Depression Scale (GDS) (41), Hospital Anxiety Depression Scale (HADS) (42)

Cognitive: Standardized Mini-Mental State Examination (SMMSE) (43)

Social: Interpersonal Support Evaluation List (ISEL-12) (44)

*All blood samples will be obtained through venepuncture. Serum will be analysed for the physiological dependent variables relating to cortisol, DHEAS and CRP (assessed by commercial ELISA kit). Inflammatory cytokines (IL-6, TNFα, IFNy) will be assayed using a multiplex commercial kit (R&D Systems). These specific physiological variables will be examined due to their association with the ageing process, and previous research which have both proposed and indicated that exercise is potentially capable of altering these variables (45-50).

## Data collection

Data will predominantly be collected at two main time points: baseline and post-intervention (Table 2).

*(Insert Table 2 - Landscape)*

For peer review only - http://bmjopen.bmj.com/site/about/guidelines.xhtml

**Baseline Assessment:** Participants' baseline socio-demographic and information for the secondary dependent variables will be collected between 5 – 2 days prior to intervention commencement. One repetition maximum (1RM) for the specially adapted resistance training equipment will also be assessed during this time period (after all baseline testing has been completed, and at least 24 hours after baseline testing which requires physical exertion, which may impact on the accuracy of the 1RM measurements). 1RM will be determined based on estimation from participants five repetition maximum (5RM) utilising the Epley formula: 1RM = w (1 + r/30), where 1 = weight, and r = repetitions (38). The protocol employed during obtainment of participants 5RM has been adapted from (37) (Supplementary file 1). A balance will be conducted prior to the commencement of the MIOLI intervention to determine whether participants should perform exercises standing, standing with chair support, or seated. In addition the resistance bands which participants in the MIOLI intervention will use will similarly be assessed during this period, with three options corresponding to light, moderate and high resistances, prescribed to participants based on initial ability and preference during their performance of the exercises in which the resistance training bands will be utilised.

**Post-intervention assessment:** Primary and secondary dependent variables will be assessed between 1 – 5 days post intervention cessation. All assessments will be conducted at least 24 hours post the cessation of the last training session.

Adherence rates in the intervention will be recorded as the number of repetitions completed in a set (100% required for adherence to that exercise), and the number of exercises for which there was 100% adherence. If participants meet these parameters for each exercise session, they will be considered to be in 100% adherence to the intervention. For example, if a participant adheres to 100% of the intervention for 9 sessions, but only 90% for 1 session, then they will have a 99% adherence rate. Adherence rates, whether high, or low, may signify that the interventions may have been too demanding, too easy or optimal. Information will also be collected throughout the study related to uptake and retention rates.

For peer review only

## Data monitoring

Data will be monitored by the trial management committee at monthly intervals. Prior to analysis data entry checking will be conducted for accuracy on 10% of all participants, and queries resolved through discussion with the trial management committee and access to the source documents. Data management will adhere to the PANINI data management plan, which was developed in accordance with national and European principles as part of the university research governance and European Commission research governance guidelines. Thus, data management for this project adheres to the FAIR principles (51).

## Sample size

This study aims to recruit a convenience sample of n = ~ 30 participants: 15 in each intervention. No formal power calculations were conducted due to the feasibility nature of this study. This sample size was based on initial expectations related to the obtainment of data saturation in the qualitative component of the study given its phenomenological approach (52,53).

## Identification, Consent and Recruitment

**Identification:** Patients on the ward at the commencement of the study will initially be screened by their care team for the known presence of any severe sensory impairments which would exclude them from participation. Due to the study's rolling recruitment, this initial screening will continue throughout the study as additional patients are admitted to the ward. Participant information sheets will not be distributed to patients who are deemed to be medically unfit or due for imminent discharge as identified by their care team. All other aspects of the screening process will occur after consent has been obtained. After identification, potential participants will be approached by the researcher with an information sheet related to the study and asked if they would be interested in participating, or if they would like to receive more information about the study (Supplementary file 2). The information sheet will contain all the most pertinent

For peer review only - http://bmjopen.bmj.com/site/about/guidelines.xhtml

information relating to the study and specifically what it would require from potential participants. Potential participants will be given ≥ 24 hours to consider whether or not they would like to participate.

**Consent:** At this stage potential participants will also be provided with an informed consent form and asked if they would be interested in participating (Supplementary file 3). If it is deemed that a potential participant lacks the capacity to consent, a personal consultee will be sought. A personal consultee will be someone who cares for the patient, or is interested in their welfare (but not a paid professional), and who is prepared to be consulted and give advice on what they believe the patient's wishes would be, were they to have the capacity to consent for themselves. If a personal consultee cannot be found, a nominated consultee will be sought. A nominated consultee will be someone who is familiar with the patient in a professional context and can adequately advise on whether they believe participation would be in the patients' best interest. These processes will be informed by the UK Department of Health's guidance on nominating a consultee for research involving adults who lack the capacity to consent (54); in accordance with section 32(3) of the Mental Capacity Act 2005 (55). All efforts possible will be made in this regard to include participants whom lack the capacity to consent, as intrinsically within the research team from a personal and professional perspective we would consider it unethical to exclude potential participants from participating in a study, which can potentially benefit them and their overall health status, due to the fact that they lack the capacity to consent.

**Recruitment:** Following consent being obtained from the participant themselves, or the obtainment of a declaration from a consultee, all consented participants will be screened for the remaining eligibility criteria.

## Statistical methods

### Primary Dependent Variables

Analysis of the primary dependent variables will be based on an inductive process utilising Interpretative Phenomenological Analysis (thematic analysis). Two researchers will be employed to analyse the data to increase triangulation from the analysis perspective, having already triangulated data acquisition through

For peer review only - http://bmjopen.bmj.com/site/about/guidelines.xhtml

obtainment from multiple sources (study participants and ward staff). All semi-structured interviews and focus groups will be audio-recorded. Data synthesis will be performed through verbatim transcription. The three main steps of Interpretative Phenomenological Analysis will be followed (35): 1) The generation of themes from transcripts within the areas of feasibility inquiry. As an iterative process, these themes will be continuously reviewed and adapted based on the emergence of information in subsequent transcripts 2) The collation and separation of these themes within each of the areas of feasibility inquiry 3) Written interpretation of the resultant themes within each of the areas of feasibility and their relationship to one another. At all stages within this process, reflective journal entries and field notes will be utilised to provide a more comprehensive understanding of the findings, in addition to incorporating additional feasibility information related to uptake and retention rates, and limited efficacy testing of the secondary dependent variables in the final analysis to provide a comprehensive assessment of the feasibility of the study.

**Secondary Dependent Variables**

Statistical analysis of the secondary dependent variables will be performed using IBM Statistical Package for Social Sciences (SPSS) software. These analyses will be performed as part of the limited efficacy testing regarding the potential impact of the interventions on the secondary dependent variables (proposed primary dependent variables of the future clinical trial). These analyse will provide an estimation of efficacy and provide valuable insight regarding feasibility; useful to informing the design of the future powered clinical trial. Specifically, the following statistical analysis will be utilised: 2x2 way independent measures ANOVA's (analysis' of variance consisting of two independent variables; the specially adapted resistance training intervention, and the Move It Or Lose It (MIOLI) intervention, each with two levels: baseline and post-intervention. A subsequent post-hoc test will be utilised if a significant main effect or interactions are found. Pearson product correlations will also be utilised between various socio-demographic variables (such as age and sex) and the dependent variables of this study, to assess possible relationships between differences in socio-demographic factors and changes in the dependent variables.

For peer review only

Central tendency and variability measurements consisting of parameters such as the mean, median and mode, and standard deviation and range of scores respectively, will also be utilised during the analysis of data for illustrative purpose. Significance levels will be set at 0.05 ($p \leq 0.05$), and effect sizes will be reported for all analyses. In order to establish if the assumptions of parametric statistics have been met regarding the assumption that there is a normal distribution of data, the data will be analysed for skewness and kurtosis. In the event data does not fulfill the assumptions of parametric statistics, the non-parametric equivalent of the aforementioned statistical analysis will be employed, namely the Scheirer-Ray-Hare test, and Spearman's rank-order correlations. As the quantitative component of this study has not been powered given the feasibility nature of the study, the examination of the efficacy of the intervention to impact these secondary dependent variables is limited; and interpretation of these results should be treated with caution pending the future powered clinical trial. All results will be reported with effect size and 95% confidence intervals.

## Data storage and protection

Participants' identity or other personal information will be kept confidential. Participants will be assigned a unique ID number under which all study information will be stored in a secure file on an encrypted and password protected computer and laptop at the University of Birmingham (UoB). Physical data (e.g. Case Report Forms (CRF's)) will be identifiable only by ID number and stored in a locked filing cabinet at the School of Sport, Exercise and Rehabilitation Sciences at UoB, accessible only by the research team. Participants' personal data (name, date of birth) and consent forms matching them to their ID number, will be stored securely in a locked filing cabinet, separate from all other data and/or in a password protected master sheet on an encrypted and password protected computer and laptop at UoB. All serum samples will be stored in Human Tissue Act (HTA) compliant facilities at UoB for three years then destroyed.

All hard copy data collected on CRF's will be stored in a linked-anonymised format securely for 10 years then destroyed. All personal data (consent forms, master sheet linking participant IDs to names and contact

For peer review only

details) will be stored for 10 years then destroyed. All computerised data will be archived on UoB servers in anonymised form for 10 years in the first instance in accordance with the UoB Code of Practice for Research, and the Data Protection Act (2018).

Following analysis for this specific study, all data will be anonymised and entered into a European 'PANINI' open access database that this project is part of and may be analysed in future ethically approved research across the PANINI network. The PANINI shared dataset will be made open access at the conclusion of the funding for the PANINI network including this study in 2020 and stored for at least 10 years as an open access searchable published dataset.

## Patient and Public involvement

All authors are strong proponents of patient and public involvement and engagement with research and believe the findings of the study will be important to aid the facilitation of improvements in the care of frail older hospital inpatients. Given the feasibility nature of this research, the qualitative element of this study will explore opportunities for Patient and Public Involvement (PPI) in the research design of the future clinical trial. The findings of this study will be disseminated to participants upon request, and our patient and public involvement groups.

## Ethics and dissemination

### Ethical approval and consent to participate

This study has been granted a favourable ethical opinion by the Coventry and Warwickshire NHS Research Ethics Committee (REC) (17/WM/0390) on the 12/03/2018. This study has also been sponsored by the University of Birmingham, after review by the sponsor's research governance office (sponsor registration number: RG_17-108).

For peer review only

## Dissemination

The findings of this study will be disseminated through publication of scientific papers in open access scientific journals, public engagement events, online via social media (Twitter, Instagram) and the PANINI project website (56,57), presentation at various conferences, and to study participants upon request.

## Safety reporting and monitoring

Adverse events (AE) and serious adverse events (SAE) will be monitored and if applicable recorded, by the primary researcher at each testing or exercise session and reported weekly to the principle investigator (PI) and reviewed in the case of AEs. SAEs will be reported immediately to the PI who will complete an SAE form indicating causality and severity. The chief investigator (CI) will then submit this to the sponsor's research governance office, the Research Ethics Committee (REC) and University Hospitals Birmingham (UHB) research governance office, within 24 hours. UHB local policies and Standard Operating Procedures (SOP's) for all safety reporting will also be followed by the research team. SAEs related to pre-existing conditions will not be reported. Actions following an AE or SAE would be as standard, i.e. direct referral to their clinical care team, who then may wish to treat the AE or SAE themselves or where appropriate refer the participant to another relevant medical professional, and to recommend that the participant withdraw from the study unless they have been cleared to continue exercise by their attending physician.

## Trial registration

This study has been registered on ClinicalTrials.gov under the identifier number: NCT03141866.

## Trial status

For peer review only

This trial commenced on the 03/09/2018 on the Harborne ward of the Queen Elizabeth Hospital Birmingham, with anticipated completion of data collection scheduled for 09/08/2019. This present study is a sister trail of another feasibility study; comprising of a specially adapted resistance training intervention for frail geriatric care home residents (58).

## Funding

This review has been funded by the European Commission's Horizon 2020 research and innovation programme under the Marie Sklodowska-Curie grant agreement (675003); of which PD is a Marie Sklodowska-Curie Doctoral Research Fellow, AW, JL and CAG doctoral supervisors, and AW the grants Principal Investigator.

## Author contributions

PD designed the study protocol, and associated manuscript for publication, with supervision, input and feedback from AW at all stage of the design and writing process. JL and CAG reviewed and revised the manuscript prior to publication. All authors have read and approved the final manuscript.

## Competing interests

The authors have no competing interests to declare.

## References

(1) Rodriguez-Mañas L, Fried LP. Frailty in the clinical scenario. The Lancet 2015 /02/14;385(9968):e-e9.

For peer review only

(2) Fried LP, Tangen CM, Walston J, Newman AB, Hirsch C, Gottdiener J, et al. Frailty in older adults evidence for a phenotype. The Journals of Gerontology Series A: Biological Sciences and Medical Sciences 2001;56(3):M14-M157.

(3) Gill TM, Gahbauer EA, Allore HG, Han L. Transitions between frailty states among community-living older persons. Arch Intern Med 2006;166(4):418-423.

(4) Clegg A, Young J, Iliffe S, Rikkert MO, Rockwood K. Frailty in elderly people. The Lancet 2013 March 8,;381(9868):752-762.

(5) Sternberg SA, Schwartz AW, Karunananthan S, Bergman H, Mark Clarfield A. The identification of frailty: a systematic literature review. J Am Geriatr Soc 2011;59(11):2129-2138.

(6) Sourial N, Bergman H, Karunananthan S, Wolfson C, Payette H, Gutierrez-Robledo LM, et al. Implementing Frailty Into Clinical Practice: A Cautionary Tale. J Gerontol A Biol Sci Med Sci 2013 -05-02:glt053.

(7) Collard RM, Boter H, Schoevers RA, Oude Voshaar RC. Prevalence of frailty in community-dwelling older persons: a systematic review. J Am Geriatr Soc 2012 Aug;60(8):1487-1492.

(8) Joosten E, Demuynck M, Detroyer E, Milisen K. Prevalence of frailty and its ability to predict in hospital delirium, falls, and 6-month mortality in hospitalized older patients; 24393272. BMC Geriatrics 2014;14(1).

(9) Oliveira DR, Bettinelli LA, Pasqualotti A, Corso D, Brock F, Erdmann AL, et al. Prevalence of frailty syndrome in old people in a hospital institution. Revista Latino-Americana de Enfermagem 2013 08/;21(4):891-898.

(10) Ekerstad N, Swahn E, Janzon M, Alfredsson J, Löfmark R, Lindenberger M, et al. Frailty is independently associated with short-term outcomes for elderly patients with non-ST-segment elevation myocardial infarction. Circulation 2011 Nov 29,;124(22):2397-2404.

(11) Purser JL, Kuchibhatla MN, Fillenbaum GG, Harding T, Peterson ED, Alexander KP. Identifying Frailty in Hospitalized Older Adults with Significant Coronary Artery Disease. Journal of the American Geriatrics Society 2006 November 1,;54(11):1674-1681.

(12) Khandelwal D, Goel A, Kumar U, Gulati V, Narang R, Dey AB. Frailty is associated with longer hospital stay and increased mortality in hospitalized older patients. Journal of Nutrition, Health and Aging 2012;16(8):732-735.

(13) Maguet PL, Roquilly A, Lasocki S, Asehnoune K, Carise E, Martin MS, et al. Prevalence and impact of frailty on mortality in elderly ICU patients: a prospective, multicenter, observational study. Intensive Care Med 2014 /05/01;40(5):674-682.

For peer review only

(14) Hewitt J, Moug SJ, Middleton M, Chakrabarti M, Stechman MJ, McCarthy K. Prevalence of frailty and its association with mortality in general surgery. Am J Surg 2015 Feb;209(2):254-259.

(15) Andela RM, Dijkstra A, Slaets JP, Sanderman R. Prevalence of frailty on clinical wards: Description and implications. International Journal of Nursing Practice 2010 February 1,;16(1):14-19.

(16) Joosten E, Demuynck M, Detroyer E, Milisen K. Prevalence of frailty and its ability to predict in hospital delirium, falls, and 6-month mortality in hospitalized older patients. BMC Geriatrics 2014;14(1).

(17) Oliveira DR, Bettinelli LA, Pasqualotti A, Corso D, Brock F, Erdmann AL. Prevalence of frailty syndrome in old people in a hospital institution. Rev Lat Am 2013;21(4):891-898.

(18) Purser JL, Kuchibhatla MN, Fillenbaum GG, Harding T, Peterson ED, Alexander KP. Identifying frailty in hospitalized older adults with significant coronary artery disease. J Am Geriatr Soc 2006;54(11):1674-1681.

(19) Le Maguet P, Roquilly A, Lasocki S, Asehnoune K, Carise E, Saint Martin M, et al. Prevalence and impact of frailty on mortality in elderly ICU patients: A prospective, multicenter, observational study. Intensive Care Med 2014;40(5):674-682.

(20) Theou O, Stathokostas L, Roland KP, Jakobi JM, Patterson C, Vandervoort AA, et al. The effectiveness of exercise interventions for the management of frailty: a systematic review. Journal of aging research 2011;2011.

(21) Arrieta H, Rezola-Pardo C, Gil SM, Virgala J, Iturburu M, Antón I, et al. Effects of Multicomponent Exercise on Frailty in Long-Term Nursing Homes: A Randomized Controlled Trial. J Am Geriatr Soc 2019.

(22) Tarazona-Santabalbina FJ, Gmez-Cabrera MC, Prez-Ros P, Martnez-Arnau FM, Cabo H, Tsaparas K, et al. A multicomponent exercise intervention that reverses frailty and improves cognition, emotion, and social networking in the community-dwelling frail elderly: a randomized clinical trial. Journal of the American Medical Directors Association 2016;17(5):426-433.

(23) Ng TP, Feng L, Nyunt MSZ, Feng L, Niti M, Tan BY, et al. Nutritional, physical, cognitive, and combination interventions and frailty reversal among older adults: a randomized controlled trial. Am J Med 2015;128(11):122-1236. e1.

(24) Fiatarone MA, Marks EC, Ryan ND, Meredith CN, Lipsitz LA, Evans WJ. High-Intensity Strength Training in Nonagenarians: Effects on Skeletal Muscle. ResearchGate 1990 /07/01;263(22):3029-34.

For peer review only

(25) Bowen DJ, Kreuter M, Spring B, Cofta-Woerpel L, Linnan L, Weiner D, et al. How we design feasibility studies. Am J Prev Med 2009;36(5):452-457.

(26) United Nations, Department of Economic and Social Affairs, Population Division. World Population Ageing 2017 (ST/ESA/SER.A/408). URL: https://www.un.org/en/development/desa/population/publications/pdf/ageing/WPA2017_Report.pdf. Accessed: 17th April 2019.

(27) Caspersen CJ, Pereira MA, Curran KM. Changes in physical activity patterns in the United States, by sex and cross-sectional age. Med Sci Sports Exerc 2000;32(9):1601-1609.

(28) Ford ES, Caspersen CJ. Sedentary behaviour and cardiovascular disease: a review of prospective studies. Int J Epidemiol 2012:dys078.

(29) Acute inflammation is associated with lower muscle strength, muscle mass and dependency in activities of daily living in male hospitalised older patients. AUSTRALASIAN JOURNAL ON AGEING: WILEY 111 RIVER ST, HOBOKEN 07030-5774, NJ USA; 2018.

(30) Hao Q, Zhou L, Dong B, Yang M, Dong B, Weil Y. The role of frailty in predicting mortality and readmission in older adults in acute care wards: a prospective study. Scientific reports 2019;9(1):1207.

(31) Martínez-Velilla N, Casas-Herrero A, Zambom-Ferraresi F, de Asteasu, Mikel L Sáez, Lucia A, Galbete A, et al. Effect of exercise intervention on functional decline in very elderly patients during acute hospitalization: a randomized clinical trial. JAMA internal medicine 2019;179(1):28-36.

(32) Atkinson G, Reilly T. Circadian variation in sports performance. Sports medicine 1996;21(4):292-312.

(33) Ortlipp M. Keeping and using reflective journals in the qualitative research process. The qualitative report 2008;13(4):695-705.

(34) Kahn RL, Cannell CF. The dynamics of interviewing; theory, technique, and cases. 1957.

(35) Smith JA, Osborn M. Interpretative phenomenological analysis. Doing social psychology research 2004:229-254.

(36) Roberts HC, Denison HJ, Martin HJ, Patel HP, Syddall H, Cooper C, et al. A review of the measurement of grip strength in clinical and epidemiological studies: towards a standardised approach. Age Ageing 2011 07/01/;40(4):423-429.

(37) Haff GG, Triplett NT. Essentials of strength training and conditioning 4th edition. : Human kinetics; 2015.

For peer review only

(38) Epley B. Poundage chart. Boyd Epley Workout.Lincoln, NE: Body Enterprises, 2985 1985;86:p. 86.

(39) Guralnik JM, Simonsick EM, Ferrucci L, Glynn RJ, Berkman LF, Blazer DG, et al. A short physical performance battery assessing lower extremity function: association with self-reported disability and prediction of mortality and nursing home admission. J Gerontol 1994;49(2):M8-M94.

(40) Katz S, Chinn AB, Cordrey L. Multidisciplinary studies of illness in aged persons II: a new classification of functional status in activities of daily living. J Chronic Dis 1959;9(1):55-62.

(41) Yesavage JA, Brink TL, Rose TL, Lum O, Huang V, Adey M, et al. Development and validation of a geriatric depression screening scale: a preliminary report. J Psychiatr Res 1983;17(1):37-49.

(42) Zigmond AS, Snaith RP. The hospital anxiety and depression scale. Acta Psychiatr Scand 1983;67(6):361-370.

(43) Molloy DW, Alemayehu E, Roberts R. Reliability of a standardized mini-mental state examination compared with the traditional mini-mental state examination. Am J Psychiatry 1991;148(1):102-105.

(44) Cohen S, Mermelstein R, Kamarck T, Hoberman HM. Measuring the functional components of social support. Social support: Theory, research and applications: Springer; 1985. p. 73-94.

(45) Heaney JL, Carroll D, Phillips AC. DHEA, DHEA-S and cortisol responses to acute exercise in older adults in relation to exercise training status and sex. Age 2013;35(2):395-405.

(46) Córdova C, Lopes-e-Silva J, Pires AS, Souza VC, Brito CJ, Moraes CF, et al. Long-Term Resistance Training Is Associated with Reduced Circulating Levels of IL-6, IFN-Gamma and TNF-Alpha in Elderly Women. Neuroimmunomodulation 2011 /02/09;18(3):165-170.

(47) Smart NA, Larsen AI, Le Maitre JP, Ferraz AS. Effect of exercise training on interleukin-6, tumour necrosis factor alpha and functional capacity in heart failure. Cardiology research and practice 2011;2011.

(48) Fischer CP. Interleukin-6 in acute exercise and training: what is the biological relevance. Exerc Immunol Rev 2006;12(6-33):41.

(49) Petersen AMW, Pedersen BK. The anti-inflammatory effect of exercise. J Appl Physiol 2005;98(4):1154-1162.

For peer review only

(50) Goldhammer E, Tanchilevitch A, Maor I, Beniamini Y, Rosenschein U, Sagiv M. Exercise training modulates cytokines activity in coronary heart disease patients. Int J Cardiol 2005;100(1):93-99.

(51) Wilkinson MD, Dumontier M, Aalbersberg IJ, Appleton G, Axton M, Baak A, et al. The FAIR Guiding Principles for scientific data management and stewardship. Scientific data 2016;3.

(52) Morse JM. Designing funded qualitative research. 1994.

(53) Creswell JW, Poth CN. Qualitative inquiry and research design: Choosing among five approaches. : Sage publications; 2017.

(54) United Kingdom. Department of Health. Guidance on nominating a consultee for research involving adults who lack capacity to consent (8953). URL: https://webarchive.nationalarchives.gov.uk/20130123193236/http://www.dh.gov.uk/en/Publicationsandstatistics/Publications/PublicationsPolicyAndGuidance/DH_083131. Accessed: 5th July 2019. 2008.

(55) United Kingdom Mental Capacity Act, 2005. URL: https://www.legislation.gov.uk/ukpga/2005/9/section/32. Accessed 5th July 2019.

(56) Whittaker AC, Delledonne M, Finni T, Garagnani P, Greig C, Kallen V, et al. Physical Activity and Nutrition INfluences In ageing (PANINI): consortium mission statement. Aging clinical and experimental research 2018:1-8.

(57) University of Birmingham. Physical Activity and Nutritional INfluences In ageing (PANINI)  Available at: http://www.birmingham.ac.uk/generic/panini/index.aspx. Accessed 6th February, 2019.

(58) Doody P, Lord JM, Whittaker AC. Assessing the feasibility and impact of an adapted resistance training intervention, aimed at improving the multi-dimensional health and functional capacity of frail older adults in residential care settings: protocol for a feasibility study. Pilot and Feasibility Studies 2019;5(1):86.

For peer review only

Protocol_Manscript_V3.0_02/09/2019

616 # Tables

617 *Table 1. The eight primary areas of focus, outlining the research questions and methods of assessment*

| Area of focus | Potential questions | Methods of Assessment |
|---|---|---|
| Acceptability | • Will the proposed population be interested in participating in the study?<br>• What will the uptake be?<br>• Will the program be judged as suitable by the delivers of the program in addition to the program participants?<br>• Participant's opinions on hypothetically being randomized into a control group during a proposed future clinical trial? *<br><br>*Participants within the feasibility study will not be recruited as participants within the proposed future clinical trial in order to protect the scientific validity of the proposed future clinical trial, as the participants within the feasibility will already have undergone the interventions, or similar interventions. However, participants within the feasibility study will likely not still be residents on the ward, as the average stay for participants on the ward is approximately 3.5 weeks. As such once the proposed future clinical trial has commenced, all participants within the feasibility trial will likely have left the ward well in advance. | • Participant uptake analysis (All participants approached and eligible for the study, all of those successfully recruited to the study)<br>• Semi-structured interviews with participants<br>• Focus groups with study support staff. |
| Demand | • Will the proposed population of hospital inpatients participate in the study?<br>• What will adherence rates be?<br>• Are the staff on the ward open to the idea of having exercise interventions potentially on the ward long term if it proves effective? | • Analysis of uptake rates<br>• Exercise intervention adherence rates<br>• Focus groups with study support staff/ward staff. |
| Implementation | • What are the possible logistical issues with the setting which will need to be addressed or accounted for prior to the clinical trial?<br>• Can the interventions be successfully carried out within this setting?<br>• Can a single or double bind be successfully implemented within this setting? | • Semi-structured interviews with study participants.<br>• More in-depth with focus groups with study support staff. |
| Practicality | • What are the practical implications of the study with relation to time commitment of the researchers, relating to both the implementation of the interventions, and the testing of participants for the dependent variables of the proposed future clinical trial?<br>• Is it viable to potentially conduct follow-up testing on participants in the proposed future clinical trial? | • Semi-structured interviews with study participants.<br>• Focus groups with support staff. |



For peer review only - http://bmjopen.bmj.com/site/about/guidelines.xhtml

Protocol_Manscript_V3.0_02/09/2019

| | | |
|---|---|---|
| | • Do any alterations need to be made to the proposed primary dependent variables of the future clinical trial?<br>• If the interventions are successful in influencing parameters of health and functional capacity, will it potentially be possible to assess if these improvements are sustained during a two week follow up in the proposed future clinical trial, if the same is found? | |
| Integration | • How will the ward staff appraise the study?<br>• Will the interventions be easily integrated into the existing culture, protocols and procedures within the ward seamlessly? | • Focus groups with ward/support staff. |
| Adaptation | • Will any further adaptations be required to the existing interventions to make them more feasible or appropriate within this setting? | • Semi structured interviews with participants. |
| Expansion | • Can the Move It or Lose It intervention (an established chair-based exercise programme for older adults) be successfully expanded to this setting?<br>• Can be specially adapted resistance training equipment be successfully expanded to this setting? | • Semi-structured interviews / Focus group with the ward/study support staff. |
| Limited-efficacy testing | • Is two weeks a sufficient duration to potentially provide significant benefit to patients?<br>• Can intensive (five-six days per week), short duration (two weeks) physical activity interventions improve markers of multi-dimensional health, in very frail individuals? | • Analysis of the secondary dependent variables within the study (primary dependant variables of the future clinical trial)<br>• Analysis of uptake and adherence rates<br>• Analysis of the level of satisfaction with the interventions through questionnaires with participants post intervention. |

For peer review only

621 *Table 2. Study timeline of all major events during each round of recruitment (SPIRIT Schedule).*

| Week | Seated Physical Activity in Ageing (SPAA) Study Timeline | | | | | | |
|---|---|---|---|---|---|---|---|
| | Monday | Tuesday | Wednesday | Thursday | Friday | Saturday | Sunday |
| Week 0 | Identification + participant information sheet distribution | 24-hour consideration period | Recruitment, eligibility screening, and pre-intervention assessments | Recruitment, eligibility screening, and pre-intervention assessments | Recruitment, eligibility screening, and pre-intervention assessments | Recruitment, eligibility screening, and pre-intervention assessments | - |
| Week 1 | Training | Rest | Training | Training | Rest | Training | Training |
| Week 2 | Training | Rest | Training | Training | Training | Rest | Training |
| Week 3 | Post-Intervention Assessment | Post-Intervention Assessment | Post-Intervention Assessment | Post-Intervention Assessment | Post-Intervention Assessment | Post-Intervention Assessment | - |

For peer review only

623 **Figures**

624 Figure 1. Trial schema of participant flow throughout the duration of the study.

625 Figure 2. The independent variables of the proposed future clinical trial.

626 Figure 3. Minato resistance training equipment utilised within the specially adapted resistance training
627 intervention.

628 Figure 4. Exercise intervention 1- Specially adapted resistance training intervention protocol.

629 Figure 5. Exercise intervention 2 – Move It Or Lose It (MIOLI) chair-based exercise intervention protocol.

630 All figures are attached to this manuscript submission as separate documents titled "Figure 1", "Figure 2",
631 "Figure 3", "Figure 4" and "Figure 5" accordingly.

632

For peer review only



For peer review only - http://bmjopen.bmj.com/site/about/guidelines.xhtml

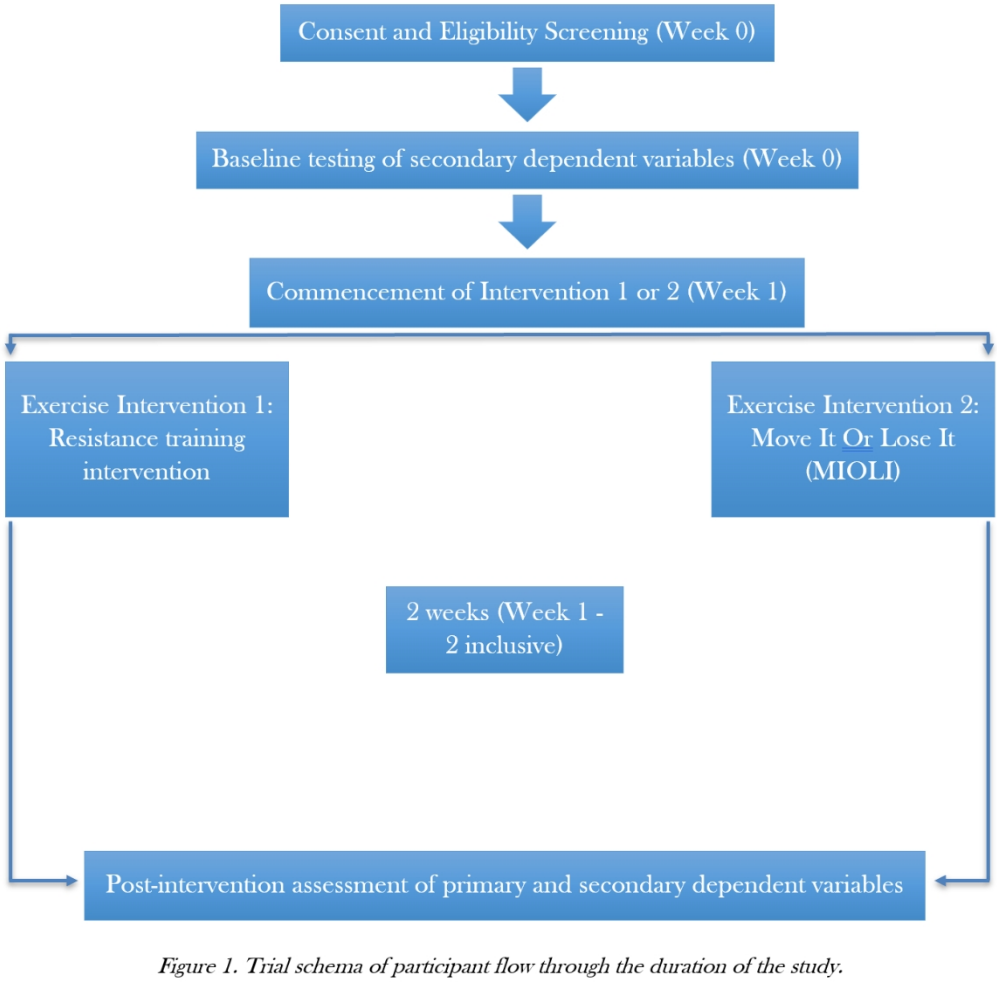


*Figure 1. Trial schema of participant flow through the duration of the study.*

Figure 1. Trial schema of participant flow through the duration of the study.

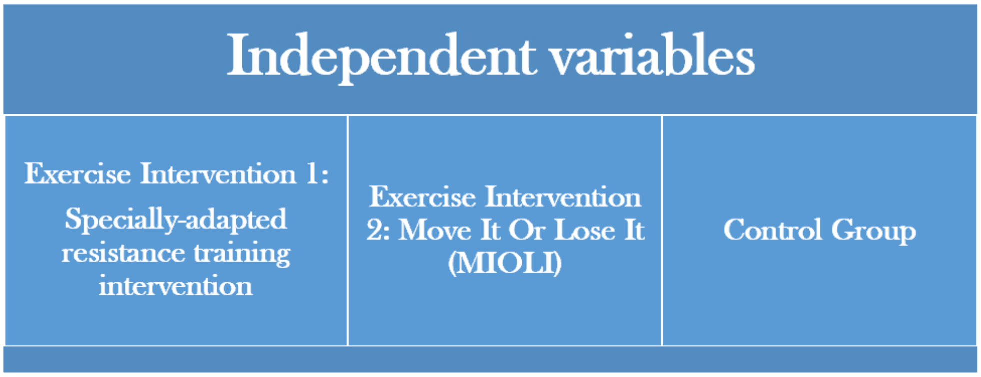


*Figure 2. The independent variables of the proposed future clinical trial.*

Figure 2. The independent variables of the proposed future clinical trial.

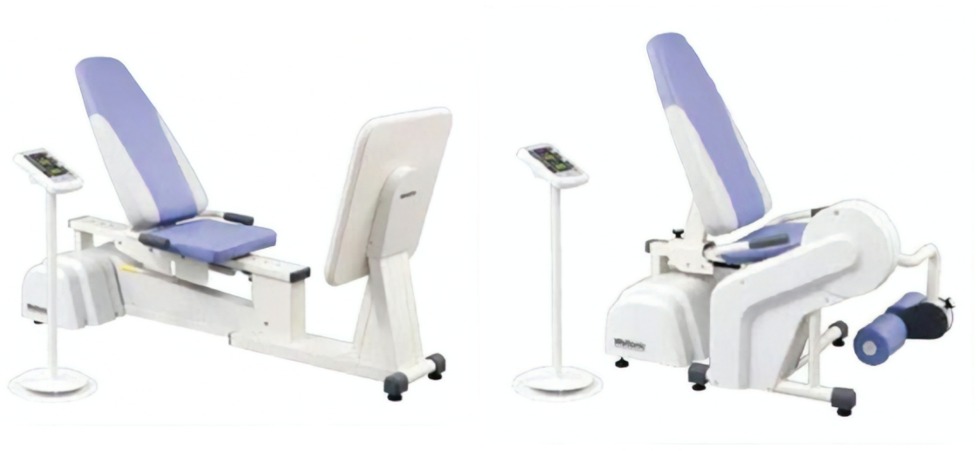

*Figure 3. Minato resistance training equipment utilised within the specially adapted resistance training intervention*

Figure 3. Minato resistance trianing equipment utilised within the speically adapted resistence training intervention.

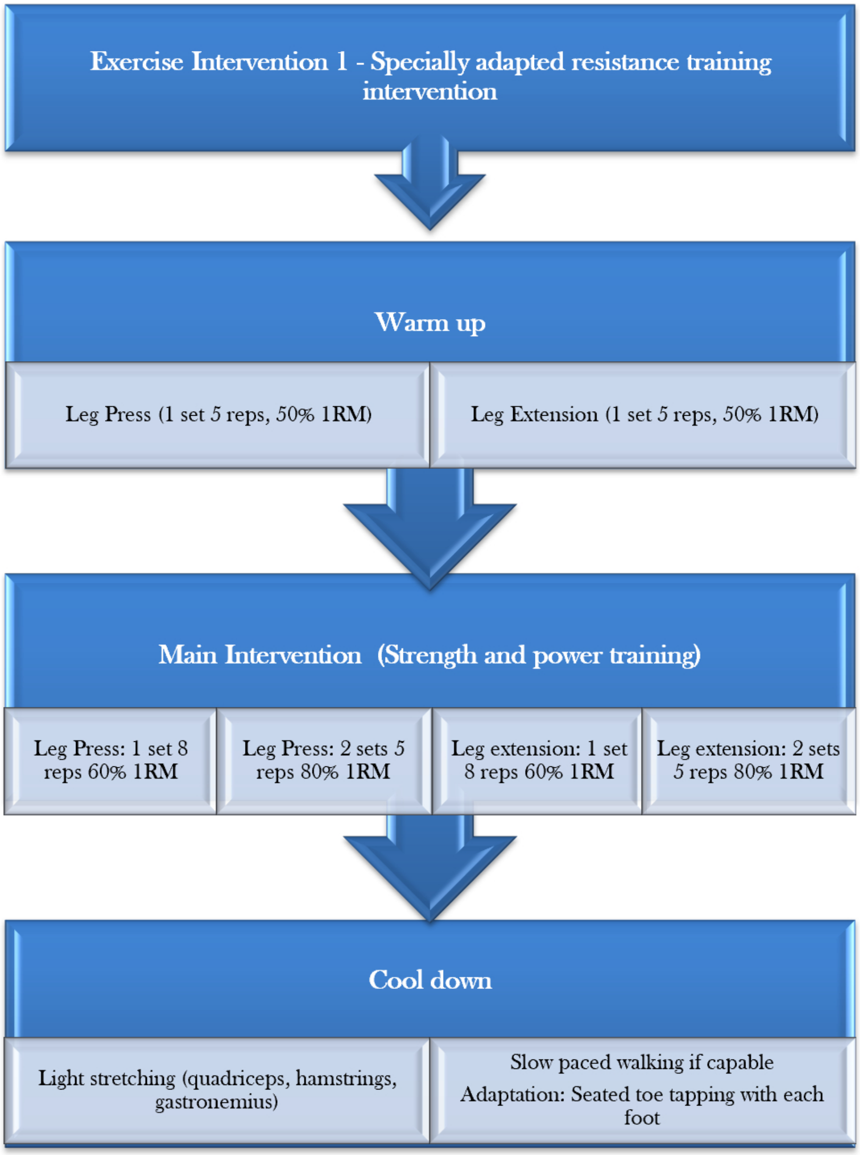


*Figure 4. Exercise intervention 1 – Specially adapted resistance training intervention protocol.*

Figure 4. Exercise intervention 1 – Specially adapted resistance training intervention protocol.

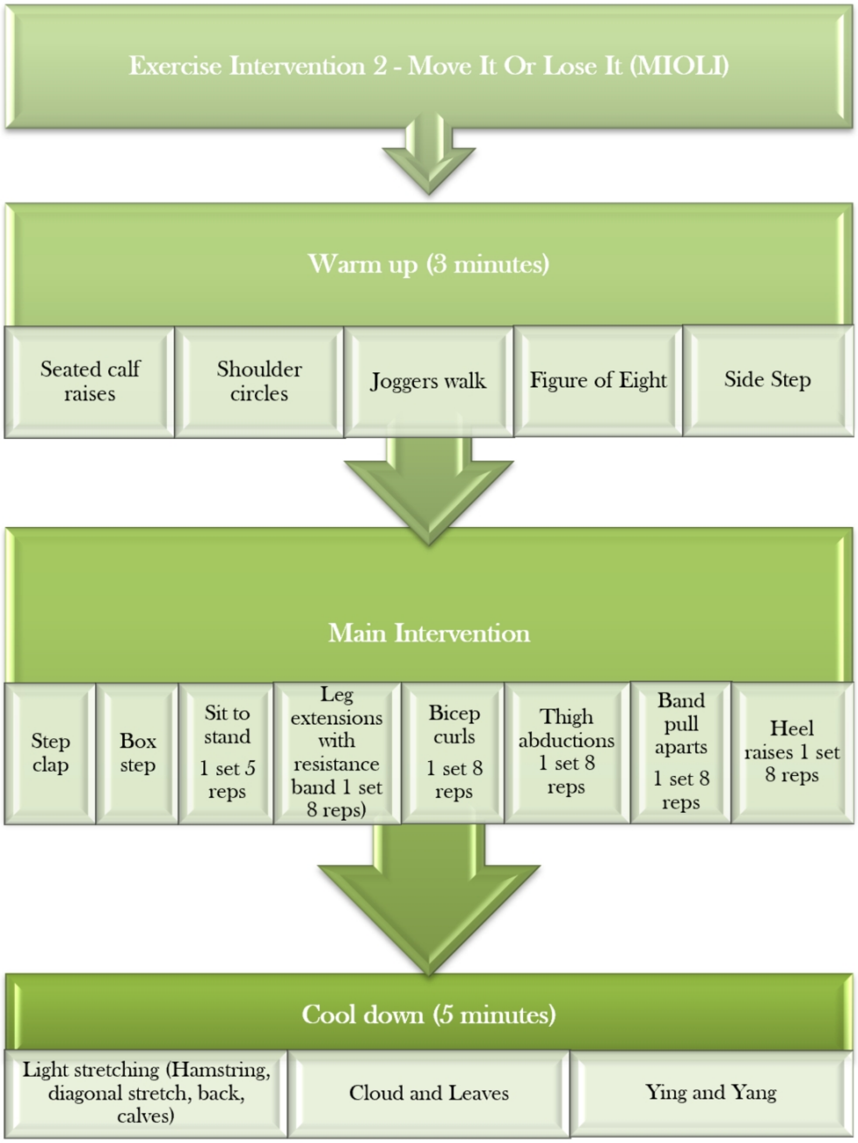


*Figure 5. Exercise Intervention 2 – Move It Or Lose It (MIOLI) chair-based exercise intervention protocol.*

Figure 5. Exercise Intervention 2 - Move It Or Lose It (MIOLI) chair-based exercise intervention protocol.

# Leg Strength Assessment

Leg strength will be assessed by an estimated one repetition maximum (1RM) obtained from participants five repetition maximum (5RM) on the leg press and leg extension machines pre- and post-intervention. The protocol for this assessment can be found below:

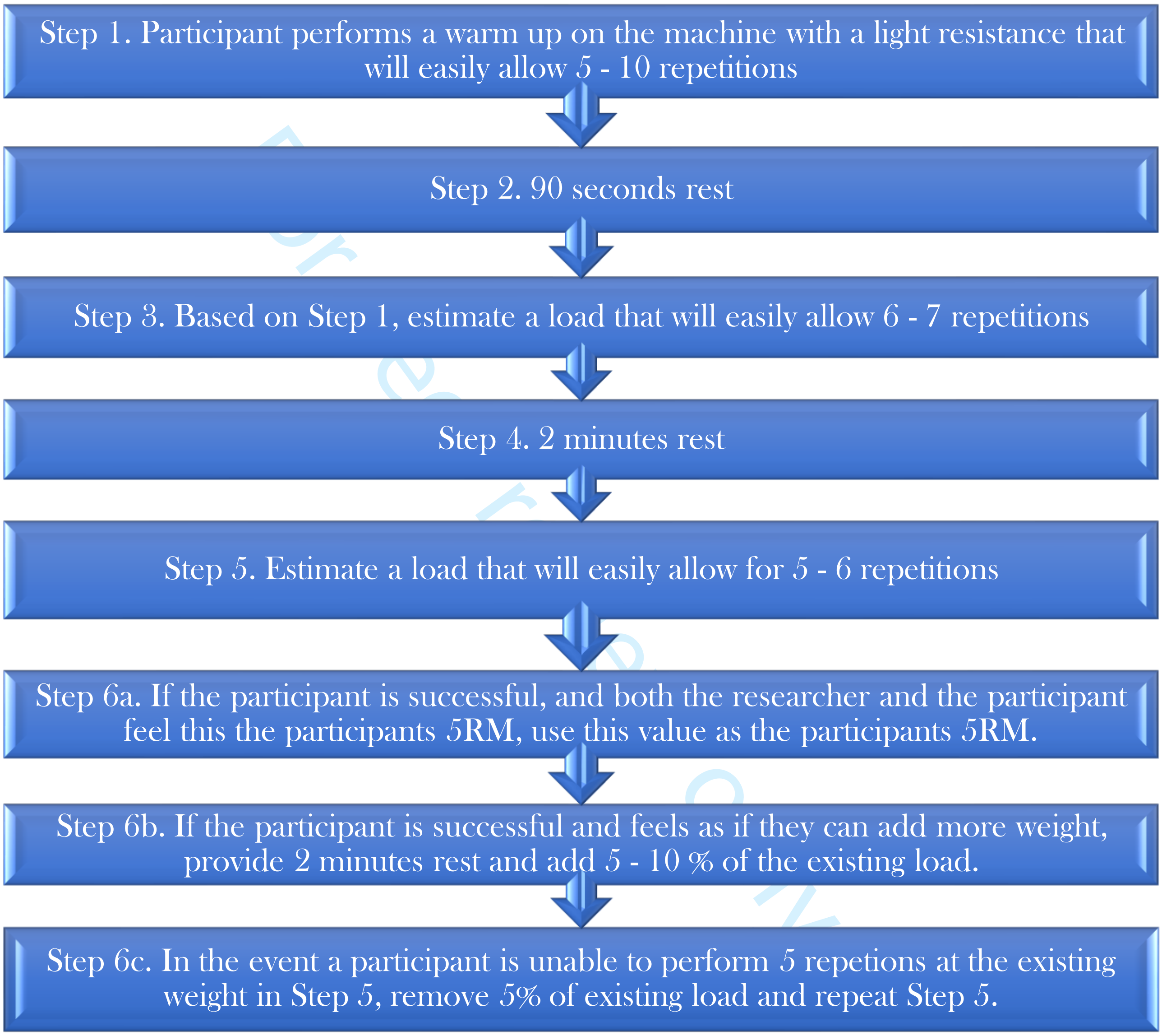


*Supplementary Material Figure 1: Five repetition maximum (5RM) assessment protocol adapted from Haff, G Gregory, Triplett, 2015*

For peer review only - http://bmjopen.bmj.com/site/about/guidelines.xhtml

# Seated Physical Activity in Ageing (SPAA)

## Participant Information Sheet

## I. What is this study about?

This research study will assess whether it is feasible to conduct two seated physical activity interventions for frail older adults within a short-stay hospital ward setting. The intervention will run five days per week (35 mins per session) for two weeks. We are interested in the effects on health and wellbeing.

The specially-adapted chair-based physical activity interventions are:

**<u>Move It Or Lose It (MIOLI)</u>**

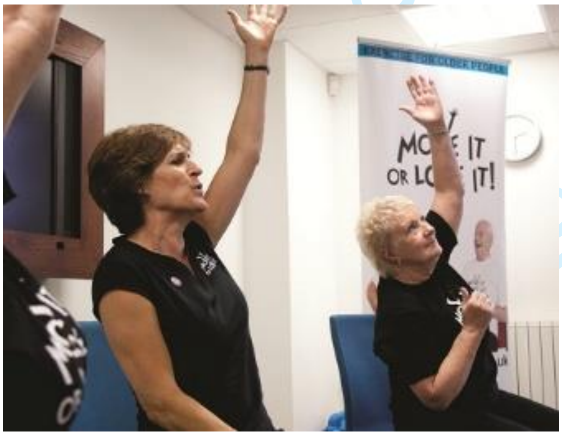

- An established chair-based physical activity programme for older adults.
- Exercises performed will be related to strength, balance, fitness and flexibility.
- The exercises are aimed to increase function and independence in older adults
- All exercises performed are chair-based, so they can be performed while sitting or standing (with, or without, chair support).

**<u>Resistance Training Intervention</u>**

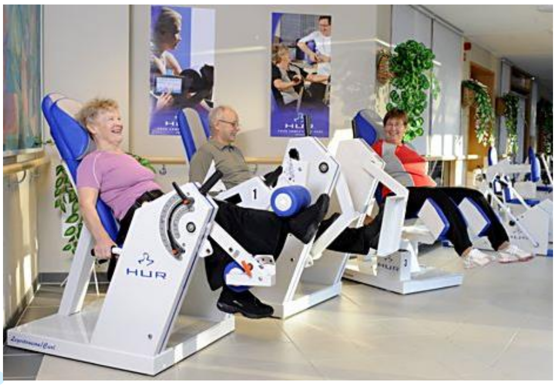


- Specially adapted resistance training machines for older adults.
- They are comfortable, easy to use, and are pneumatic (work on air-based resistance)
- They allow the difficulty to be increased in small amounts (100 grams - similar to the weight of a small apple).
- The machines work by scanning your wristband on a touch screen, and pressing buttons to increase or decrease difficulty

## II. Who is organising and conducting this study?

The Physical Activity and Nutritional INfluences In ageing (PANINI) project research group at the University of Birmingham (School of Sport Exercise and Rehabilitation Sciences), in collaboration with the Queen Elizabeth Hospital Birmingham.

Research Team: Mr. Paul Doody (Email: p.d.doody@bham.ac.uk. Phone: 0121 414 4125)
Professor Anna Whittaker (Email: a.c.whittaker@bham.ac.uk. Phone: 0121 414 4121)
Professor Janet Lord (Email: j.m.lord@bham.ac.uk. Phone: 0121 371 3234)
Dr. Thomas Jackson PhD (Consultant Geriatrician) (Email: t.jackson@bham.ac.uk)
Dr. Zoe Wyrko (Consultant Geriatrician (Email: zoe.wyrko@uhb.nhs.uk)


For peer review only - http://bmjopen.bmj.com/site/about/guidelines.xhtml

## III. Why have I been approached?

The Harborne ward is actively involved in research in geriatric populations, and encourages patient involvement in research to improve care.

In order to be eligible for the study, you must be:

- A patient / resident on the Harborne Ward
- ≥ 65 years old,
- Have the capacity to speak and read in English,
- Not currently terminally ill
- Not currently taking part in any other research study which could affect the findings of this present study
- Frail* (Possessing any three of the following criteria):
  - Low levels of handgrip strength,
  - Unintentional weight loss,
  - Low levels of physical activity,
  - Slow walking speed, and
  - Self-reported exhaustion

*If you would like to participate but are unsure if you are eligible based on the criteria above, do not worry, as these criteria will be assessed by the research team prior to enrolment once you have expressed your interest.

We will also liaise with your clinical care team to determine if you will be expected to stay on the ward for a length of time that would enable your participation in the study, and to ensure that you do not have any condition which could potentially exclude you from the study, such as severe sensory impairments.

## IV. What will happen to me if I take part?

Your participation will be up to 3 weeks, including testing before and after the physical activity interventions themselves. There will be no changes to your standard care.

<u>Before the intervention:</u> Up to four days before the intervention, you will be asked to undergo a series of measurements including testing if you are eligible for the study (1 hour), relating to your physical, psychological, emotional and social health and physical ability. These will be assessed through tests, questionnaires, and a blood sample consisting of three small tubes (16ml total) to measure immune function. These will take about 3 hours in total, across two 1 hour 30-minute sessions over the four days. Although we would ideally like you to complete all of the assessments, if you feel it is too much, we will prioritise certain tests.

<u>During the intervention:</u> You will then be allocated into one of the two physical activity interventions (which intervention depends on the dates you are resident in the ward). You will be asked to complete 10 training sessions throughout two weeks within the physical activity intervention you have been allocated to. Each training session will last approximately 35 minutes, and all training sessions will take place on the Harborne ward.

<u>After the Intervention:</u> After the intervention, you will be asked to complete the same measurements as before (tests of function, questionnaires, and a blood sample) in order to assess the impact of the intervention, which again will be split into two sessions, each about 1 hour 30 minutes. You will also be asked to take part in an interview (20 minutes) with the researcher, audio-recorded with your permission. During this you will be able


For peer review only - http://bmjopen.bmj.com/site/about/guidelines.xhtml

to tell us your opinion of the intervention, what you enjoyed about it the study, and where it could be improved, which will allow us to adapt the intervention based on your feedback, which we would greatly appreciate. After transcription of the interviews the audio recording will be destroyed. The researchers within this study will also request limited access to your medical notes to allow the research team to assess any factors (injuries, medications, diseases) which might affect the results of the study. Information from your patient notes will be stored confidentially and separately from your personal details.

## V. Do I have to take part?

Involvement is purely voluntary and you are free to withdraw from the study at any point without giving an explanation. There will be no negative consequences of withdrawing from the study in terms of your care. If you withdraw we would like to still analyse the data we have collected thus far.

If you express your interest in the study, a member of the research team will then meet with you to provide you with more information on the study, and to answer any questions you may have. If you states that you would not like to be involved in the research and would also not like to be contacted in the future with relation to participation, then you will not be approached by the researchers.

## VI. What are the possible benefits of taking part?

Physical activity has been shown to produce positive health benefits in older people. Additionally, physical activity interventions have been suggested as potentially offering the best form of treatment for frail older adults. Frailty can have an enormous impact on an individual's life, in addition to the lives of their friends and family. If this current study proves feasible and is found to have a positive impact, this research has the potential to have positive implications in the advancement of the hospital care of frail older adults.

## VII. What are the possible risks/side effects of taking part?

Physical activity in older people has a relatively small risk of adverse effects, typically mild muscle soreness. Due to the nature of the interventions (physical activity), it is possible that you may experience mild muscle soreness associated with physical activity, however this is a normal adaptation to exercise. In order to minimise the risk of Delayed Onset Muscle Soreness (DOMS – a more severe form of muscle soreness with a delayed onset), the interventions have been developed in a progressive manner. Additionally, the interventions will be carried out on the Harborne ward; with your care team in close proximity at all times.

## VIII. Confidentiality and data protection

Your identity or other personal information will be kept confidential. You will be assigned an ID number under which all study information will be stored on an encrypted and password protected computer/laptop at the University of Birmingham. Physical data (e.g. questionnaires) will be identifiable only by ID number and stored in a locked filing cabinet at the University of Birmingham, accessible only by the research team. Your personal information (name, D.O.B.) and your consent form will be stored securely in a separate locked filing cabinet. Data will be stored securely and confidentially for 10 years and anonymized data will also be entered into a European 'PANINI' database that this project is part of. Blood samples will be stored in approved facilities at the University of Birmingham for three years and **optionally** may be analysed in future ethically approved research across the PANINI network. As part of the PANINI project we would like to send the smallest of the three blood sample tubes to the University of Bologna in Italy to be tested by specialists there for any potential alterations in genetic markers from the physical activity intervention (optional). The University of Birmingham has public liability insurance covering its research.



For peer review only - http://bmjopen.bmj.com/site/about/guidelines.xhtml

## IX. Funding

This study has been funded by the European Commission as part of a European – wide innovate training network (ITN), consisting of Marie Curie Research Fellows / PhD students.

## X. What next?

If you say that you are interested in taking part in the study to your clinical care team, the research team will come to answer any questions you may have, check you are eligible and enrol you into the study. If you do not state that you are either interested/not in the study, the research team will ask you about whether or not you would be potentially interested in taking part. If you would not like to take part, then please tell your care team and/or the research team and you will not be contacted further about participation.

If you agree to take part, we will ask you to sign an informed consent form, outlining that you have read this information sheet and that you are aware of what the study involves. You can either sign this straight away, or take up to 24 hours to make up your mind. After this, you will be assessed for the eligibility criteria, and then begin the testing as soon as possible and then start the intervention.

If you have concerns about this study please contact the study research team or your care team to discuss this. For independent advice, you can contact your local Patient Advice and Liaison Service (PALS) (0121 371 3280), or the study sponsor (the University of Birmingham, who have given this study the sponsor registration number: RG_16-172):

Dr. Sean Jennings, Head of Research Ethics and Governance, University of Birmingham, Edgbaston, Birmingham, United Kingdom. Telephone: 0121 415 8011, Email: s.jennings@bham.ac.uk

Thank you very much for your help

For peer review only



For peer review only - http://bmjopen.bmj.com/site/about/guidelines.xhtml

Informed Consent Form
Seated Physical Activity in Ageing (SPAA)
PANINI (Physical Activity and Nutritional INfluences In ageing) Project

UNIVERSITY OF BIRMINGHAM

# Seated Physical Activity in Ageing (SPAA)

## II. Consent

Please complete the following table. Read each of the following statements. If you agree to the statement please put your **initials** in the box. If you do not agree to the statement please leave the box blank. Please note any boxes which are not marked as optional, are compulsory for initial enrollment into the study.

| | | |
|---|---|---|
| I confirm that I have read the Participant Information Sheet (Version 4 – 10/01/2018), and that I fully understand the information provided, and have had the opportunity to ask questions, and have received satisfactory answers. | | |
| I understand that I will receive a unique ID number, under which all information collected about me will be stored confidentially, either on an encrypted and password protected laptop and computer, or in the case of physical information, within a locked filing cabinet, at the University of Birmingham, accessible only to the research team. | | |
| I consent to giving a blood sample both pre- and post-intervention. I am aware that samples will be stored in a secure laboratory freezer at the University of Birmingham. | | |
| I consent to giving information related to my personal data (name, age, occupation, marital status) psychological, cognitive, emotional, social health and functional capacity through questionnaires and tests. | | |
| I agree to the audio-taping of an interview between myself and the researcher (where I can give my opinion on the study) and understand recordings will be destroyed after transcription. | | |
| I understand that my data might be used in a written report, but will not be identifiable as me. | | |
| I consent to all data obtained about me being stored for a period of 10 years as part of the PANINI project shared dataset, and that these data will be unidentifiable as mine but may be used in future ethically approved research. After 10 years it will be destroyed. | | |
| I understand that involvement within this research project is purely voluntary and that I am free to withdraw from the study at any point which I wish to do so with no repercussion of any form for myself, or my quality of care. | | |
| I understand that relevant sections of the data collected about me during the study may be looked at by ethics regulatory authorities or appropriate individuals from the University of Birmingham during a potential monitoring visit. I give permission for these individuals to have access to my linked anonymized data. | | |
| I consent to take part in this above titled research study of my own volition | | |
| **Optional (Please circle yes / no)** - I consent to members of the research team having limited access to my medical notes in order to allow information to be obtained relating to various morbidities (injuries, ailments, diseases) and medications. | Yes<br>No | |
| **Optional (Please circle yes / no)** - I consent to my blood samples being stored as part of the PANINI project shared dataset for three years, and that these data will be unidentifiable as mine, but may be used in future ethically approved research. | Yes<br>No | |

**Participants' Signature:** ___________________

**Name in Block Capitals:** _________________

**Date:** ________________________________

**Signature of person taking consent:** _______________

**Name in Block Capitals:** ________________________

**Date:** ________________________________________


For peer review only - http://bmjopen.bmj.com/site/about/guidelines.xhtml

SPIRIT — Standard Protocol Items: Recommendations for Interventional Trials

SPIRIT 2013 Checklist: Recommended items to address in a clinical trial protocol and related documents*

| Section/item | Item No | Description | Addressed on page number |
|---|---|---|---|
| **Administrative information** | | | |
| Title | 1 | Descriptive title identifying the study design, population, interventions, and, if applicable, trial acronym | Page 1 (Lines 1-5) |
| Trial registration | 2a | Trial identifier and registry name. If not yet registered, name of intended registry | Page 2 (Line 36), Page 18 (Lines 417) |
| | 2b | All items from the World Health Organization Trial Registration Data Set | N/A |
| Protocol version | 3 | Date and version identifier | Page 1 -28 (Header) |
| Funding | 4 | Sources and types of financial, material, and other support | Page 18 (Lines 423 - 427) |
| Roles and responsibilities | 5a | Names, affiliations, and roles of protocol contributors | Page 1 (Lines 6 -1 – 13), Page 19 (Lines 429 - 432) |
| | 5b | Name and contact information for the trial sponsor | Page 17 (Lines 394 - 396) |
| | 5c | Role of study sponsor and funders, if any, in study design; collection, management, analysis, and interpretation of data; writing of the report; and the decision to submit the report for publication, including whether they will have ultimate authority over any of these activities | N/A |

For peer review only

| | | | |
|---|---|---|---|
| | 5d | Composition, roles, and responsibilities of the coordinating centre, steering committee, endpoint adjudication committee, data management team, and other individuals or groups overseeing the trial, if applicable (see Item 21a for data monitoring committee) | Page 12 (Lines 274 – 280) |
| **Introduction** | | | |
| Background and rationale | 6a | Description of research question and justification for undertaking the trial, including summary of relevant studies (published and unpublished) examining benefits and harms for each intervention | Page 3-5 (Lines 46 – 96) |
| | 6b | Explanation for choice of comparators | Page 4/5 (Lines 67 - 96) |
| Objectives | 7 | Specific objectives or hypotheses | Page 5 (Lines 98 - 114) |
| Trial design | 8 | Description of trial design including type of trial (eg, parallel group, crossover, factorial, single group), allocation ratio, and framework (eg, superiority, equivalence, noninferiority, exploratory) | Page 6/7 (Lines 116 - 153) |
| **Methods: Participants, interventions, and outcomes** | | | |
| Study setting | 9 | Description of study settings (eg, community clinic, academic hospital) and list of countries where data will be collected. Reference to where list of study sites can be obtained | Page 4 (Line 76), Page 5 (Lines 106 - 107), Page 6 (Lines 121 - 123) |
| Eligibility criteria | 10 | Inclusion and exclusion criteria for participants. If applicable, eligibility criteria for study centres and individuals who will perform the interventions (eg, surgeons, psychotherapists) | Page 7 (Lines 153 - 163) |
| Interventions | 11a | Interventions for each group with sufficient detail to allow replication, including how and when they will be administered | Page 7/8 (Lines 165 - 182) |
| | 11b | Criteria for discontinuing or modifying allocated interventions for a given trial participant (eg, drug dose change in response to harms, participant request, or improving/worsening disease) | Page 17/18 Lines 403 - 414) |


For peer review only - http://bmjopen.bmj.com/site/about/guidelines.xhtml

| | | | |
|---|---|---|---|
| | 11c | Strategies to improve adherence to intervention protocols, and any procedures for monitoring adherence (eg, drug tablet return, laboratory tests) | Page 12 (Lines 266 - 272) |
| | 11d | Relevant concomitant care and interventions that are permitted or prohibited during the trial | Page 6/7 Lines 125 - 153) |
| Outcomes | 12 | Primary, secondary, and other outcomes, including the specific measurement variable (eg, systolic blood pressure), analysis metric (eg, change from baseline, final value, time to event), method of aggregation (eg, median, proportion), and time point for each outcome. Explanation of the clinical relevance of chosen efficacy and harm outcomes is strongly recommended | Page 8-11 (Lines 184 - 248) |
| Participant timeline | 13 | Time schedule of enrolment, interventions (including any run-ins and washouts), assessments, and visits for participants. A schematic diagram is highly recommended (see Figure) | Page 11/12 (Lines 250 - 272) |
| Sample size | 14 | Estimated number of participants needed to achieve study objectives and how it was determined, including clinical and statistical assumptions supporting any sample size calculations | Page 12/13 (Lines 282 - 286) |
| Recruitment | 15 | Strategies for achieving adequate participant enrolment to reach target sample size | Page 13/14 (Lines 288 - 316) |

**Methods: Assignment of interventions (for controlled trials)**

Allocation:

| | | | |
|---|---|---|---|
| Sequence generation | 16a | Method of generating the allocation sequence (eg, computer-generated random numbers), and list of any factors for stratification. To reduce predictability of a random sequence, details of any planned restriction (eg, blocking) should be provided in a separate document that is unavailable to those who enrol participants or assign interventions | Page 7 Lines 148 - 149) |
| Allocation concealment mechanism | 16b | Mechanism of implementing the allocation sequence (eg, central telephone; sequentially numbered, opaque, sealed envelopes), describing any steps to conceal the sequence until interventions are assigned | N/A |
| Implementation | 16c | Who will generate the allocation sequence, who will enrol participants, and who will assign participants to interventions | N/A |

| | | | |
|---|---|---|---|
| Blinding (masking) | 17a | Who will be blinded after assignment to interventions (eg, trial participants, care providers, outcome assessors, data analysts), and how | N/A |
| | 17b | If blinded, circumstances under which unblinding is permissible, and procedure for revealing a participant's allocated intervention during the trial | N/A |
| **Methods: Data collection, management, and analysis** | | | |
| Data collection methods | 18a | Plans for assessment and collection of outcome, baseline, and other trial data, including any related processes to promote data quality (eg, duplicate measurements, training of assessors) and a description of study instruments (eg, questionnaires, laboratory tests) along with their reliability and validity, if known. Reference to where data collection forms can be found, if not in the protocol | Page 8 – 12 (Line 194-285) |
| | 18b | Plans to promote participant retention and complete follow-up, including list of any outcome data to be collected for participants who discontinue or deviate from intervention protocols | N/A |
| Data management | 19 | Plans for data entry, coding, security, and storage, including any related processes to promote data quality (eg, double data entry; range checks for data values). Reference to where details of data management procedures can be found, if not in the protocol | Page 13 (Line 287-293) |
| Statistical methods | 20a | Statistical methods for analysing primary and secondary outcomes. Reference to where other details of the statistical analysis plan can be found, if not in the protocol | Page 14 – 16 (Lines 331-373) |
| | 20b | Methods for any additional analyses (eg, subgroup and adjusted analyses) | |
| | 20c | Definition of analysis population relating to protocol non-adherence (eg, as randomised analysis), and any statistical methods to handle missing data (eg, multiple imputation) | N/A |
| **Methods: Monitoring** | | | |
| Data monitoring | 21a | Composition of data monitoring committee (DMC); summary of its role and reporting structure; statement of whether it is independent from the sponsor and competing interests; and reference to where further details about its charter can be found, if not in the protocol. Alternatively, an explanation of why a DMC is not needed | Page 13 (Lines 287-293) |
| | 21b | Description of any interim analyses and stopping guidelines, including who will have access to these interim results and make the final decision to terminate the trial | N/A |


For peer review only - http://bmjopen.bmj.com/site/about/guidelines.xhtml

| | | | |
|---|---|---|---|
| Harms | 22 | Plans for collecting, assessing, reporting, and managing solicited and spontaneously reported adverse events and other unintended effects of trial interventions or trial conduct | Page 17/18 (Line 403 - 414) |
| Auditing | 23 | Frequency and procedures for auditing trial conduct, if any, and whether the process will be independent from investigators and the sponsor | N/A |
| **Ethics and dissemination** | | | |
| Research ethics approval | 24 | Plans for seeking research ethics committee/institutional review board (REC/IRB) approval | Page 17 (Line 391 – 396) |
| Protocol amendments | 25 | Plans for communicating important protocol modifications (eg, changes to eligibility criteria, outcomes, analyses) to relevant parties (eg, investigators, REC/IRBs, trial participants, trial registries, journals, regulators) | N/A |
| Consent or assent | 26a | Who will obtain informed consent or assent from potential trial participants or authorised surrogates, and how (see Item 32) | Page 13/14 (Line 300 - 316) |
| | 26b | Additional consent provisions for collection and use of participant data and biological specimens in ancillary studies, if applicable | Page 16 (Lines 376 - 380) |
| Confidentiality | 27 | How personal information about potential and enrolled participants will be collected, shared, and maintained in order to protect confidentiality before, during, and after the trial | Page 16 (Lines 361 - 380) |
| Declaration of interests | 28 | Financial and other competing interests for principal investigators for the overall trial and each study site | Page 19 (Lines 434 - 435) |
| Access to data | 29 | Statement of who will have access to the final trial dataset, and disclosure of contractual agreements that limit such access for investigators | Page 16 (Lines 361 - 380) |
| Ancillary and post-trial care | 30 | Provisions, if any, for ancillary and post-trial care, and for compensation to those who suffer harm from trial participation | N/A |
| Dissemination policy | 31a | Plans for investigators and sponsor to communicate trial results to participants, healthcare professionals, the public, and other relevant groups (eg, via publication, reporting in results databases, or other data sharing arrangements), including any publication restrictions | Page 17 (Lines 387 – 388, Lines 398 – 401) |

For peer review only

| | | | |
|---|---|---|---|
| | 31b | Authorship eligibility guidelines and any intended use of professional writers | Page 19 (Lines 429 - 432) |
| | 31c | Plans, if any, for granting public access to the full protocol, participant-level dataset, and statistical code | Page 16 (Lines 376 – 380) |
| **Appendices** | | | |
| Informed consent materials | 32 | Model consent form and other related documentation given to participants and authorised surrogates | Supplementary files |
| Biological specimens | 33 | Plans for collection, laboratory evaluation, and storage of biological specimens for genetic or molecular analysis in the current trial and for future use in ancillary studies, if applicable | Page 10 (Lines 233 – 235), Page 11 (Lines 243 – 248) |

*It is strongly recommended that this checklist be read in conjunction with the SPIRIT 2013 Explanation & Elaboration for important clarification on the items. Amendments to the protocol should be tracked and dated. The SPIRIT checklist is copyrighted by the SPIRIT Group under the Creative Commons "Attribution-NonCommercial-NoDerivs 3.0 Unported" license.

For peer review only
